# Intermolecular Electrophilic Germylation Using GeCl_4_ and Al_2_Cl_6_

**DOI:** 10.1002/anie.7070836

**Published:** 2026-04-13

**Authors:** Justyna Łosiewicz, Milan Kumar Bisai, Zachary A. Bub, Stuart A. Macgregor, Michael J. Ingleson

**Affiliations:** ^1^ EaStCHEM School of Chemistry University of Edinburgh Edinburgh UK; ^2^ EaStCHEM School of Chemistry North Haugh University of St Andrews St. Andrews UK

**Keywords:** aluminum trichloride, C–H germylation, electrophilic substitution, germanium, Lewis acids

## Abstract

Electrophilic aromatic substitution is a textbook transformation for the lighter Group 14 elements. In contrast to acylation, alkylation, and silylation, electrophilic germylation is extremely underdeveloped. Herein we report the first effective intermolecular Germa–Friedel–Crafts reaction. Specifically, combining the key industrial precursor GeCl_4_, with Al_2_Cl_6_ and an inexpensive hindered pyridine base enables electrophilic C‐H germylation of a range of arenes. The process can be applied for selective synthesis of either the mono‐ or the di‐aryl germanes, ArGeCl_3_ and Ar_2_GeCl_2_, respectively. ArGeCl_3_ are versatile intermediates that can be transformed in situ into the synthetically desirable ArGe(alkyl)_3_ derivatives. Mechanistic and computational analysis support an S_E_Ar‐type process where Al_2_Cl_6_ is the key halophilic activator that generates a germanium electrophile able to effect C–H germylation in combination with the base. Overall, this work demonstrates that a high‐yielding intermolecular Germa–Friedel–Crafts reaction is possible provided an appropriate Brønsted base is used.

## Graphical abstract

While electrophilic aromatic substitution is well‐established for carbon and silicon‐based electrophiles, it is extremely underdeveloped for germanium. Herein we report the first effective intermolecular Germa–Friedel–Crafts reaction. The process can be modified to enable selective synthesis of the mono‐ or the di‐aryl germane derivatives, ArGeCl_3_ and Ar_2_GeCl_2_.

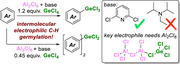

## 1 Introduction

**FIGURE 1:**
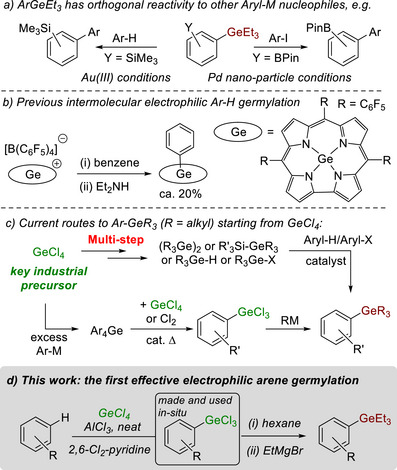
(a) Orthogonal (to other Aryl‐M nucleophiles) reactivity of ArGeEt_3_. (b) A previously reported example of intermolecular electrophilic germylation of arenes. (c) Current multistep routes from GeCl_4_ to ArGeR_3_ reagents. (d) This work on direct electrophilic germylation of arenes using GeCl_4_/AlCl_3_/base.

The past decade has witnessed dramatic advances in germanium chemistry [1], particularly in the synthesis and utilisation of organogermanes [2, 3]. There are now many modular transformations starting from ArGe(alkyl)_3_ derivatives, most commonly ArGeEt_3_ [2, 4]. Furthermore, ArGeEt_3_ nucleophiles have been shown to react in an orthogonal manner to all other aryl‐M nucleophiles (M = Sn, Si, B, Zn, etc.; Figure 1a) which has enabled novel chemoselective diversification strategies [4, 5, 6, 7]. These breakthroughs, coupled with the good stability and low toxicity of ArGe(alkyl)_3_, mean that aryl‐germanes are now sought‐after reagents [2]. This has led in turn to an increased need for efficient methods to form aryl‐germanes [8]. Ideally, these would proceed by C–H germylation of the parent arene. Electrophilic aromatic substitution (S_E_Ar) is a textbook method for the direct functionalisation of arenes. While Friedel–Crafts alkylation and acylation reactions are ubiquitous, S_E_Ar reactions that install the heavier members of group 14 are much less developed. Indeed, it is only in the past decade that notable advances in electrophilic silylation have enabled the Sila‐Friedel–Crafts reaction to become a useful transformation [9, 10, 11, 12, 13]. The effective scavenging of the protic by‐product from S_E_Ar was crucial to enable high‐yielding electrophilic C‐H silylation. In contrast to silylation, the electrophilic germylation of arenes is extremely rare [14, 15]. To our knowledge, intermolecular electrophilic arene C–H germylation is limited to just one report (Figure 1b) [16, 17, 18]. However, this required a square planar germanium electrophile and reported low yielding (ca. 25%) C–H germylation of just a single arene (benzene). The low arene germylation yield has been attributed previously to proto‐degermylation of the aryl‐germane (making germylation reversible), but attempts to sequester the “H^+^” by‐product from S_E_Ar using alkyl amines (Hünig's base or Et_2_NH) did not increase germylation yields [17, 18]. Therefore, developing a methodology for the effective (i.e., achieving good germylation yields using simple reagents) intermolecular electrophilic C‐H germylation of arenes remains a significant unmet challenge.

A particularly attractive precursor for germylation chemistry is GeCl_4_ as it is the only germanium compound directly made from “germanium concentrate” (the industrial starting material) [19]. However, the established route to form organogermanes starting from GeCl_4_ is inefficient (Figure 1c) and requires the use of Grignard or aryl‐lithium reagents (generally derived from aryl‐halides) [20]. Furthermore, even under strict stoichiometric control, this approach leads to mixtures of products (R_x_GeCl_4‐x_) [21, 22, 23]. A direct arene C‐H germylation process using GeCl_4_ would be desirable as an efficient method to form aryl‐germanes. Note, while there are notable non‐S_E_Ar arene C‐H germylation approaches reported [2], these all use more complex and expensive germanium precursors [24] that are actually derived from GeCl_4_.

Herein we report the first effective electrophilic C‐H germylation of arenes. This methodology uses only inexpensive reagents (GeCl_4_, AlCl_3_ and 2,6‐dichloro‐pyridine), proceeds under neat conditions, and can be rationally modified to access Ar_2_GeCl_2_ or ArGeCl_3_ derivatives. Mechanistic studies were consistent with an S_E_Ar‐type process where GeCl_4_ is activated by Al_2_Cl_6_, not AlCl_3_.

## 2 Results and Discussion

**SCHEME 1:**
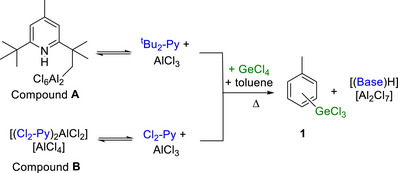
The reversible formation of **A** and **B** and the subsequent formation of germylation product **1** on heating both mixtures.

In previous work on electrophilic arene C‐H borylation using BCl_3_/AlCl_3_/amine, amines such as Et_3_N/Hünig's base were outperformed by hindered pyridines [25, 26, 27, 28, 29]. This is presumably due to the higher nucleophilicity of trialkylamines toward simple (e.g., ligand‐free) Lewis acidic metal(oid)‐halides. Therefore, it was hypothesised that combining GeCl_4_/AlCl_3_ with hindered pyridines could lead to an effective electrophilic C‐H germylation process. Studies commenced using 4‐Me‐2,6‐^t^Bu_2_‐pyridine (termed ^t^Bu_2_‐Py herein) and 2,6‐dichloro‐pyridine (Cl_2_‐Py) as weakly nucleophilic bases that are effective in electrophilic borylation using BCl_3_/AlCl_3_ [25, 26, 27, 28, 29, 30]. Note, while these two bases do react with AlCl_3_ to form **A** and **B**, respectively (Scheme 1) [30, 31], these reactions are reversible.

Initially, equimolar combinations of ^t^Bu_2_‐Py, GeCl_4_ and AlCl_3_ in chlorobenzene were reacted with toluene (selected as a weakly activated S_E_Ar substrate, Mayr *N* value = −4.4) [32, 33]. At temperatures <80°C only sp^3^C‐H alumination of ^t^Bu_2_‐Py was observed, leading to compound **A** (Scheme 1, top) [31]. Repeating the reaction at higher temperatures led to sp^2^C‐H germylation, forming a mixture of *para‐* (major), *meta‐*, and *ortho*‐germylated isomers, termed **1** (Scheme 1). When using one equivalent of all reagents, the yield of **1** was ≤50% under a range of conditions, but when two equivalents of AlCl_3_ were used, **1** was formed in good yield (>80%). A related outcome was observed replacing ^t^Bu_2_‐Py with Cl_2_‐Py (Scheme 1, bottom), with **1** again formed in good conversion on heating. To unambiguously confirm electrophilic germylation had occurred, **1** was converted to the previously reported bench‐stable tolyl‐GeEt_3_ product, termed **2a**. This enabled the isolation of **2a** in good yield.

An optimisation study was performed next, and from this it was found that performing the reaction neat gave good outcomes. As this is desirable (from a green chemistry perspective) [34], all subsequent optimisation reactions were run under neat conditions. Note, while the major germylated product is **1**, low quantities of a species assigned as (tolyl)_2_GeCl_2_ also was observed (*vide infra*), and this combined with unreacted toluene represents the arene mass balance for these reactions. AlCl_3_ and base were both required for germylation (Table 1 entries 1 and 2) [35], with 2 equivalents of AlCl_3_ and 1.2 equivalents of GeCl_4_ resulting in good conversions (entry 3 vs. 4). At lower temperatures the selectivity for the *para*‐germylated isomer improves (entries 5 and 6 vs. 4). However, significant amounts of **A** persisted after 16 h when using ^t^Bu_2_‐Py at these lower temperatures. Therefore, several other amine bases that were effective in the borylation of arenes using BCl_3_/AlCl_3_ were explored (Table S1) [30]. The inexpensive base 2,6‐dichloropyridine (Cl_2_‐py) proved optimal out of those tested. With Cl_2_‐py, lower temperatures also could be used, which improved the selectivity for *para*‐germylated toluene without reducing the conversion to **1** (entries 8 and 9). Note, effectively quantitative [(Cl_2_‐py)H]^+^ is formed (by ^1^H NMR spectroscopy) in reactions forming **1** in high yield, indicating Cl_2_‐py is effectively sequestering the protic by‐product from this S_E_Ar reaction. In contrast, using Hünig's base under the optimised conditions led to <10% germylation (entry 10), highlighting the importance of using an appropriate base.

**TABLE 1:** Select optimisation for AlCl_3_/base‐mediated germylation of toluene.^a^

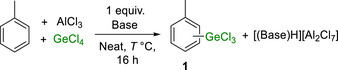					
	AlCl_3_ (eq.)	GeCl_4_ (eq.)	Base	T (°C)	Conversion p/m/o (%)^b^
1	—	1.2	^t^Bu_2_‐Py	120	0/0/0
2	2.0	1.2	—	120	0/0/0
3	2.0	1.2	^t^Bu_2_‐Py	120	58/23/12
4	2.0	1.0	^t^Bu_2_‐Py	120	45/25/9
5	2.0	1.2	^t^Bu_2_‐Py	100	65/16/11
6	2.0	1.2	^t^Bu_2_‐Py	80	52/16/9
7	2.0	1.2	Cl_2_‐Py	100	64/25/8
8	2.0	1.2	Cl_2_‐Py	80	77/14/6
9	2.0	1.2	Cl_2_‐Py	70	78/7/4^c^
10	2.0	1.2	EtN^i^Pr_2_	80	<10^d^
11^e^	2.5	1.2	Cl_2_‐Py	120	32/42/7
12	2.5	1.2	^t^Bu_2_‐Py	120	38/53/9

^a^Toluene (1.0 equiv.), heated without any additional solvent.

^b^Conversion determined by ^1^H NMR spectroscopy from the integration of functionalized toluene‐Me peaks after addition of C_6_D_6_ (ca. 0.4 mL) at the end of the reaction. In several examples the product was worked up to confirm the formation of **2a** and the relative isomer ratios.

^c^Reaction run for 24 h.

^d^Combined germylated isomers.

^e^Reaction run for 72 h.

From the optimisation study it also should be noted that increasing the amount of AlCl_3_ to >2 equivalents led to a different isomer ratio, with the *meta* isomer now dominating (entries 11 and 12). This was attributed to Lewis acid‐induced group migration (either of methyl or GeCl_3_) [36] as observed in other main group S_E_Ar reactions [25, 26]. The different C‐H germylation isomer distribution when using ≤2 equiv. versus 2.5 equiv. AlCl_3_ indicates germylation is proceeding irreversibly with ≤2 equiv. AlCl_3_, thus is under kinetic control (*vide infra*) under those conditions.

**FIGURE 2:**
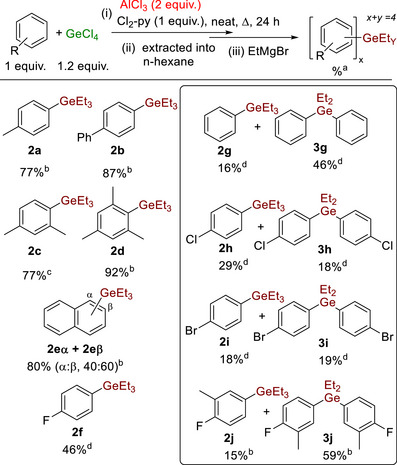
Substrate scope for germylation using 2 equiv. AlCl_3_. ^a^Isolated yields relative to starting arene. ^b^Run at 70°C. ^c^86:14 ratio of 1,2,4:1.3.5‐isomer. ^d^Run at 120°C/125°C. For the mono‐haloarenes the *para* isomer is the major product in each case; see supporting information for exact isomer ratios.

A scope exploration under neat conditions was conducted next with the Aryl‐GeCl_3_ products converted to the respective ArGeEt_3_ compound to enable isolation and characterisation. Initially, scoping studies focused on heterocycles amenable to other main group electrophile‐based sp^2^C‐H metalations [25, 26, 37]. However, under these germylation conditions, 2‐Me‐thiophene, benzothiophene and *N*‐Me‐carbazole all resulted in no observable aryl‐germane formation. Instead, they produced intractable mixtures along with black solids. As similar outcomes were observed using ^t^Bu_2_‐Py in place of Cl_2_‐Py this is attributed to AlCl_3_‐initiated oligomerisation/carbonisation [38, 39, 40]. Subsequent work focused on aromatics more nucleophilic than toluene that were found previously to be stable to electrophilic borylation using excess AlCl_3_ [30]. This revealed that biphenyl can be germylated selectively in the *para* position, with the GeEt_3_ derivative (**2b**, Figure 2) isolated in good yield (87%). *Meta*‐xylene produced the 1,2,4‐isomer, **2c**, post work‐up as the major product along with the 1,3,5‐isomer as the minor product. Formation of the former indicated that germylation *ortho* to a methyl group was feasible, an observation confirmed by the sp^2^C‐H germylation of mesitylene leading to **2d** in 92% yield. Note, for these methylated‐aryl substrates, no sp^3^C‐H germylation was observed, disfavouring deprotonative or radical‐based germylation processes [41].

The electrophilic germylation of naphthalene (Mayr *N* value = −3.9) [32] at 70°C produced an isomeric mixture of **2e** (α:β = 40:60) in good yield on work‐up (80%). The α position in naphthalene is the kinetically preferred site for S_E_Ar, while the β position is thermodynamically favored [25, 26]; thus, C–H germylation of this substrate is also irreversible under these conditions. Attempts to extend this germylation process to anthracene and phenanthrene failed, with the formation of insoluble black solids observed again, consistent with AlCl_3_‐induced carbonisation [38, 39, 40].

**SCHEME 2:**
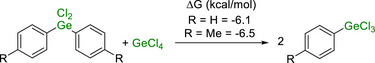
Gibbs free energy (Δ*G*) change at 25°C for forming the mono‐aryl germanes from Ar_2_GeCl_2_ and GeCl_4_.

Mono‐cyclic arenes less nucleophilic than toluene were explored next. The highly deactivated arene, 1,2‐dichlorobenzene, did not undergo germylation even under forcing conditions. In contrast, fluorobenzene underwent significant germylation on heating. After 24 h at 120°C, **2f** was isolated post‐workup in 46% yield (Figure 2). The need for a higher temperature (than toluene germylation) is consistent with the lower nucleophilicity of fluorobenzene (Mayr *N* value = −5.7) [32]. The even less nucleophilic arenes, benzene (*N* = −6.3), chlorobenzene (*N* = −7.0), and bromobenzene (*N* = −7.2) also all required heating to >70°C for significant C‐H germylation. However, for all three, alongside the ArGeEt_3_ products **2g**, **2h,** and **2i**, significant amounts of the diarylgermanes, **3g**, **3h**, **3i**, were isolated despite using 1.2 equiv. of GeCl_4_ (Figure 2, inset). A similar outcome was observed during the germylation of *o*‐fluorotoluene, producing a mixture of **2j** and **3j**. Calculations at the B3PW91(D3(BJ), Def2‐TZVPP, PhCl(SMD))//PBE0(D3(BJ), Def2‐SVP) level (all calculations discussed herein are performed at this level) revealed that the thermodynamic product is ArGeCl_3_ for both benzene and toluene (Scheme 2). The disparity in outcome between benzene and toluene germylation (for toluene, formation of **2a** dominates) therefore is kinetic in origin and indicates the germylation of benzene is irreversible even at 120°C with 2 equiv. AlCl_3_.

**SCHEME 3:**
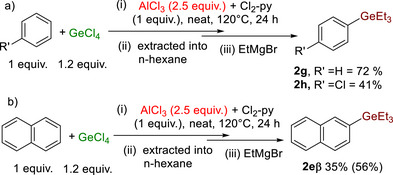
(a) More selective formation of mono‐aryl‐germanes using 2.5 equiv. AlCl_3_. Isolated yields post‐chromatography. (b) More selective formation of **2eβ** formed as a 6:1 ratio of **2eβ:2eα**. Yield in parenthesis determined versus internal standard.

Given the reaction free energies shown in Scheme 2, identifying conditions where germylation is reversible would enable formation of the ArGeCl_3_ derivatives more selectively. As the germylation of toluene using 2.5 equiv. of AlCl_3_ led to different isomer ratios relative to using 2 equiv. of AlCl_3_ (Table 1) it was hypothesised that 2.5 equiv. of AlCl_3_ would enable reversible germylation of benzene under otherwise identical conditions. Indeed, performing the germylation of benzene using 2.5 equiv. of AlCl_3_ led to the formation of the mono‐arylated product as the major species, with **2** **g** isolated in good yield (72%, Scheme 3a). The same approach also worked with chlorobenzene, with **2** **h** formed in 41% yield after 24 h. However, attempts to extend this approach to bromobenzene led to complex mixtures (attributed to AlCl_3_‐induced halide scrambling) [42]. Nevertheless, this approach also can be used to improve the selectivity in the germylation of naphthalene (producing a ca. 6:1 β:α product mixture, Scheme 3b).

**SCHEME 4:**
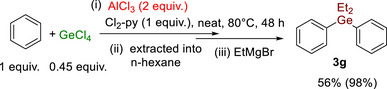
More selective formation of **3** **g** using 0.45 equiv. GeCl_4_. Yield = isolated, yield in parenthesis is vs an internal standard (the latter is as a 9:1 ratio **3g:2g**).

Varying the amount of GeCl_4_ in these electrophilic germylation reactions was then explored, targeting a more selective synthesis of Ph_2_GeCl_2_. Reducing the equivalents of GeCl_4_ used to 0.45 equiv. led to a ratio of 9:1 **3g**:**2g**, a significant improvement over conditions using 1.2 equiv. GeCl_4_ (which gave a 3:1 **3g:2g**). Formation of **3g/2g** was effectively quantitative (98% vs. internal standard), and **3g** could be isolated in 56% yield (Scheme 4).

**SCHEME 5:**
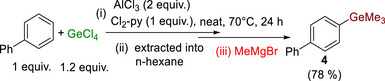
Formation of **4** by electrophilic germylation (isolated yield shown).

Finally, in the scoping, to demonstrate the versatility of the primary ArGeCl_3_ products, one was converted to a different aryl‐germane derivative. Compound **4** was formed by using a different Grignard reagent in the work‐up (Scheme 5). Note, ArGeMe_3_ are also desirable organogermane nucleophiles for use in functional group transformations [43].

While the substrate scope is consistent with an S_E_Ar‐type process, mechanistic insight was sought from deuterium labeling experiments. As the reactions under neat conditions contained varying amounts of solid, conditions in which a homogenous solution persisted throughout were identified. Thus, the electrophilic germylation reactions were performed in parallel in C_6_H_5_Cl and in C_6_D_5_Cl at 125°C. From this, a k_H_/k_D_ of 1.0 was found. Note, similar k_H_/k_D_ values have been reported in the nitration of chlorobenzene, and these values are consistent with arenium cation formation being rate‐limiting [36, 44].

## 3 Computational Studies

**SCHEME 6:**
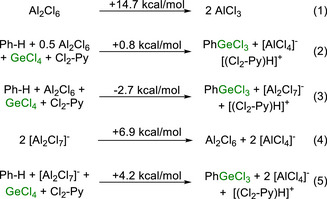
Gibbs free energy change (kcal/mol) at 125°C for various germylation reactions (Equations 2, 3, and 5) and for the interconversion of several aluminium Lewis acids (Equations 1 and 4). Method: B3PW91‐D3(BJ)/def2‐TZVPP, PhCl (SMD)//PBE0‐D3(BJ)/def2‐SVP.

Firstly, an explanation for the requirement for two equivalents of AlCl_3_ in this electrophilic germylation was sought. This stoichiometry was surprising initially, as a range of other electrophilic C‐H functionalisations only require one equivalent of AlCl_3_ as an activator to achieve a high yield [25, 26]. As the cleavage of Al_2_Cl_6_ into two equivalents of AlCl_3_ is significantly endergonic at 125°C (Scheme 6, Equation 1), all calculations herein use Al_2_Cl_6_ as using the correct speciation of “aluminium trichloride” is crucial to obtain meaningful energetics.

Electrophilic germylation using only 0.5 equivalent of Al_2_Cl_6_ would lead to the anionic by‐product being [AlCl_4_]^–^ (Equation 2). Therefore, the Gibbs free energy change was computed for the germylation of benzene producing [AlCl_4_]^−^ and for that producing [Al_2_Cl_7_]^−^ (Scheme 6, Equations 2 and 3). This revealed that while the C–H germylation of benzene producing [AlCl_4_]^–^ as by‐product is effectively thermoneutral at 125°C, it is 3.5 kcal/mol less favored than benzene germylation producing [Al_2_Cl_7_]^–^. This difference is presumably due to the higher chloride affinity of Al_2_Cl_6_ relative to AlCl_3_ [45]. However, the [AlCl_4_]^−^ formed from the germylation in eq. 2 will act as a Lewis base toward unreacted Al_2_Cl_6_ producing [Al_2_Cl_7_]^−^. Thus, when using 0.5 equiv. Al_2_Cl_6_ after 50% germylation has occurred all remaining Al_2_Cl_6_ will have been converted to [Al_2_Cl_7_]^–^. The production of Al_2_Cl_6_ from [Al_2_Cl_7_]^−^ adds an additional endergonic step (Scheme 6, Equation 4). When this additional step is included, benzene germylation becomes endergonic (Scheme 6, Equation 5), consistent with the observed stoichiometry required for good (>50%) germylation conversions.

**FIGURE 3:**
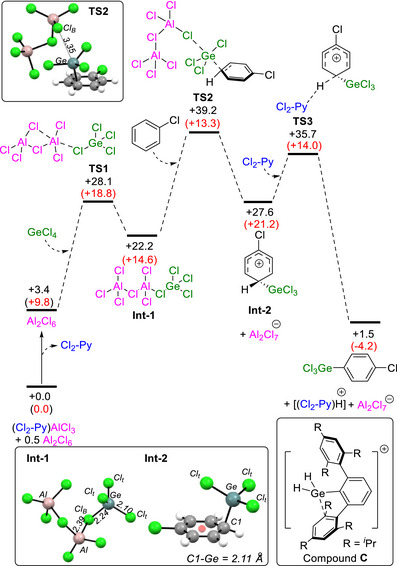
Computed Gibbs free energy (ΔH in parentheses in red) reaction profile (kcal/mol) at 125°C for the C–H germylation of chlorobenzene. Method: B3PW91(D3(BJ), Def2‐TZVPP, PhCl(SMD))//PBE0(D3(BJ), Def2‐SVP. Top inset: the structure of **TS2**. Bottom left inset: structures of **Int‐1** and **Int‐2** with select metrics (Å). Red sphere = centroid of the phenyl ring. Bottom right inset: intramolecular [Y_3_Ge(arene)]^+^ complex (compound **C**).

Next, the mechanism was investigated computationally for the germylation of chlorobenzene using GeCl_4_/Al_2_Cl_6_/Cl_2_‐Py at 125°C (this temperature is used to align with the experimental KIE measurements). While both AlCl_3_ and Al_2_Cl_6_ were investigated as the Lewis acid that is activating GeCl_4_, only Al_2_Cl_6_ led to identification of a plausible reaction mechanism [46]. A range of starting Lewis adducts were explored, with the lowest energy combination being the Lewis adduct (Cl_2_‐Py)AlCl_3_ and 0.5 eq. Al_2_Cl_6_ (see Figure S54), and this combination was taken as the starting point for this process. The calculated mechanism (depicted in Figure 3) proceeds through an S_E_Ar‐type process with formation of the arenium cation **Int‐2** involving the highest energy transition state (**TS2**). The calculated k_H_/k_D_ for **TS2** of 1.0 at 125°C is in excellent agreement with the experimentally determined k_H_/k_D_. Note, several stationary points in this calculated mechanism involve several components; in particular, rate‐limiting **TS2** corresponds to an overall barrier of 39.8 kcal/mol, which is rather too high for a reaction proceeding at 125°C. In addition, the overall reaction also was calculated to be slightly endergonic (by +1.5 kcal/mol). This is attributed to the entropic contribution for these associative processes, which when calculated in the gas‐phase is accepted to be over‐estimated compared to the solution phase [47, 48, 49]. Thus, the absolute barrier computed for this pathway is likely to be exaggerated. Figure 3 also provides (in red) the computed enthalpies at 125°C, which emphasize the significant entropic contribution (also see Figure S55 for further discussion).

Analyzing the profile in more detail revealed that as germylation progresses toward **TS1,** there is significant elongation of one of the bridging Al‐(μ‐Cl)‐Al bonds (from 2.28 Å in Al_2_Cl_6_ to 2.72 Å in **TS1**) concomitant with the formation of a Ge‐(μ‐Cl)‐Al unit. As previously reported, cleaving one of the bridging Al‐(μ‐Cl)‐Al bonds in Al_2_Cl_6_ generates a highly electrophilic aluminium centre [30]. **Int‐1** is then formed, and in **Int‐1** the sum of the Cl_t_‐Ge‐Cl_t_ angles, at 337.6° (bottom Figure 3), is closer to that expected for a tetrahedral germanium centre (327°) than for a [GeCl_3_]^+^ species (360°). Thus, **Int‐1** has only partial germylium cation character [14, 50], which is consistent with the short Ge‐Cl_B_ distance of 2.24 Å. Nevertheless, the transfer of {GeCl_3_}^+^ to the arene proceeds in an associative manner through **TS2** and leads to the arenium cation **Int‐2** and [Al_2_Cl_7_]^–^. **TS2** (inset top Figure 3) is a late transition state and contains a significantly elongated Ge···Cl_B_ distance (3.35 Å) and a short Ge‐C distance (2.18 Å) comparable to that in **Int‐2** (2.11 Å). **Int‐2** (Figure 3, bottom) lies on the σ···π arenium complex continuum [51], with an Aryl_centroid_‐C1‐Ge angle of 107.8°. While [Y_3_Ge(arene)]^+^ cations have been proposed in a number of catalytic cycles [52], to our knowledge there are no structurally characterised examples. One solid‐state structure relevant to this work is the intramolecular arenium complex, compound **C** (inset, bottom Figure 3) [53]. Compound **C** contains a [Y_3_Ge(arene)]^+^ sub‐unit with a Ge—C_arene_ distance (2.262(3) Å) that is notably longer than the comparable interaction in **Int‐2**. This indicates that **Int‐2** will have a significantly lowered pK_a_ value (relative to that of free chlorobenzene), which will facilitate deprotonation by Cl_2_‐Py.

The reaction profile in Figure 3 uses chlorobenzene as solvent, but most reactions herein were performed neat. The neat reactions contain various compounds that are liquid at the reaction temperatures used. Furthermore, as the reaction proceeds [(Cl_2_‐Py)H][Al_2_Cl_7_] is produced and closely related [pyridinium][Al_2_Cl_7_] salts are ionic liquids [54, 55]. As ionic liquids have a significantly higher dielectric constant [56], ε, than chlorobenzene (ε PhCl = 5.62) an analysis of how transition state energies vary as the solvent dielectric changes was performed (see Table S3). As ε increases the energy of **TS1** remains effectively constant but both **TS2** and **TS3** drop in energy. The decrease in the energy of **TS2** and **TS3** with increasing ε, combined with the higher reagent concentration of the neat reactions is consistent with high yielding germylation occurring at lower temperatures under neat conditions relative to that in chlorobenzene.

## 4 Conclusion

High‐yielding intermolecular electrophilic germylation of arenes has been achieved for the first time. This process uses simple and inexpensive reagents (GeCl_4_, AlCl_3_ and 2,6‐dichloropyridine) to produce ArGeCl_3_ species that can be readily converted into desirable ArGe(alkyl)_3_ compounds. The identification of 2,6‐dichloropyridine as an efficient base was key to promoting electrophilic aromatic substitution using these main group halides. By understanding the thermodynamics of this transformation and how to enable reversible germylation, the reaction conditions were modified to selectively synthesise either the Ar_2_GeCl_2_ or the ArGeCl_3_ derivatives. Mechanistic and computational analyses were consistent with an S_E_Ar‐type process where Al_2_Cl_6_ is the Lewis acid that generates a germanium electrophile able to effect germylation in combination with an appropriate base. Overall, this work provides a new route to increasingly popular organogermane reagents, while demonstrating that a high yielding intermolecular Germa–Friedel–Crafts reaction is possible provided an appropriate Brønsted base is used.

## Conflicts of Interest

The authors declare no conflicts of interest.

## Supporting information




**Supporting File 1**: anie72051‐sup‐0001‐SuppMat.pdf.

**Supporting File 2**: anie72051‐sup‐0002‐Data.zip.

## Data Availability

The data that support the findings of this study are available from the corresponding author upon reasonable request.
