# Supplementary material for: Intermolecular Electrophilic Germylation Using GeCl4 and Al2Cl6
Source: Angew Chem Int Ed Engl. 2026 Apr 13;65(22):e7070836. doi: 10.1002/anie.7070836 (PMC13206196; doi:10.1002/anie.7070836)
Supplement: Supplementary file 1 — Supporting File 1: anie72051‐sup‐0001‐SuppMat.pdf. [file ANIE-65-e7070836-s002.pdf]

Supporting Information for:

Intermolecular Electrophilic Germylation using $GeCl_4$ and Al_2Cl_6

Justyna Łosiewicz[a‡], Milan Kumar Bisai[a‡], Zachary A. Bub[a], Stuart A. Macgregor[b]* and Michael J. Ingleson[a]*

[a] EaStCHEM School of Chemistry, University of Edinburgh, Edinburgh, EH9 3FJ, UK.

[b] EaStCHEM School of Chemistry, North Haugh, University of St Andrews, St. Andrews, KY16 9ST, UK.

Contents

S1. General considerations....................S2

S2. Synthesis of aryl germane compounds....................S3

S3. Mechanistic investigation....................S41

S4. Computational details....................S48

S5. References....................S63

S1. General considerations

Unless otherwise mentioned, all the experiments were carried out under an inert atmosphere using either standard Schlenk techniques or in a MBraun glovebox (<0.1 ppm H_2O/O_2).

Chlorobenzene and C_6D_6 were distilled over CaH_2 and stored over activated 3 Å molecular sieves. C_6D_5Cl and $CDCl_3$ were dried over activated 3 Å molecular sieves. All other solvents were obtained from an Inert PureSolv MD5 solvent purification system (SPS) and further dried over activated 3 Å molecular sieves. All other chemicals were, unless otherwise stated, purchased from commercial sources and used as received. $GeCl_4$ was transferred to an ampule prior to use.

Column chromatography was performed on a Teledyne Isco CombiFlash® 100 instrument using Advion-Interchim columns (spherical silica, 25 μm).

^{1}H, $^{1}H\{^{19}F\}$, $^{13}C\{^{1}H\}$, ^{19}F, $^{19}F\{^{1}H\}$ and 2D NMR spectra were recorded on Bruker Avance Neo spectrometer equipped with a 5 mm helium-cooled BBO cryoprobe or Bruker Avance AVIII HD spectrometer equipped with a 5 mm nitrogen-cooled BBO Prodigy cryoprobe or Bruker Avance AVIII HD spectrometer equipped with a 5 mm helium-cooled H/N/C/F QCI cryoprobe and referenced to the solvent in use for ^{1}H, $^{1}H\{^{19}F\}$ and $^{13}C\{^{1}H\}$, while ^{19}F and $^{19}F\{^{1}H\}$ shifts were referenced relative to external C_6F_6. Chemical shifts are reported as dimensionless δ values in ppm, coupling constants J are given in Hertz (Hz). The multiplicity of the signals is indicated as “br. s”, “s”, “d”, “t” “q” “pent”, “sept” or “m” for broad singlet, singlet, doublet, triplet, quartet, pentet, septet or multiplet, respectively. Unless otherwise stated, NMR spectroscopy was undertaken at room temperature (~27°C). Quaternary carbon atoms directly bonded to -$GeCl_3$ were often not observed in the $^{13}C\{^{1}H\}$ NMR spectra. Data analysis was carried out using MestReNova.

Mass spectrometry was performed by the Scottish Instrumentation and Resource Centre for Advanced Mass Spectrometry (SIRCAMS) at the University of Edinburgh. Mass spectrometry was performed using the APCI source on the 12T FT-ICR Solarix (Bruker). Data analysis was carried out using Data Analysis (Bruker).

S2. Synthesis of aryl germane compounds

S2.1. General procedure 1: Optimisation of the $AlCl_3$ mediated C−H germylation of toluene.

In a glovebox, $AlCl_3$ (0.0-1.5 mmol, 0-3.0 equiv.) and base (0.5-0.6 mmol, 1.0-1.2 equiv.) were charged in a J. Young's NMR tube. Subsequently, toluene (0.5 mmol, 1.0 equiv.) and $GeCl_4$ (0.5-0.75 mmol, 1.0-1.5 equiv.) were added to the reaction mixture at room temperature. The NMR tube was then sealed under argon and heated at a specified temperature for a specified time. Upon completion, C_6D_6 (0.4 mL) was added to the reaction mixture and the conversion to the products was determined via NMR spectroscopy by the integration of diagnostic ^{1}H (*Me*-$C_6H_4GeCl_3$) resonances.

Table S1: Aluminium trichloride mediated C−H germylation of toluene.[a]

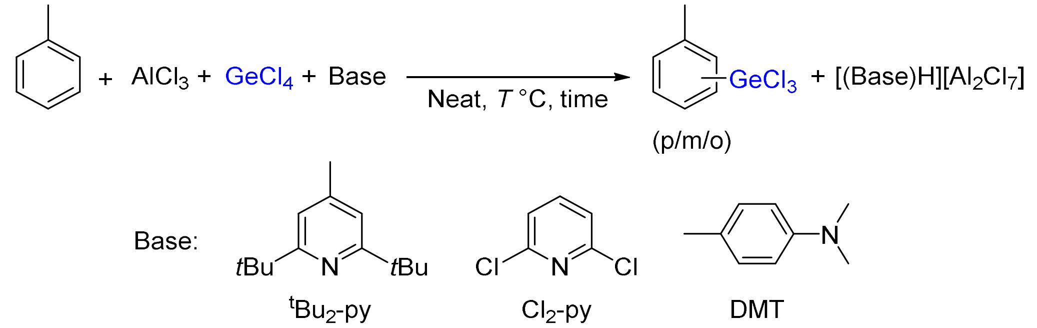

Entry	$AlCl_3$ (equiv.)	$GeCl_4$ (equiv.)	Base	Base (equiv.)	T (°C)	time (h)	Conversion p/m/o(%)[b]
**1**	-	1.2	$^{t}Bu_2$-py	1.0	120	16	0.0
**2**	2.0	1.2	-	-	120	16	0.0
**3**	2.0	1.2	$^{t}Bu_2$-py	1.0	120	16	58/23/12
**4**	2.0	1.2	$^{t}Bu_2$-py	1.2	120	16	37/15/7
**5**	2.0	1.0	$^{t}Bu_2$-py	1.2	120	16	30/13/5
**6**[c]	2.0	1.0	$^{t}Bu_2$-py	1.0	120	16	45/25/9
**7**	2.0	1.5	$^{t}Bu_2$-py	1.0	120	16	60/23/12
**8**	2.5	1.2	$^{t}Bu_2$-py	1.0	120	16	38/53/9
**9**	2.5	1.2	$^{t}Bu_2$-py	1.0	120	72	39/40/9
**10**	3.0	1.2	$^{t}Bu_2$-py	1.0	120	16	36/55/9
**11**	2.0	1.2	$^{t}Bu_2$-py	1.0	100	16	65/16/11
**12**	2.0	1.2	$^{t}Bu_2$-py	1.0	100	10	62/22/11
**13**	2.0	1.2	$^{t}Bu_2$-py	1.0	80	16	52/16/9
**14**	2.0	1.2	PPh_3	1.0	80	16	< 5% combined

**15**	2.0	1.2	C_5H_5N	1.0	80	16	< 5% combined
**16**	2.0	1.2	2,6-lutidine	1.0	80	16	47/22/7
**17**	2.0	1.2	EtN^iPr_2	1.0	100	16	<10 combined
**18**[c]	2.0	1.2	DMT	1.0	100	16	52/13/4
**19**	2.0	1.2	Cl_2-py	1.0	100	16	64/25/8
**20**	2.0	1.2	Cl_2-py	1.0	100	6	60/24/9
**21**	2.0	1.2	Cl_2-py	1.0	80	16	77/14/6
**22**	2.0	1.2	Cl_2-py	1.0	80	19	79/10/5
**23**	2.0	1.2	Cl_2-py	1.0	70	19	73/6/3
**24**	2.0	1.2	Cl_2-py	1.0	70	24	78/7/4
**25**	2.5	1.2	Cl_2-py	1.0	120	72	32/42/7

[a] Toluene (1.0 equiv.), $AlCl_3$ (2.0-3.0 equiv.), Base (1.0-1.2 equiv.), and $GeCl_4$ (1.0-1.5 equiv.) heated without using any additional solvent. [b] Conversion determined by the ^1H NMR spectroscopy from the integration of functionalized toluene -Me peaks after addition of C_6D_6 (0.4 mL) at the end. [c] Unknown side products also formed in minor amount.

S2.1.1. Optimisation example of the C–H germylation of toluene – para isomer major

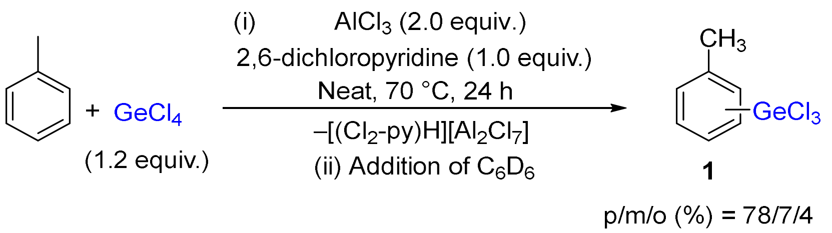

As per general procedure 1, $GeCl_3$-tolyl isomers (**1**) were prepared using toluene (54 µL, 0.5 mmol, 1.0 equiv.), $GeCl_4$ (70 µL, 0.6 mmol, 1.2 equiv.), $AlCl_3$ (134 mg, 1.0 mmol, 2.0 equiv.) and 2,6-dichloropyridine (74 mg, 0.5 mmol, 1.0 equiv.) and heating at 70 °C for 24 h. Upon completion, C_6D_6 (0.4 mL) was added to the reaction mixture and the conversion to the products was determined via NMR spectroscopy by the integration of diagnostic ^1H (*Me*-$C_6H_4GeCl_3$) resonances. The in-situ conversion to **1** vs unreacted toluene (1%) was determined (89%, *p:m:o* isomer = 78:7:4). The remaining 10% is tentatively assigned to $GeEt_2$-(p-tolyl)$_2$ (*vide infra*).

1, para-isomer:

^1H NMR (500 MHz, C_6D_6): δ 7.31 (d, J = 8.3 Hz, 2H, Ar*H*), 6.90 (d, J = 8.3 Hz, 2H, Ar*H*), 2.03 (s, 3H, C*H*$_3$).

$^{13}C\{^1H\}$ NMR (126 MHz, C_6D_6): δ 144.2, 131.2, 130.4, 21.5.

These data are in agreement with those reported previously in the literature.[1]

1, meta-isomer:

^{1}H NMR (500 MHz, C_6D_6): δ 7.26-23 (m, 1H, Ar*H*), 7.15-7.10 (m, 1H, Ar*H*), 7.04-6.98 (m, 2H, Ar*H*), 1.96 (s, 3H, CH_3).

1, ortho-isomer:

^{1}H NMR (500 MHz, C_6D_6): δ 7.52 (dd, J = 7.7, 1.5 Hz, 1H, Ar*H*), 6.98-6.94 (m, 1H, Ar*H*), 6.81-6.70 (m, 2H, Ar*H*), 2.35 (s, 3H, CH_3).

These data are in agreement with those reported previously in the literature.[2]

Due to meta- and ortho-isomers being the minor products, we were unable to assign $^{13}C\{^1H\}$ NMR data unambiguously for these isomers of **1**.

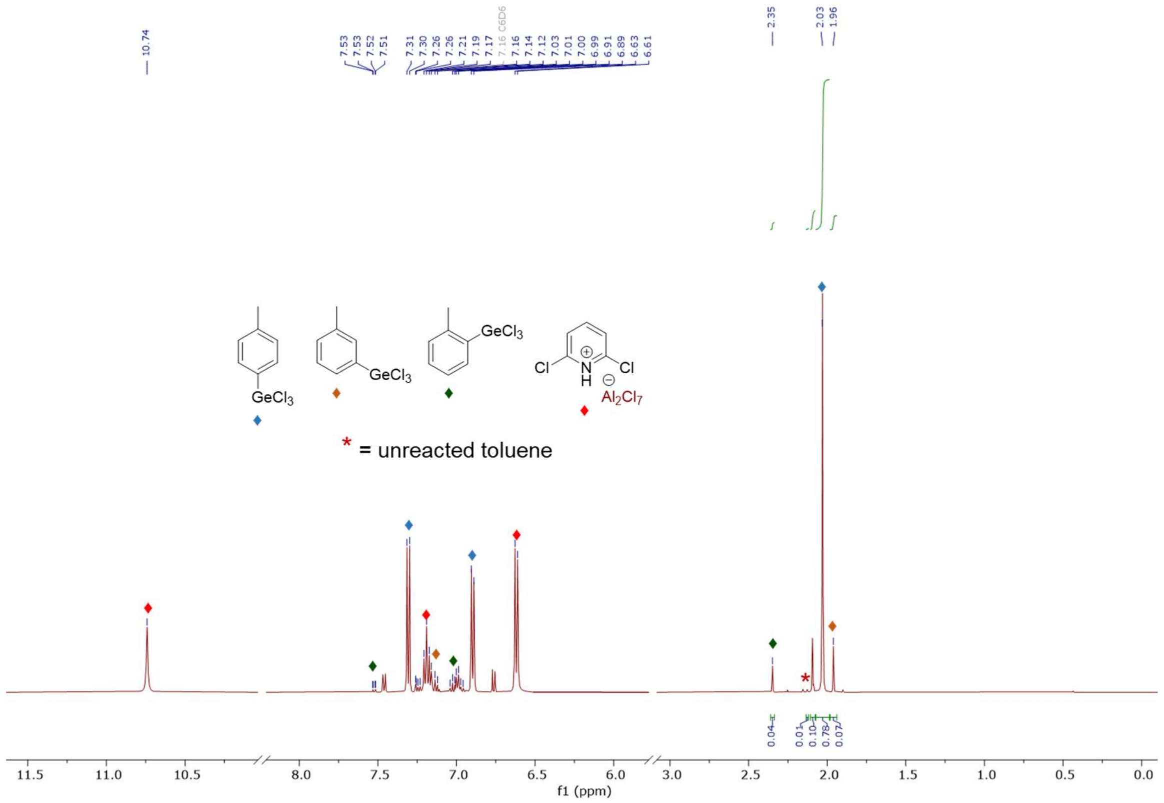

Figure S1: C–H germylation of toluene upon reaction completion after addition of C_6D_6 by in-situ ^{1}H NMR spectroscopy yielding predominantly the para isomer. The unmarked resonance (10%) in the -CH_3 region is tentatively assigned to $GeEt_2$-(p-tolyl)$_2$.

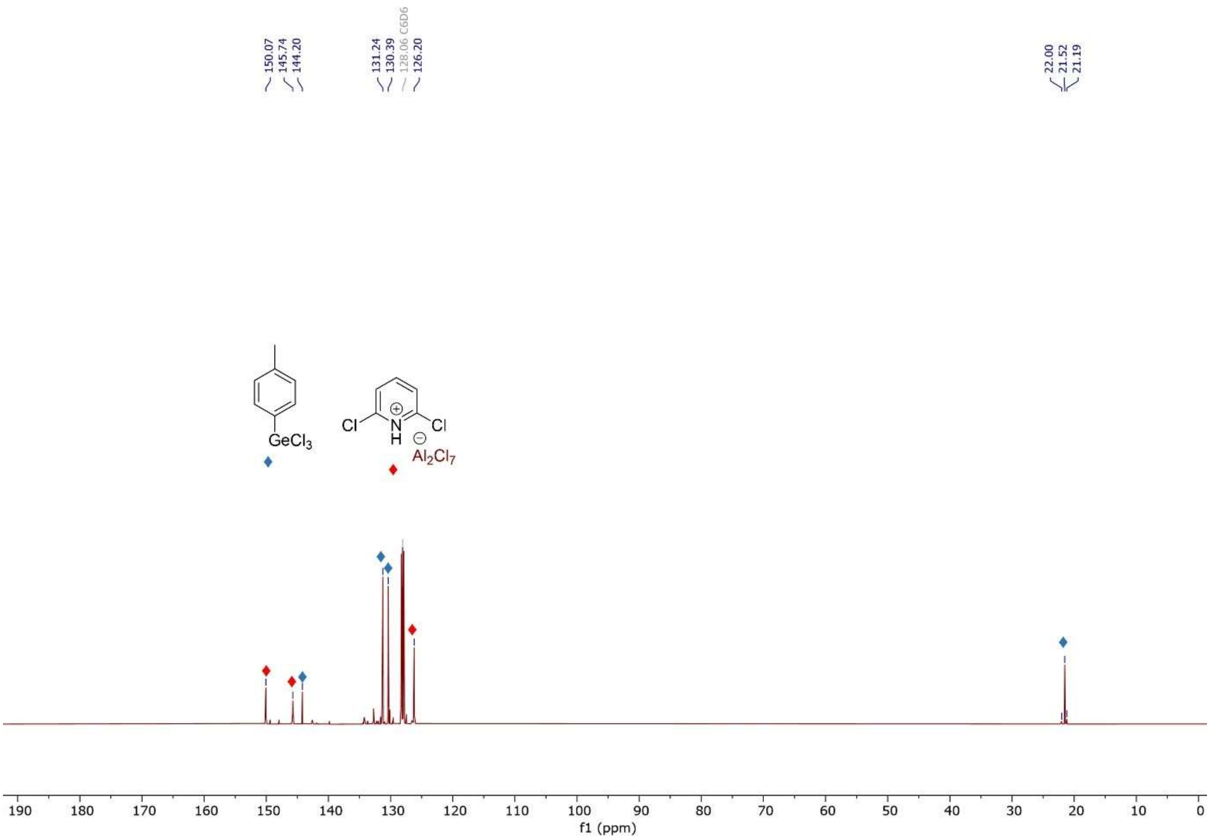

Figure S2: C–H germylation of toluene in C_6D_6 by in-situ $^{13}C\{^1H\}$ spectroscopy yielding predominantly the para isomer.

S2.1.2. Optimisation example of the C−H germylation of toluene – meta isomer major

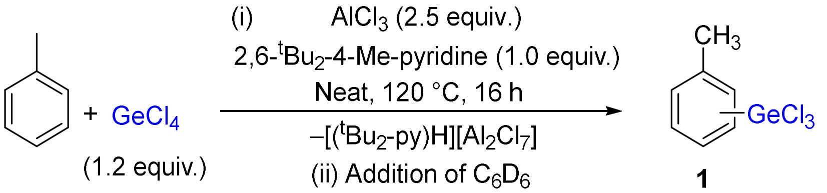

1

p/m/o (%) = 38/53/9

As per general procedure 1, $GeCl_3$-tolyl isomers (**1**) were prepared using toluene (54 µL, 0.5 mmol, 1.0 equiv.), $GeCl_4$ (70 µL, 0.6 mmol, 1.2 equiv.), $AlCl_3$ (167 mg, 1.25 mmol, 2.5 equiv.) and 2,6-ditertbutyl-4-methyl-pyridine (103 mg, 0.5 mmol, 1.0 equiv.) and heating at 120 °C for 416h. Upon completion, C_6D_6 (0.4 mL) was added to the reaction mixture and the conversion to the products was determined via NMR spectroscopy by the integration of diagnostic ^1H (*Me*-$C_6H_4GeCl_3$) resonances. The in-situ conversion to **1** vs unreacted toluene was determined (100%, *p:m:o* isomer = 38:53:9).

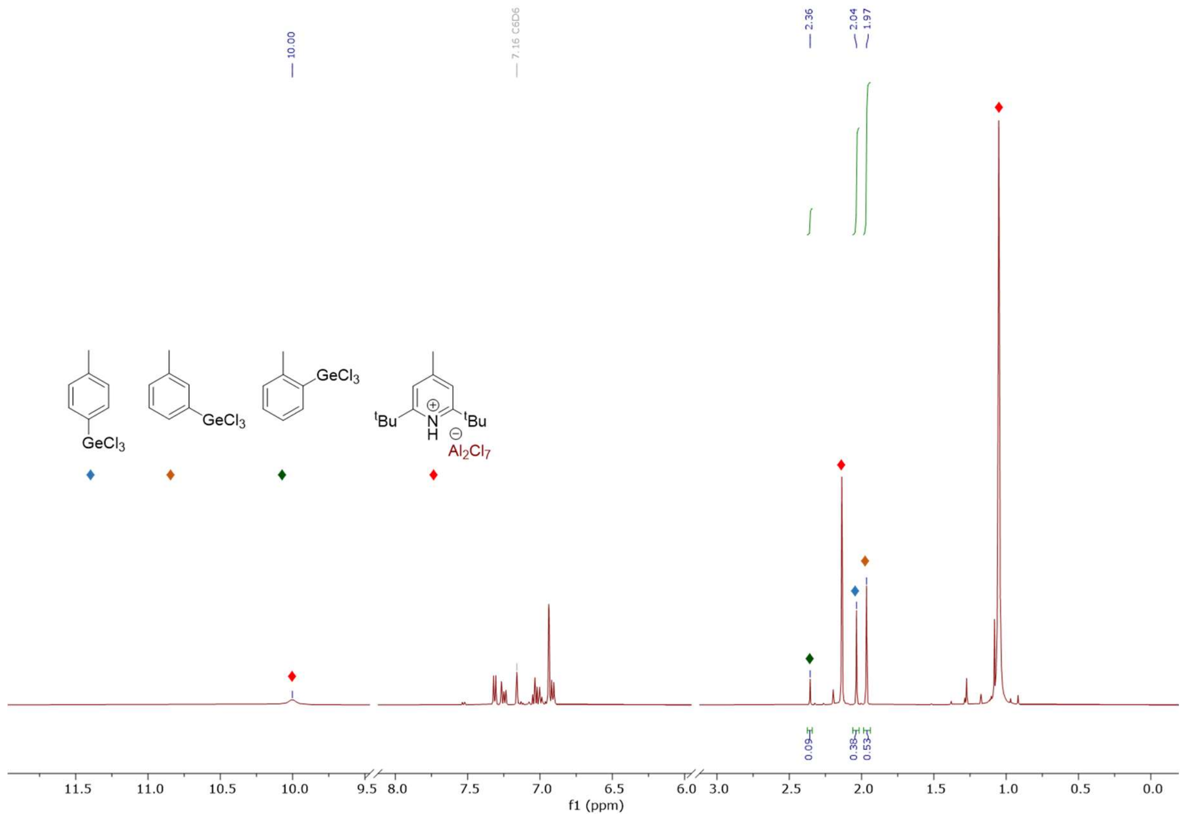

Figure S3: C–H germylation of toluene in C_6D_6 by in-situ ^1H NMR spectroscopy yielding predominantly the meta isomer.

S2.2. General procedure 2: Aluminium trichloride mediated C−H germylation of arenes.

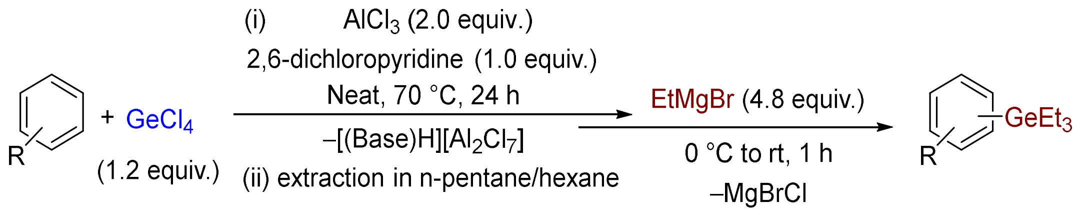

In a glovebox, $AlCl_3$ (134 mg, 1.0 mmol, 2.0 equiv.), and 2,6-dichloropyridine (74 mg, 0.5 mmol, 1.0 equiv.) were charged in a J. Young's ampule (5 mL). Subsequently, arene (0.5 mmol, 1.0 equiv.) and $GeCl_4$ (70 µL, 0.6 mmol, 1.2 equiv.) were added to the reaction mixture at room temperature. The ampule was then sealed under argon and heated at 70 °C for 24 h. Upon completion, n-pentane or n-hexane (5 mL) was added to the reaction and stirred for 5 min and then settled to obtain two separate immiscible liquid layers (the second layer is attributed to a [Cl_2-pyH][Al_2Cl_7] rich phase which is insoluble in alkane). The homogenous alkane layer was then separated and the remaining "[Cl_2-pyH][Al_2Cl_7] layer" was subsequently washed with n-pentane or n-hexane (2x5 mL). Combined the solution of the Arene$GeCl_3$ was cooled to 0 °C and EtMgBr (3.0 M solution in E_2O, 0.8 mL, 2.4 mmol, 4.8 equiv. w.r.t. arene) was added, which led to immediate formation of a white precipitate. The reaction was then warmed to room temperature and stirred for 1 h. Upon completion, the reaction was slowly quenched with 10 mL water at room temperature and the organic layer was extracted with dichloromethane (4x15 mL). The combined organic layer was washed with brine (10 mL), dried over $MgSO_4$, filtered, and concentrated *in vacuo.* When stated, CH_2Br_2 (35 µL, 0.5 mmol, 1 eq.) was added as an internal standard to determine the crude NMR yield. When stated, the crude product was then subjected to column chromatography on silica gel using 40/60 petroleum ether (100%) to elute the products.

S2.2.1. C–H germylation of toluene

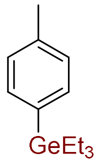

2a

As per general procedure 2, p-$GeEt_3$-tolyl (**2a**) was prepared using toluene (54 µL, 0.5 mmol, 1.0 equiv.) followed by extraction in n-hexane. **2a** was purified by column chromatography on silica gel using 40/60 petroleum ether (100%) eluent and isolated as a colourless oil. Yield: 77% (97 mg).

^{1}H NMR (500 MHz, $CDCl_3$): δ 7.36 (d, J = 8.1 Hz, 2H, Ar*H*), 7.19 (d, J = 8.1 Hz, 2H, Ar*H*), 2.37 (s, 3H, CH_3), 1.10-1.07 (m, 9H, GeEt_3), 1.02-0.97 (m, 6H, GeEt_3).

^{13}C{^{1}H} NMR (126 MHz, $CDCl_3$): δ 137.8, 136.0, 133.9, 128.7, 21.4, 8.9, 4.2.

Mass Spectrometry: Calculated [$(M\text{-}Et)^+$] = 223.05420 ($C_{11}H_{17}Ge$), Observed [$(M\text{-}Et)^+$] = 223.05375.

These data are in agreement with those reported previously in the literature.[3]

Note: A minor compound tentatively assigned to $GeEt_2$-(p-tolyl)$_2$ based on ^{1}H NMR spectroscopy was also observed via mass spectrometry.

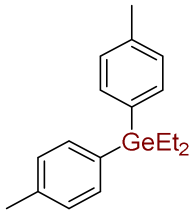

Mass Spectrometry: Calculated [$(M\text{-}Et)^+$] = 285.06985 ($C_{16}H_{19}Ge$), Observed [$(M\text{-}Et)^+$] = 285.06948.

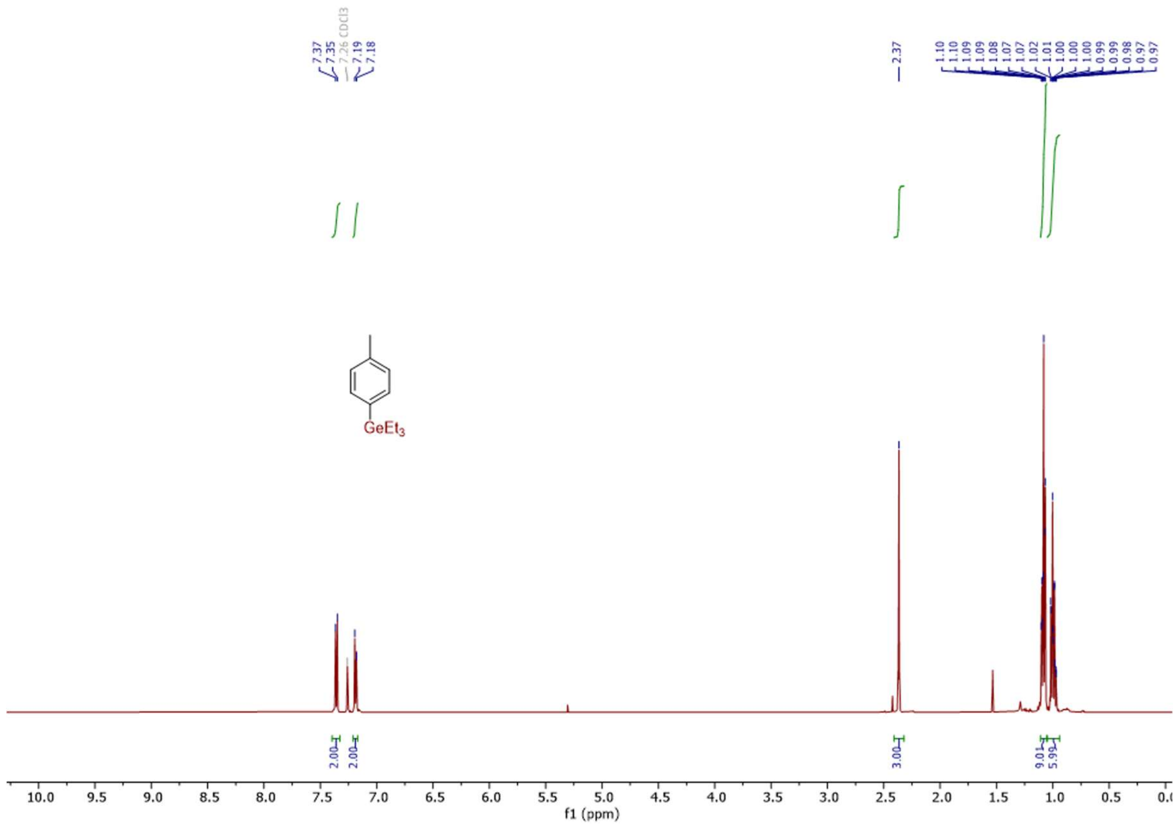

Figure S4: ^{1}H NMR spectrum of compound **2a** in $CDCl_3$.

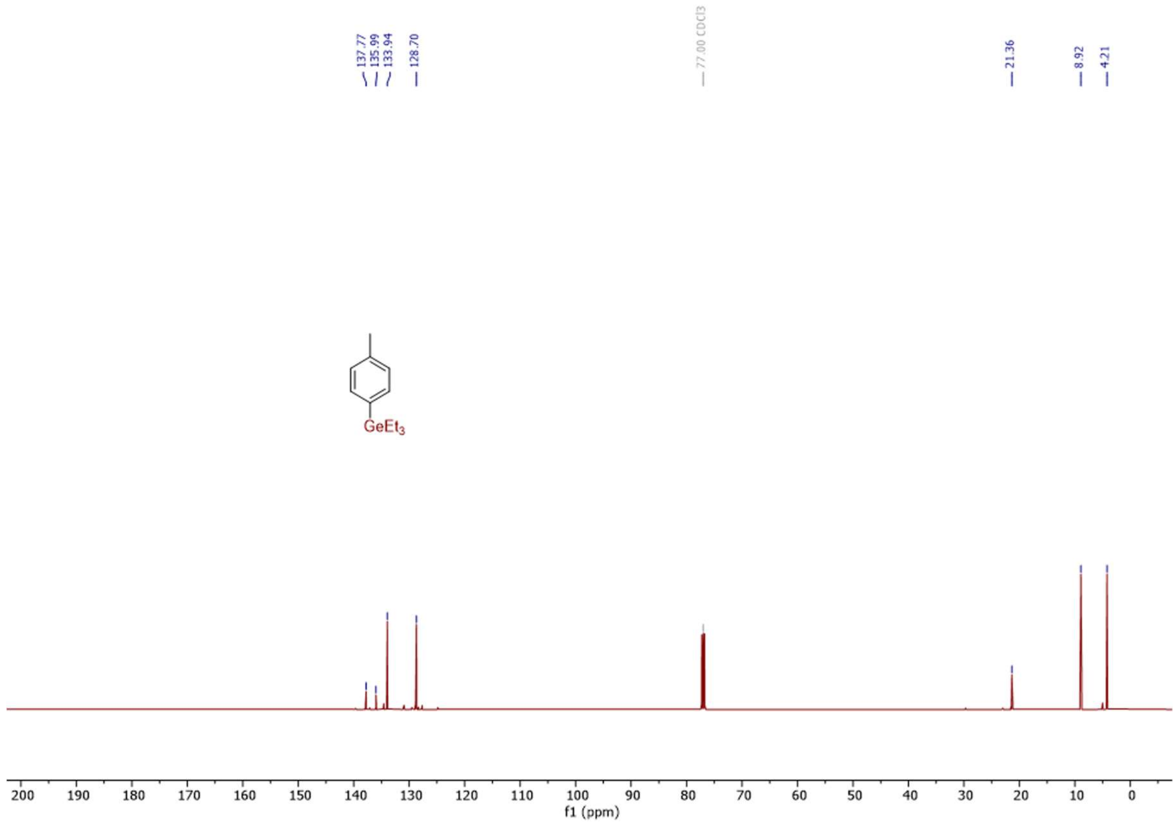

Figure S5: $^{13}C\{^{1}H\}$ NMR spectrum of compound **2a** in $CDCl_3$.

S2.2.2. C–H germylation of biphenyl

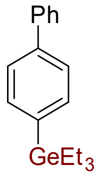

2b

As per general procedure 2, p-GeEt$_3$-biphenyl (**2b**) was prepared using biphenyl (80 mg, 0.5 mmol, 1.0 equiv.) followed by extraction in n-hexane. **2b** was purified by column chromatography on silica gel using 40/60 petroleum ether (100%) eluent and isolated as a colourless oil. Yield: 87% (136 mg).

^{1}H NMR (600 MHz, CDCl$_3$): δ 7.62-7.56 (m, 4H, Ar*H*), 7.54-7.49 (m, 2H, Ar*H*), 7.47-7.41 (m, 2H, Ar*H*), 7.38-7.31 (m, 1H, Ar*H*), 1.13-1.06 (m, 9H, Ge*Et*$_3$), 1.06-0.99 (m, 6H, Ge*Et*$_3$).

^{13}C{^{1}H} NMR (151 MHz, CDCl$_3$): δ 141.4, 141.1 138.9, 134.6, 128.9, 127.3, 127.3, 126.7, 9.1, 4.4.

Mass Spectrometry: Calculated [(M-Et)$^+$] = 285.06959, Observed [(M-Et)$^+$] = 285.06899.

These data are in agreement with those reported previously in the literature.[4]

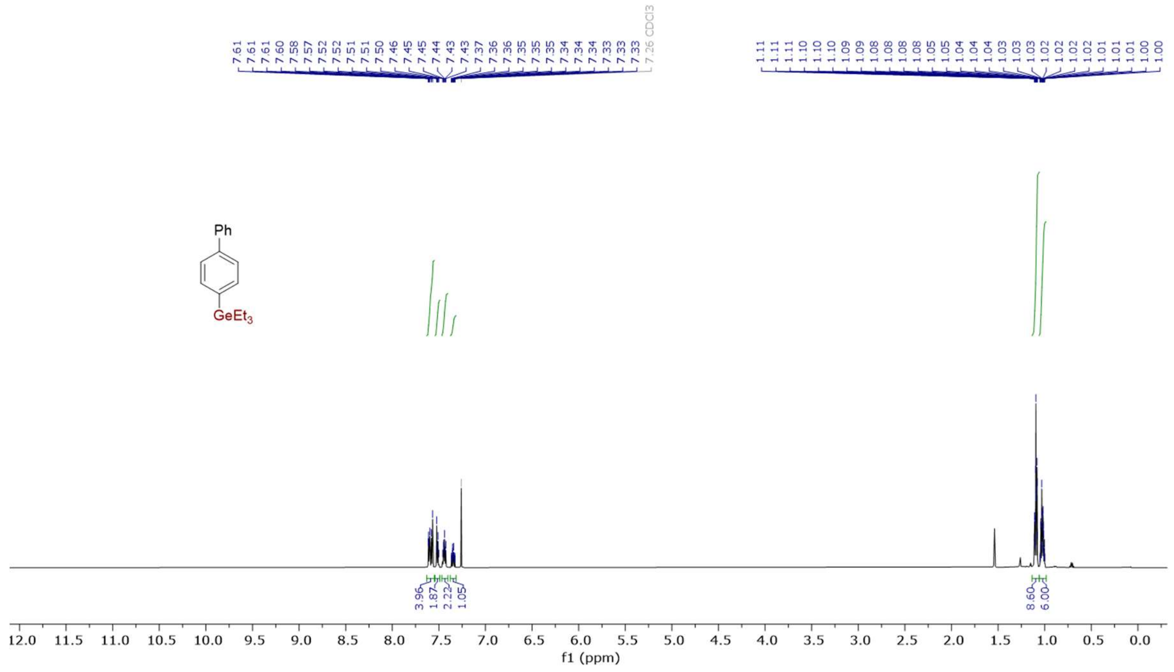

Figure S6: ^{1}H NMR spectrum of compound **2b** in CDCl$_3$.

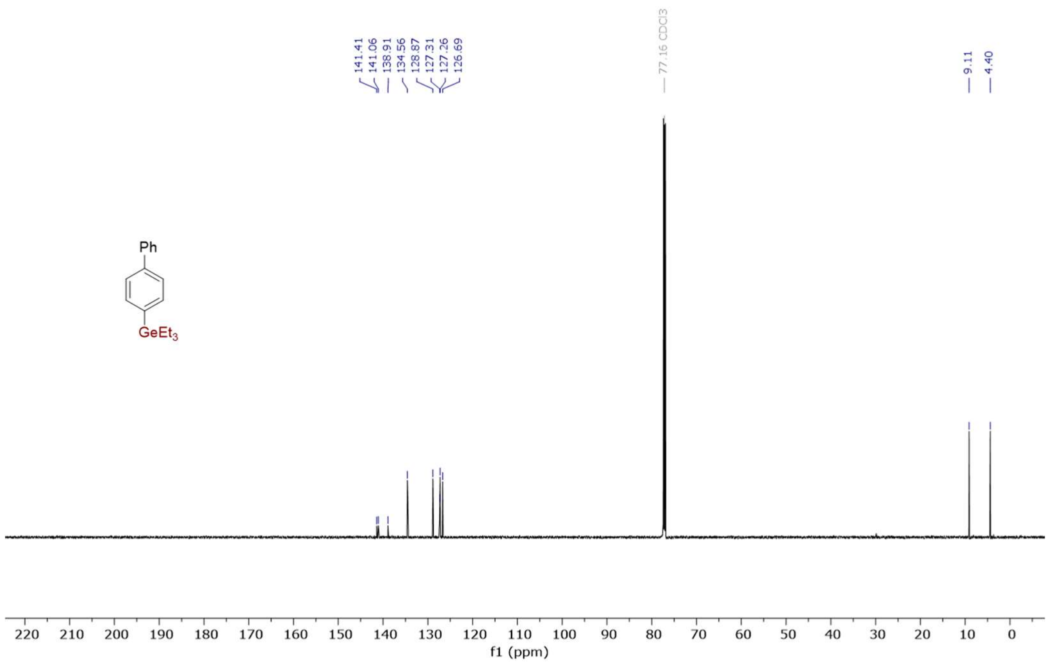

Figure S7: $^{13}C\{^1H\}$ NMR spectrum of compound **2b** in $CDCl_3$.

Synthesis of Compound 4:

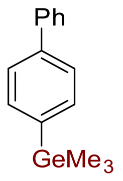

4

As per modified general procedure 2, p-$GeMe_3$-biphenyl (**4**) was prepared using biphenyl (80 mg, 0.5 mmol, 1.0 equiv.) followed by extraction in n-hexane. The solution of the biphenyl-$GeCl_3$ was dried in-vacuo and redissolved in Et_2O (15 mL), cooled to 0 °C and MeMgBr added (3.0 M solution in E_2O, 0.8 mL, 2.4 mmol, 4.8 equiv. w.r.t. arene). **4** was isolated as a colourless oil without further purification. Yield: 78% (105 mg).

^{1}H NMR (500 MHz, $CDCl_3$): δ 7.70-7.65 (m, 6H, Ar*H*), 7.54-7.51 (m, 2H, Ar*H*), 7.45-7.42 (m, 1H, Ar*H*), 0.53 (s, 9H, Ge*Me*$_3$).

$^{13}C\{^1H\}$ NMR (126 MHz, $CDCl_3$): δ 141.5, 141.4, 141.3, 133.6, 128.9, 127.4, 127.3, 126.8, -1.6.

These data are in agreement with those reported previously in the literature.[4]

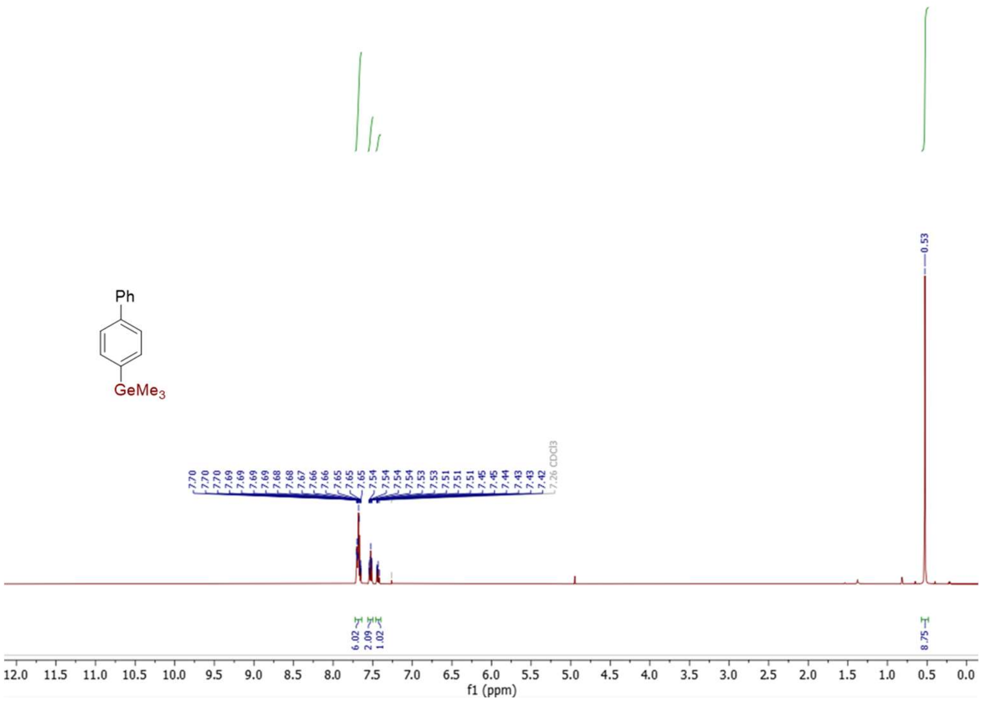

Figure S8: ^{1}H NMR spectrum of compound **4** in $CDCl_3$.

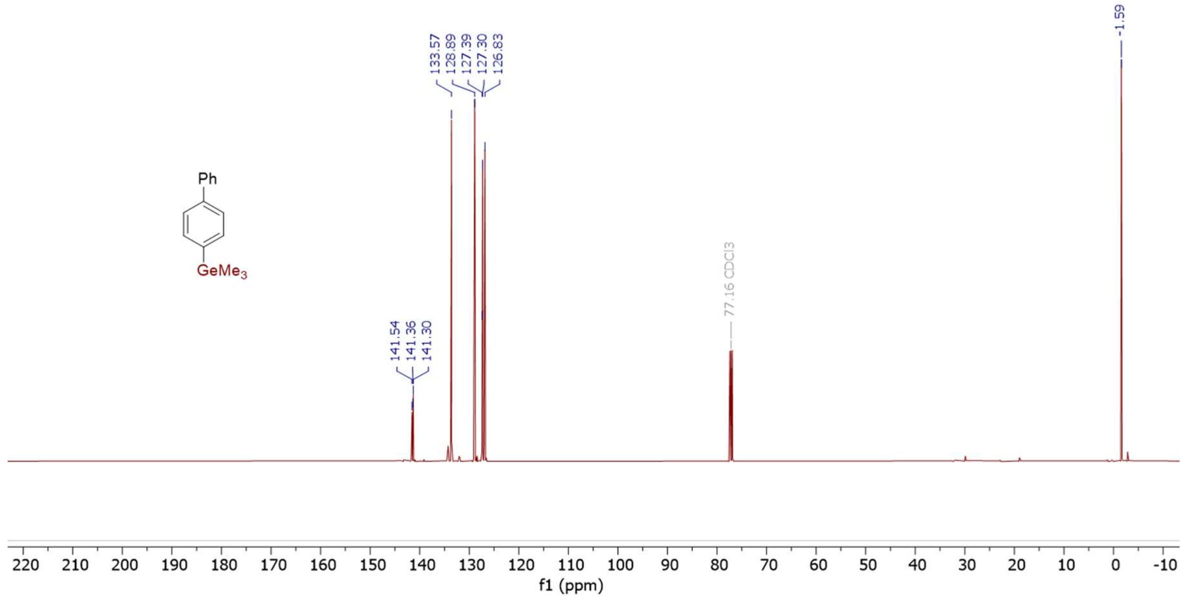

Figure S9: $^{13}C\{^{1}H\}$ NMR spectrum of compound **4** in $CDCl_3$.

S2.2.3. C–H germylation of m-xylene

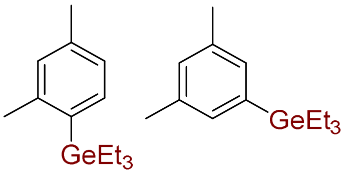

2c (major) **2c' (minor)**

6 : 1 ratio

As per general procedure 2, o-$GeEt_3$-m-xylene (**2c**) was prepared using m-xylene (62 µL, 0.5 mmol, 1.0 equiv.) followed by extraction in n-pentane. CH_2Br_2 (35 µL, 0.5 mmol, 1 eq.) was added as an internal standard to determine the crude NMR yield. **2c** was isolated together with a minor product m-$GeEt_3$-m-xylene (**2c'**) in a 6:1 ratio (vs internal standard) as a colourless oil without further purification. Combined yield: 77% (100 mg).

2c

^{1}H NMR (600 MHz, $CDCl_3$): δ 7.34 (d, J = 7.5 Hz, 1H, Ar*H*), 7.09 (d, J = 2.0 Hz, 1H, Ar*H*), 7.06 (dd, J = 7.6, 1.8 Hz, 1H, Ar*H*), 2.46 (s, 3H, CH_3), 2.39 (s, 3H, CH_3), 1.20-1.08 (m, 15H, GeEt_3, overlapping with **2c'**).

^{13}C{^{1}H} NMR (151 MHz, $CDCl_3$): δ 143.1, 137.9, 134.6, 134.2, 130.5, 125.6, 22.8, 21.1, 9.0, 5.0.

These data are in agreement with those reported previously in the literature.[4]

2c'

^{1}H NMR (600 MHz, $CDCl_3$): δ 7.14 (d, J = 1.8 Hz, 2H, Ar*H*), 7.05-7.04 (m, 1H, Ar*H*, overlapping with **2c**), 2.41 (s, 3H, CH_3), 1.20-1.08 (m, 6H, GeEt_3, overlapping with **2c**), 1.09-1.04 (m, 9H, GeEt_3).

^{13}C{^{1}H} NMR (151 MHz, $CDCl_3$): δ 139.4, 136.9, 131.5, 129.8, 21.3, 8.9, 4.1.

Mass Spectrometry: Calculated [$(M\text{-}Et)^+$] = 237.06950, Observed [$(M\text{-}Et)^+$] = 237.06907.

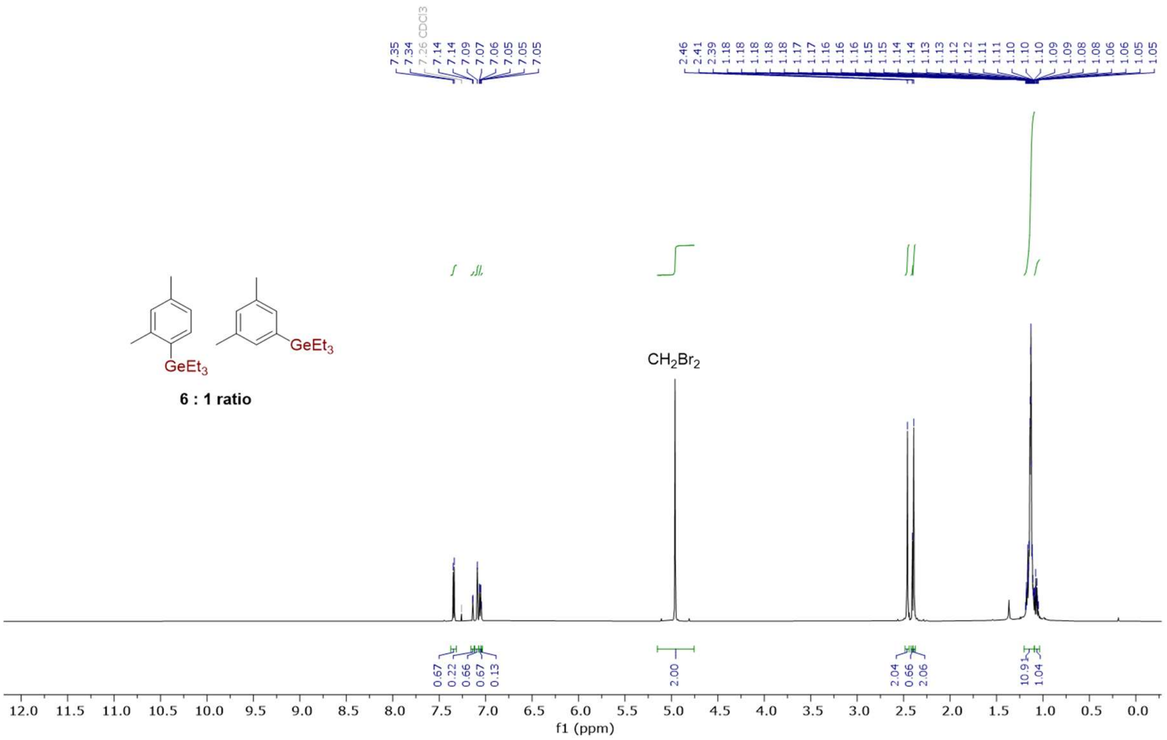

Figure S10: ^{1}H NMR spectrum of the two isomers of compound **2c** in $CDCl_3$.

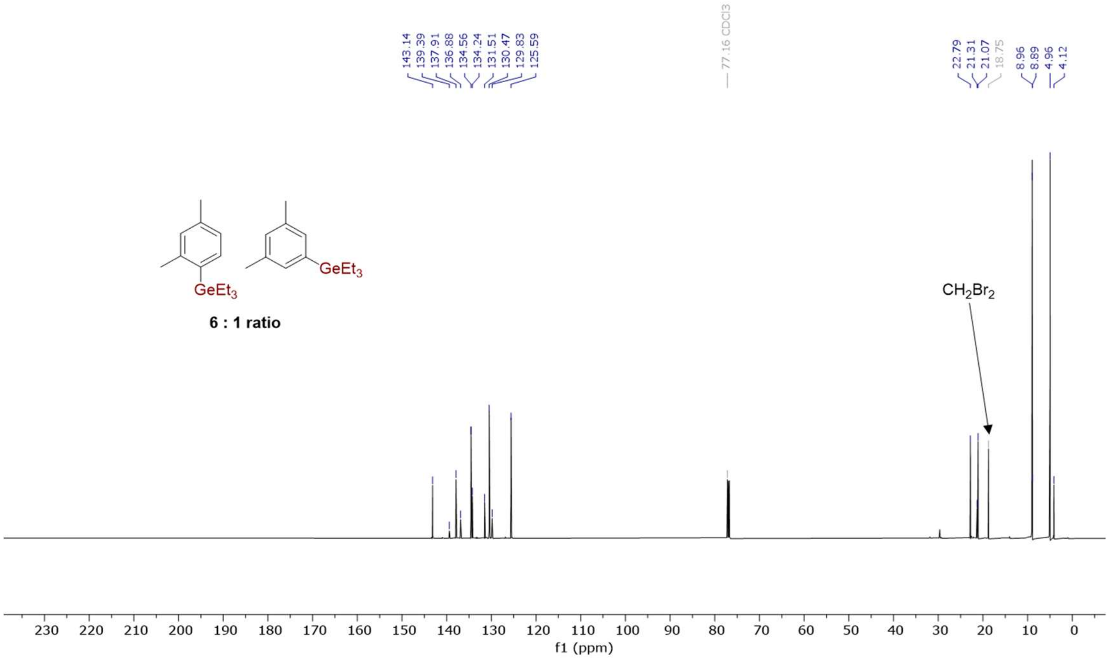

Figure S11: $^{13}C\{^{1}H\}$ NMR spectrum of the two isomers of compound **2c** in $CDCl_3$.

S2.2.4. C–H germylation of mesitylene

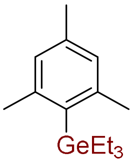

2d

As per general procedure 2, $GeEt_3$-mesityl (**2d**) was prepared using mesitylene (70 µL, 0.5 mmol, 1.0 equiv.) followed by extraction in n-hexane. **2d** was purified by column chromatography on silica gel using 40/60 petroleum ether (100%) eluent and isolated as a colourless oil. Yield: 92% (127 mg).

^{1}H NMR (500 MHz, $CDCl_3$): δ 6.82 (s, 2H, Ar*H*), 2.39 (s, 6H, o-CH_3), 2.26 (s, 3H, p-CH_3), 1.14-1.09 (m, 6H, GeEt_3), 1.07-1.04 (m, 9H, GeEt_3).

^{13}C{^{1}H} NMR (126 MHz, $CDCl_3$): δ 144.2, 137.7, 134.0, 128.6, 24.3, 20.8, 9.3, 7.9.

Mass Spectrometry: Calculated [$(M\text{-}Et)^+$] = 251. 08517, Observed [$(M\text{-}Et)^+$] = 251.08469.

These data are in agreement with those reported previously in the literature.[5]

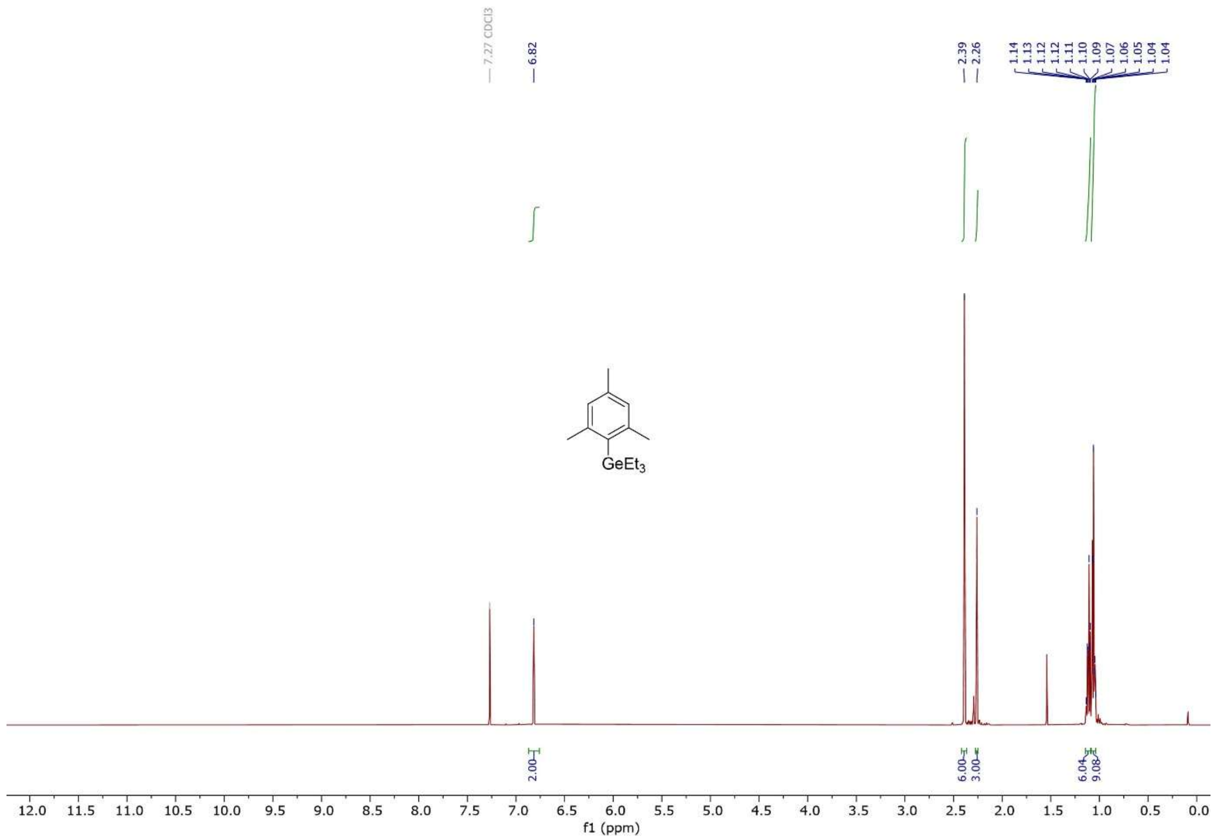

Figure S12: ^{1}H NMR spectrum of compound **2d** in $CDCl_3$.

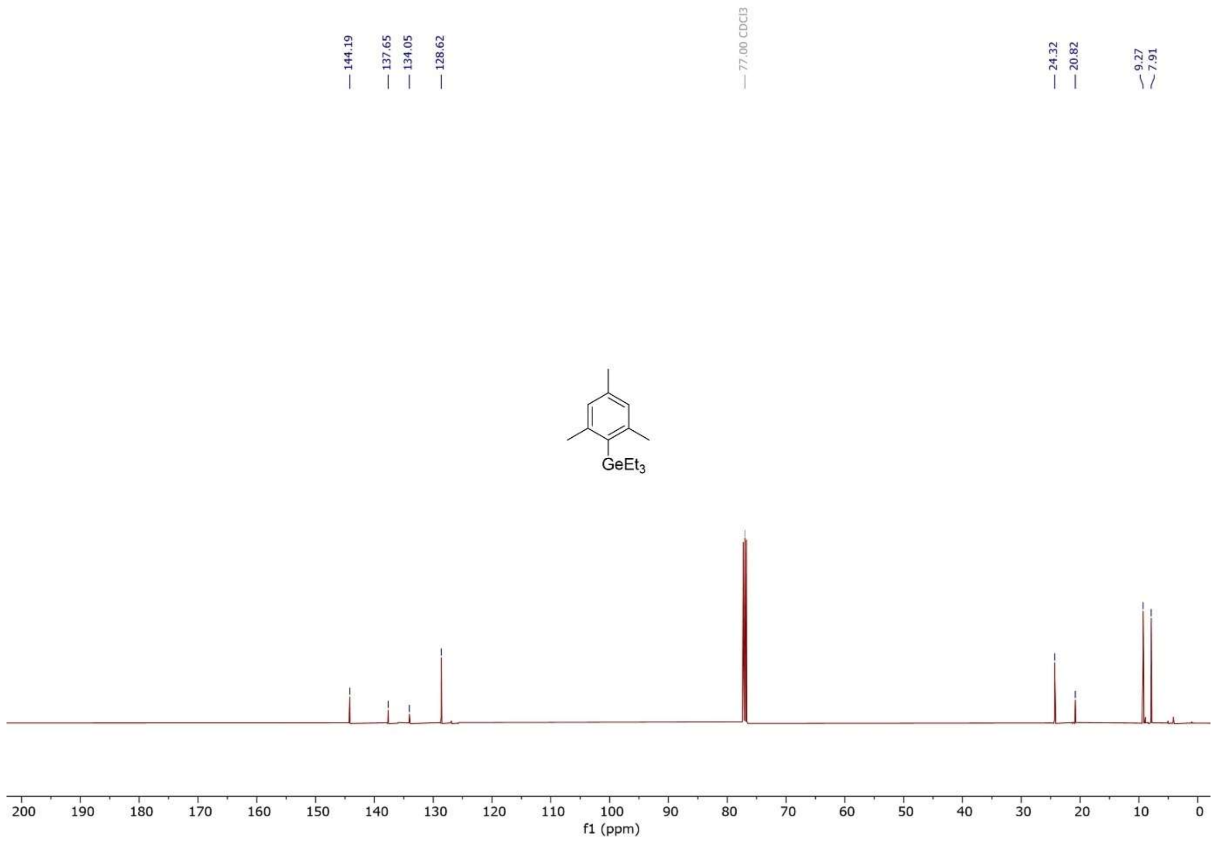

Figure S13: $^{13}C\{^{1}H\}$ NMR spectrum of compound **2d** in $CDCl_3$.

S2.2.5. C–H germylation of naphthalene

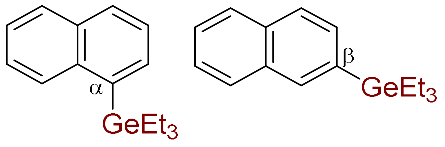

As per general procedure 2, α-GeEt3-naphthalene (**2e-α**) and β-GeEt3-naphthalene (**2e-β**) were prepared using naphthalene (64 mg, 0.5 mmol, 1.0 equiv.) followed by extraction in n-hexane. The crude reaction mixture was purified by column chromatography on silica gel using 40/60 petroleum ether (100%) eluent. **2e-α** and **2e-β** were isolated together in a 2:3 ratio as a pale-yellow oil. Combined yield: 80% (114 mg).

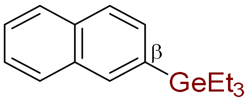

2e-β

As per modified general procedure 2, β-GeEt3-naphthalene (**2e-β**) was prepared using naphthalene (64 mg, 0.5 mmol, 1.0 equiv.), $AlCl_3$ (167 mg, 1.25 mmol, 2.5 eq.) and heating at 120 °C for 24 h followed by extraction in n-pentane and the standard workup. This yielded 56% (vs internal standard) of **2e-α** and **2e-β** in a ca. 1:6 ratio The crude reaction mixture was purified by column chromatography on silica gel using 40/60 petroleum ether (100%) eluent. **2e-β** was isolated as a pale-yellow oil. Yield: 35% (50 mg). *Note:* **2e-β** was isolated in a 6:1 ratio with **2e-α**.

2e-α

^{1}H NMR (500 MHz, $CDCl_3$): δ 8.06-7.99 (m, 1H, Ar*H*), 7.91-7.82 (m, 2H, Ar*H*, overlapping with **2e-β**), 7.58 (dd, *J* = 8.1, 1.1 Hz, 1H, Ar*H*), 7.55-7.44 (m, 3H, Ar*H*, overlapping with **2e-β**), 1.25-1.10 (m, 15H, Ge*Et*3, overlapping with **2e-β**).

^{13}C{^{1}H} NMR (126 MHz, $CDCl_3$): δ 138.5, 137.7, 133.8, 133.3, 129.1, 128.9, 128.3, 125.7, 125.4, 125.3, 9.3, 5.7.

These data are in agreement with those reported previously in the literature.[3]

2e-β

^{1}H NMR (500 MHz, CDCl$_3$): 7.93 (s, 1H, Ar*H*), 7.85-7.81 (m, 3H, Ar*H*), 7.55 (dd, *J* = 8.1, 1.1 Hz, 1H, Ar*H*), 7.49-7.46 (m, 2H, Ar*H*), 1.11-1.07 (m, 15H, Ge*Et*$_3$, overlapping with **2e-α**).

^{13}C{^{1}H} NMR (126 MHz, CDCl$_3$): δ 137.6, 134.2, 133.5, 133.3, 131.0, 128.0, 127.9, 127.0, 126.0, 125.9, 9.1, 4.4.

Mass Spectrometry: Calculated [(M-Et)$^+$] = 259.05389, Observed [(M-Et)$^+$] = 259.05340.

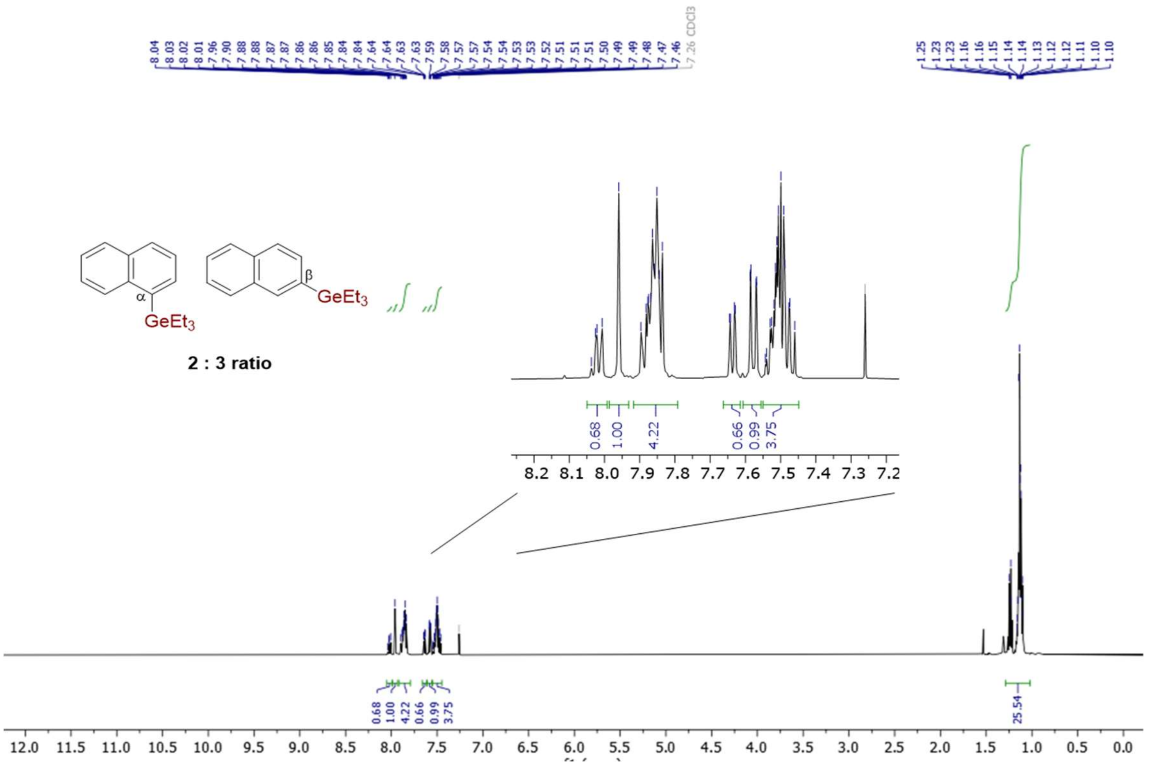

Figure S14: ^{1}H NMR spectrum of the two isomers of compound **2e** in CDCl$_3$.

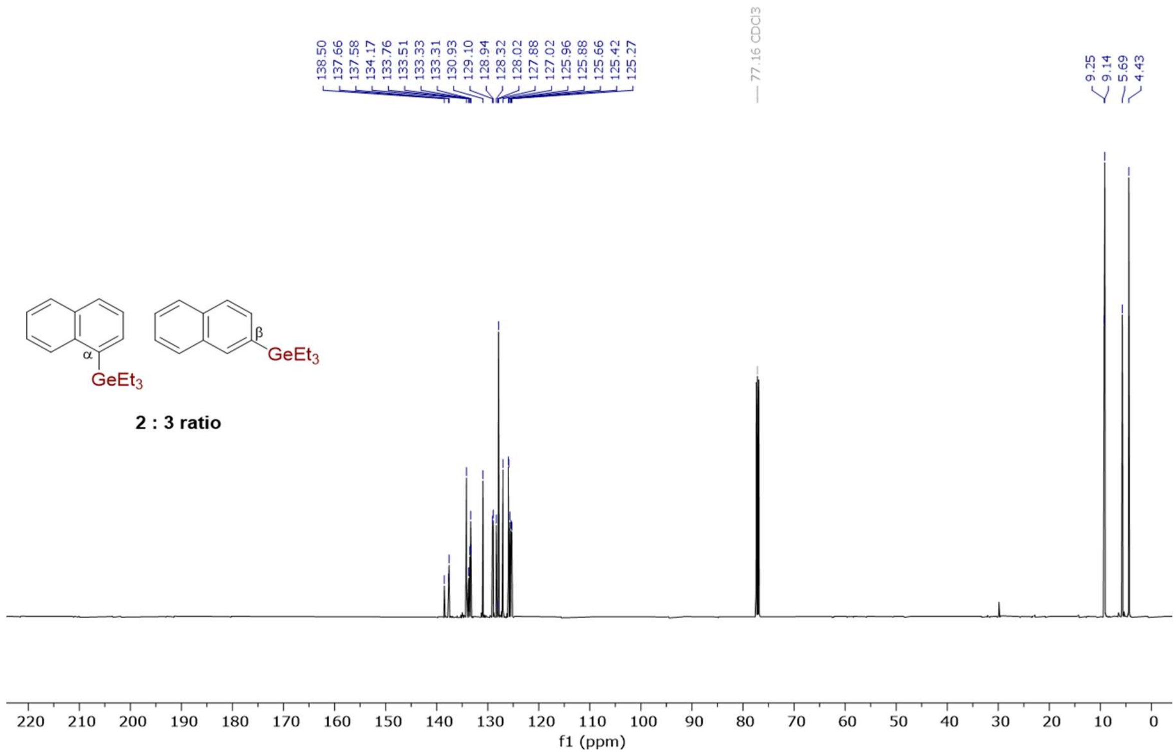

Figure S15: $^{13}C\{^{1}H\}$ NMR spectrum of the two isomers of compound **2e** in $CDCl_3$.

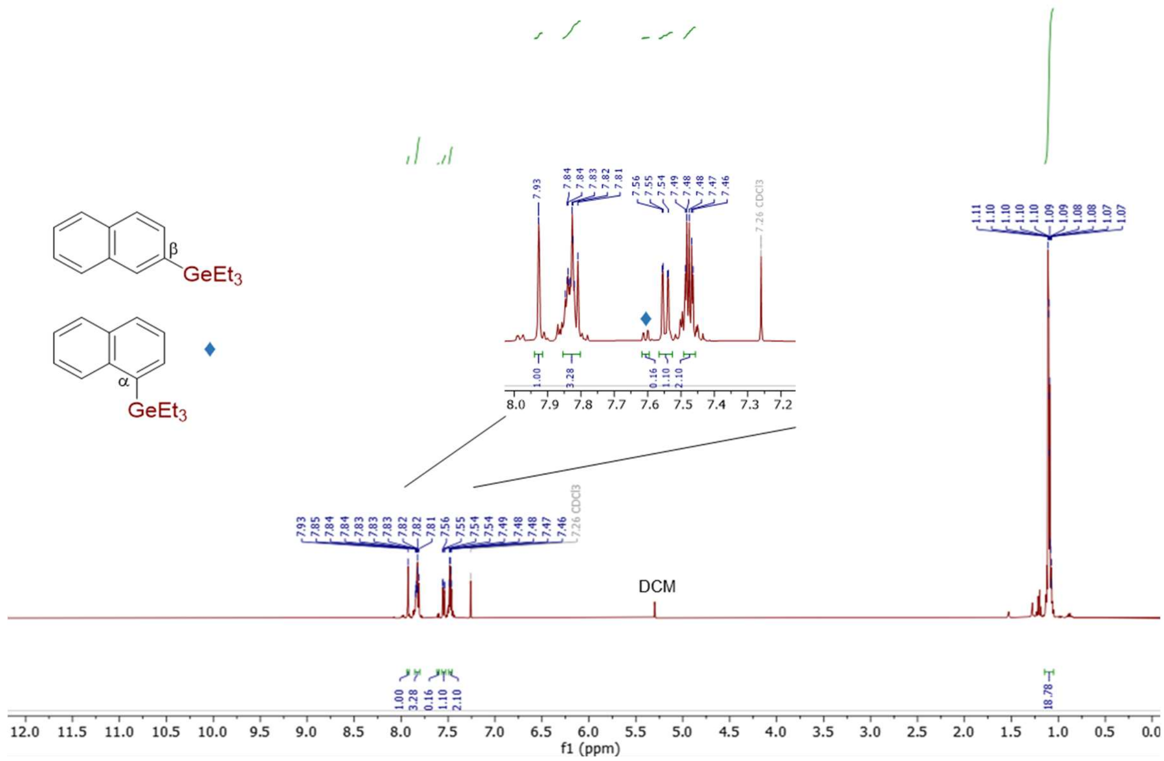

Figure S16: ^{1}H NMR spectrum of compound **2e-β** in $CDCl_3$ (contains ca. 14% of **2e-α**).

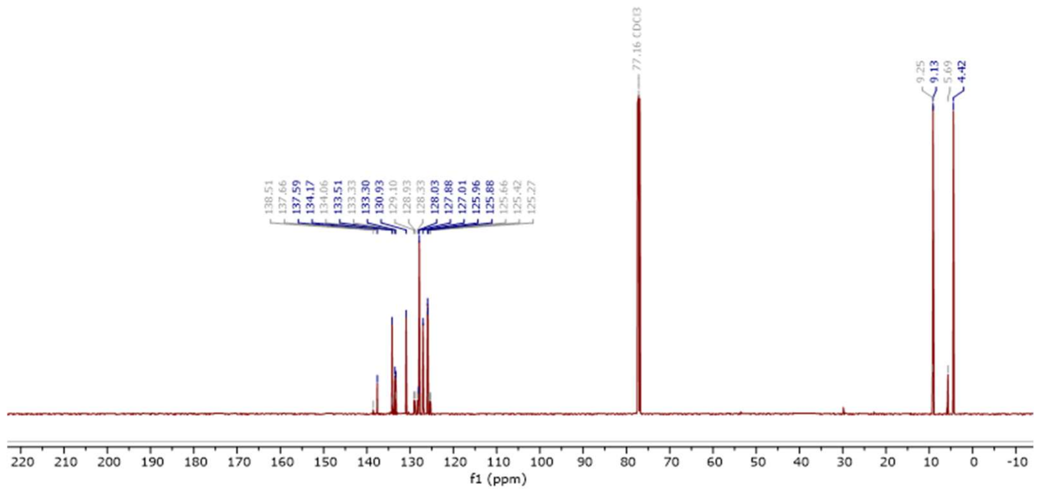

Figure S17: $^{13}C\{^1H\}$ NMR spectrum of compound **2e-β** in $CDCl_3$. Peaks annotated in grey were assigned to **2e-α**.

S2.2.6. C–H germylation of fluorobenzene

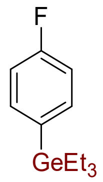

2f
(p:o:m, 78:16:6)

As per modified general procedure 2, p-$GeEt_3$-fluorobenzene (**2f**) was prepared using fluorobenzene (47 µL, 0.5 mmol, 1.0 equiv.) and heating at 120 °C, followed by extraction in n-pentane. **2f** was purified by column chromatography on silica gel using 40/60 petroleum ether (100%) eluent and isolated (p:o:m, 78:16:6) as a colourless oil. Yield: 46% (59 mg).

^{1}H NMR (500 MHz, $CDCl_3$): δ 7.40 (dd, J = 8.5, 6.4 Hz, 2H, ArH), 7.05 (dd, J = 9.5, 8.6 Hz, 2H, ArH), 1.10-1.03 (m, 9H, GeEt_3), 1.03-0.95 (m, 6H, GeEt_3).

$^{13}C\{^1H\}$ NMR (126 MHz, $CDCl_3$): δ 163.4 (d, J = 246.3 Hz), 135.7 (d, J = 7.0 Hz), 135.0 (d, J = 4.0 Hz), 115.1 (d, J = 19.4 Hz), 9.0, 4.4.

^{19}F NMR (471 MHz, $CDCl_3$): δ –113.6 - –113.9 (m).

Mass Spectrometry: Calculated $[(M\text{-}Et)^+]$ = 227.02874, Observed $[(M\text{-}Et)^+]$ = 227.02835.

These data are in agreement with those reported previously in the literature.[3]

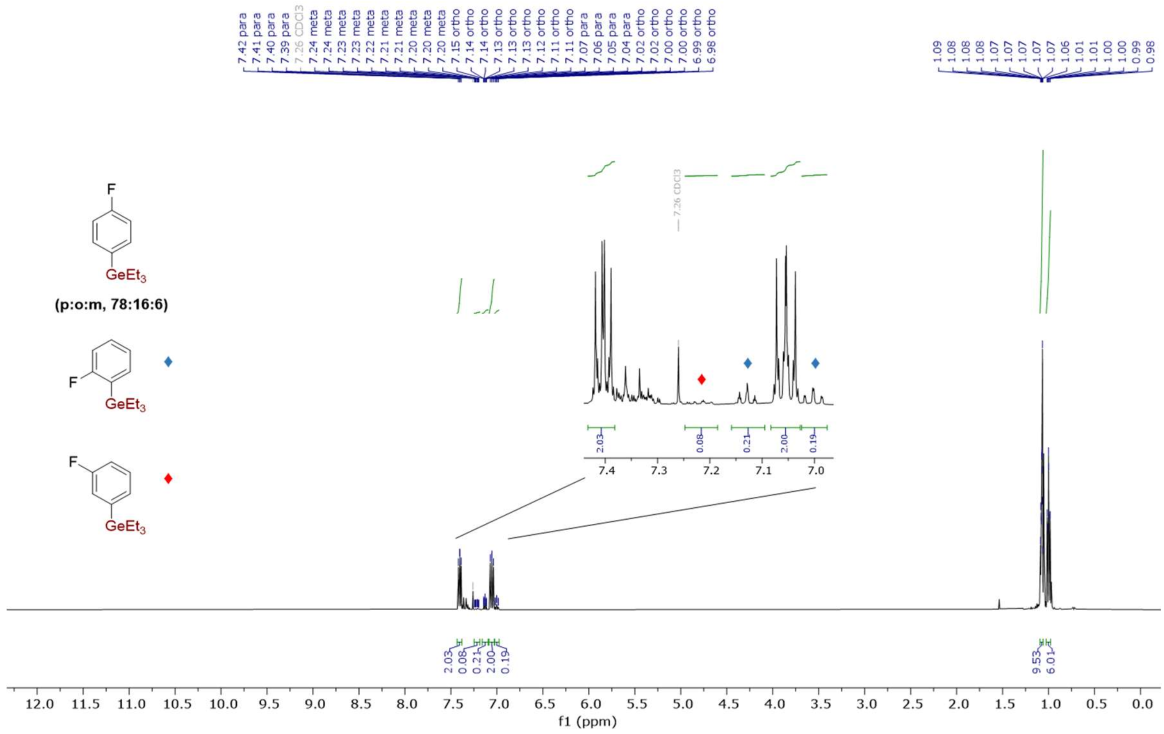

Figure S18: ^{1}H NMR spectrum of compound **2f** in $CDCl_3$.

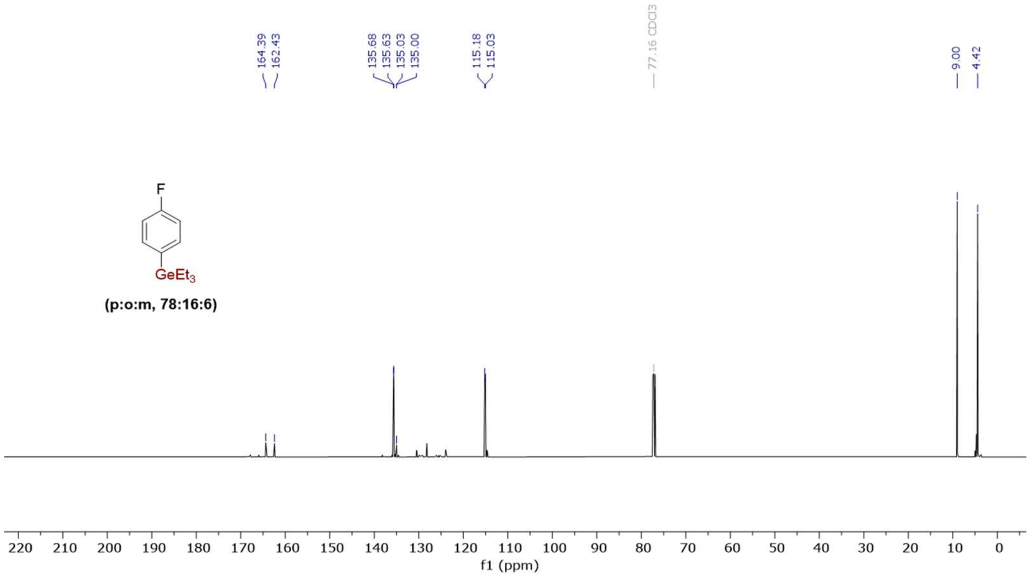

Figure S19: $^{13}C\{^1H\}$ NMR spectrum of compound **2f** in $CDCl_3$.

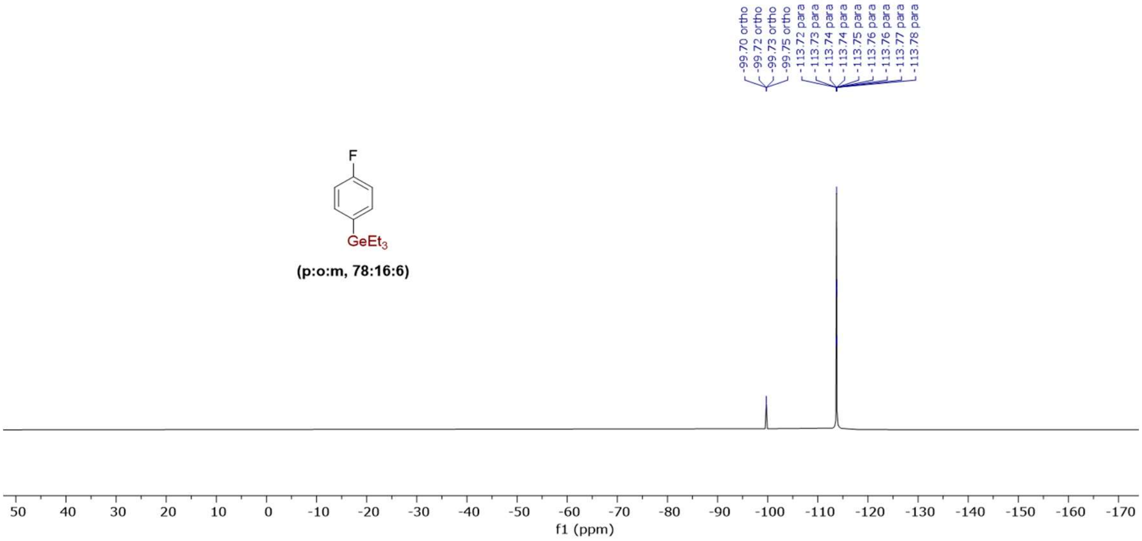

Figure S20: ^{19}F NMR spectrum of compound **2f** in $CDCl_3$.

S2.2.7. C–H germylation of benzene

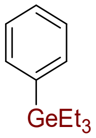

2g

As per general procedure 2, $GeEt_3$-phenyl (**2g**) was prepared using benzene (45 µL, 0.5 mmol, 1.0 equiv.), and heating to 120°C, with extraction in n-hexane. **2g** was purified by column chromatography on silica gel using 40/60 petroleum ether (100%) eluent and isolated as a colourless oil. Yield: 16% (19 mg). $GeEt_2$-phenyl$_2$ (**3g**) was also isolated in 46% yield (33 mg).

As per modified general procedure 2, $GeEt_3$-phenyl (**2g**) was prepared using benzene (45 µL, 0.5 mmol, 1.0 equiv.), $AlCl_3$ (167 mg, 1.25 mmol, 2.5 eq.) and heating at 120 °C, followed by extraction in n-pentane. **2g** was purified by column chromatography on silica gel using 40/60 petroleum ether (100%) eluent and isolated as a colourless oil. Yield: 72% (85 mg).

^{1}H NMR (500 MHz, $CDCl_3$): δ 7.47-7.41 (m, 2H, Ar*H*), 7.38-7.30 (m, 3H, Ar*H*), 1.11-1.03 (m, 9H, Ge*Et*$_3$), 1.03-0.96 (m, 6H, Ge*Et*$_3$).

^{13}C{^{1}H} NMR (126 MHz, $CDCl_3$): δ 140.0, 134.1, 128.2, 128.0, 9.1, 4.3.

Mass Spectrometry: Calculated [(M-Et)$^+$] = 209.03813 ($C_{10}H_{15}Ge$), Observed [(M-Et)$^+$] = 209.03816.

These data are in agreement with those reported previously in the literature.[5]

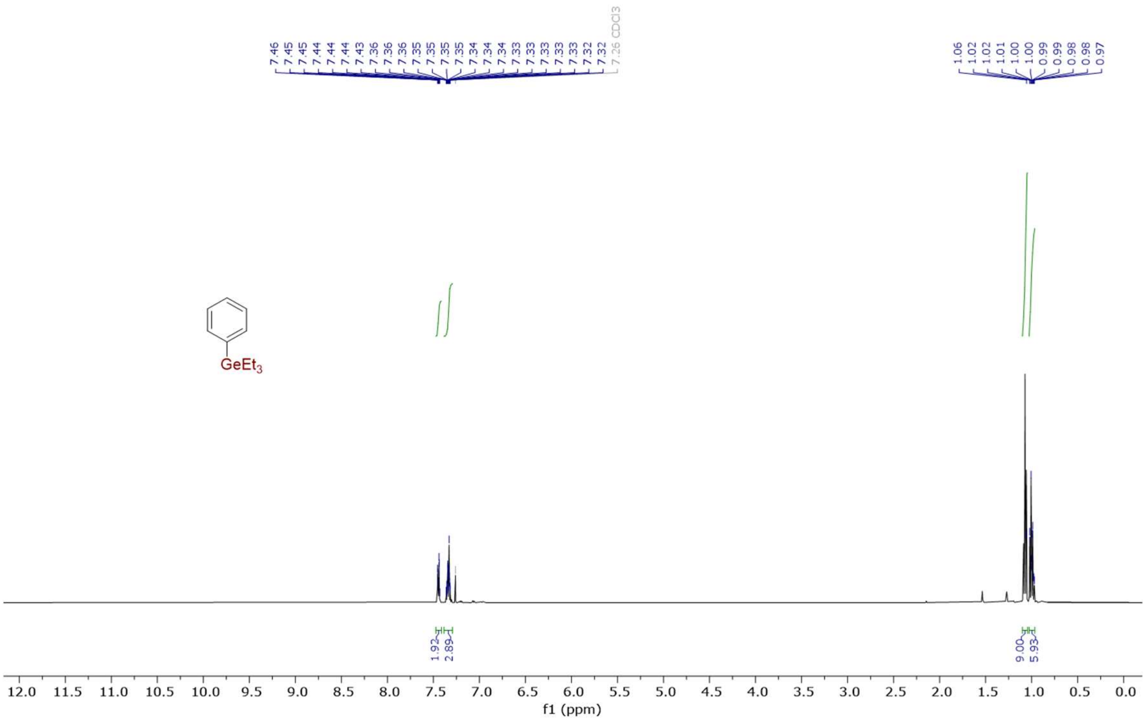

Figure S21: ^{1}H NMR spectrum of compound **2g** in $CDCl_3$.

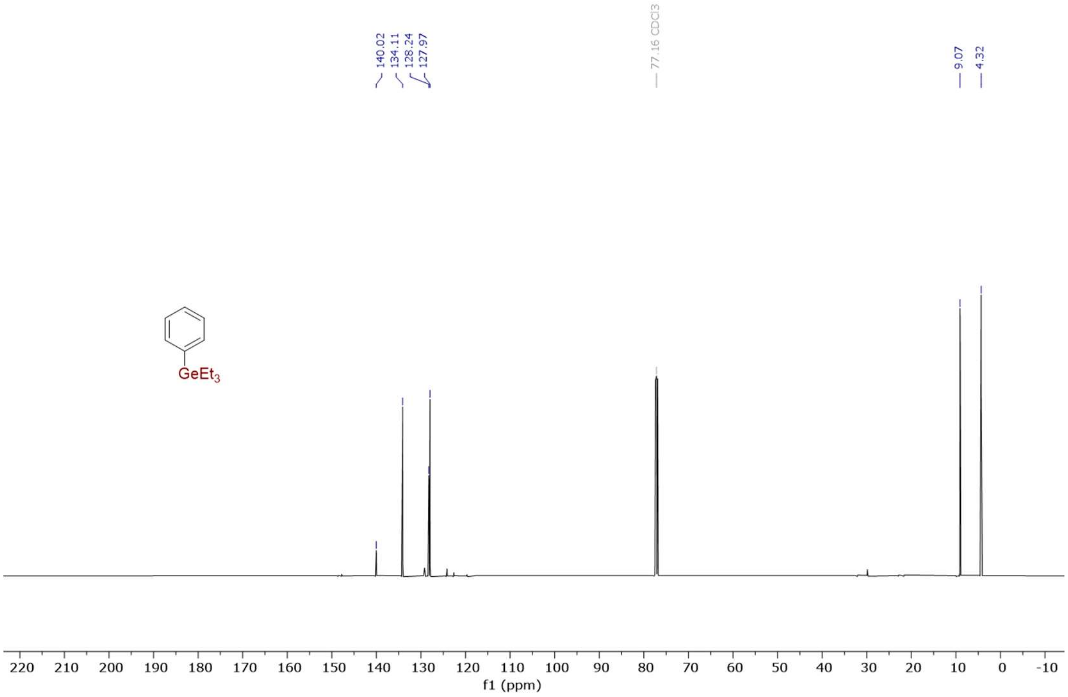

Figure S22: $^{13}C\{^{1}H\}$ NMR spectrum of compound **2g** in $CDCl_3$.

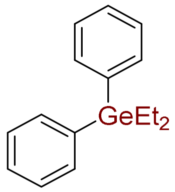

3g

As per modified general procedure 2, GeEt$_2$-phenyl$_2$ (**3g**) was prepared using benzene (45 μL, 0.5 mmol, 1.0 equiv.), GeCl$_4$ (25 μL, 0.22 mmol, 0.45 eq.) and heating at 80 °C for 48 h, followed by extraction in n-pentane and the addition of EtMgBr solution (3.0 M solution in E$_2$O, 1.2 mmol., 2.4 equiv. w.r.t. arene). Crude **3g** was obtained with 98% yield (61 mg) containing 11% of **2g**. **3g** was further purified by column chromatography on silica gel using 40/60 petroleum ether (100%) eluent and isolated as a colourless oil. Yield: 56% (35 mg). *Note:* GeEt$_3$-phenyl (**2g**) was also isolated in 8% yield (4 mg).

^{1}H NMR (500 MHz, CDCl$_3$): δ 7.50-7.45 (m, 4H, Ar*H*), 7.39-7.34 (m, 6H, Ar*H*), 1.28 (q, J = 7.4, 4H, Ge*Et*$_2$), 1.13 (t, J = 7.5, 6H, Ge*Et*$_2$).

^{13}C{^{1}H} NMR (126 MHz, CDCl$_3$): δ 138.5, 134.6, 128.6, 128.1, 9.1, 5.2.

Mass Spectrometry: Calculated [(M-Et)$^+$] = 257.03800 ($C_{14}H_{15}Ge$), Observed [(M-Et)$^+$] = 257.03796.

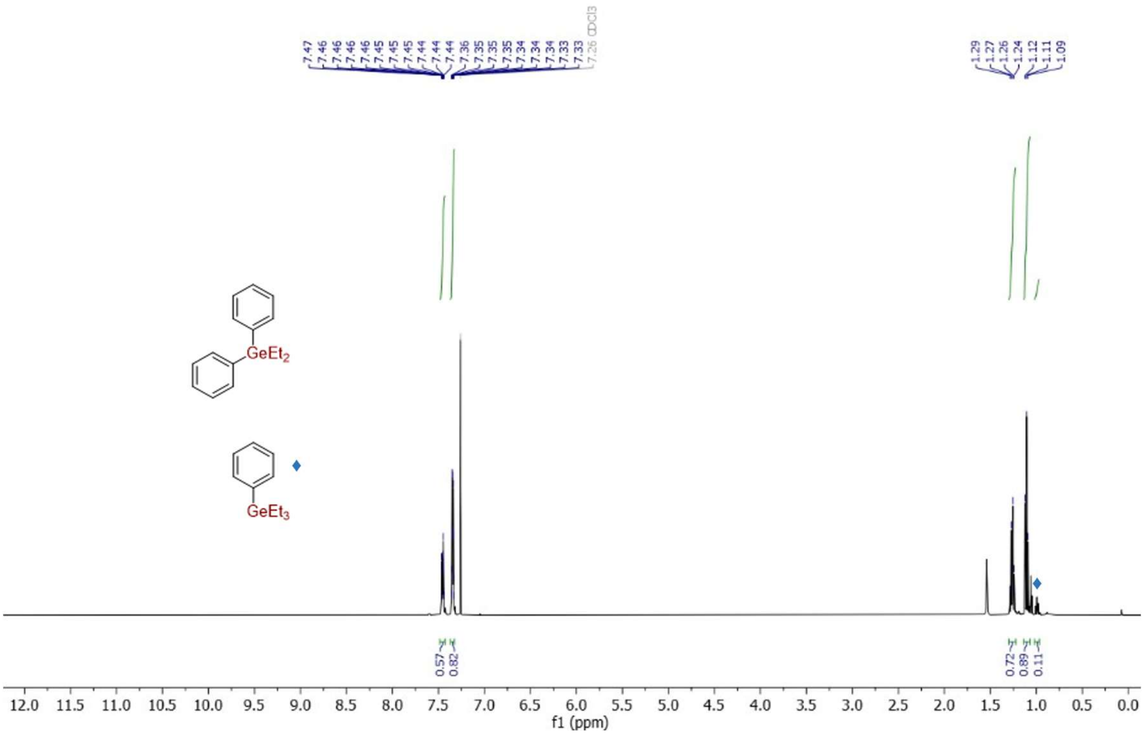

Figure S23: Crude ^{1}H NMR spectrum of compound **3g** in $CDCl_3$ (contains 11% of **2g**).

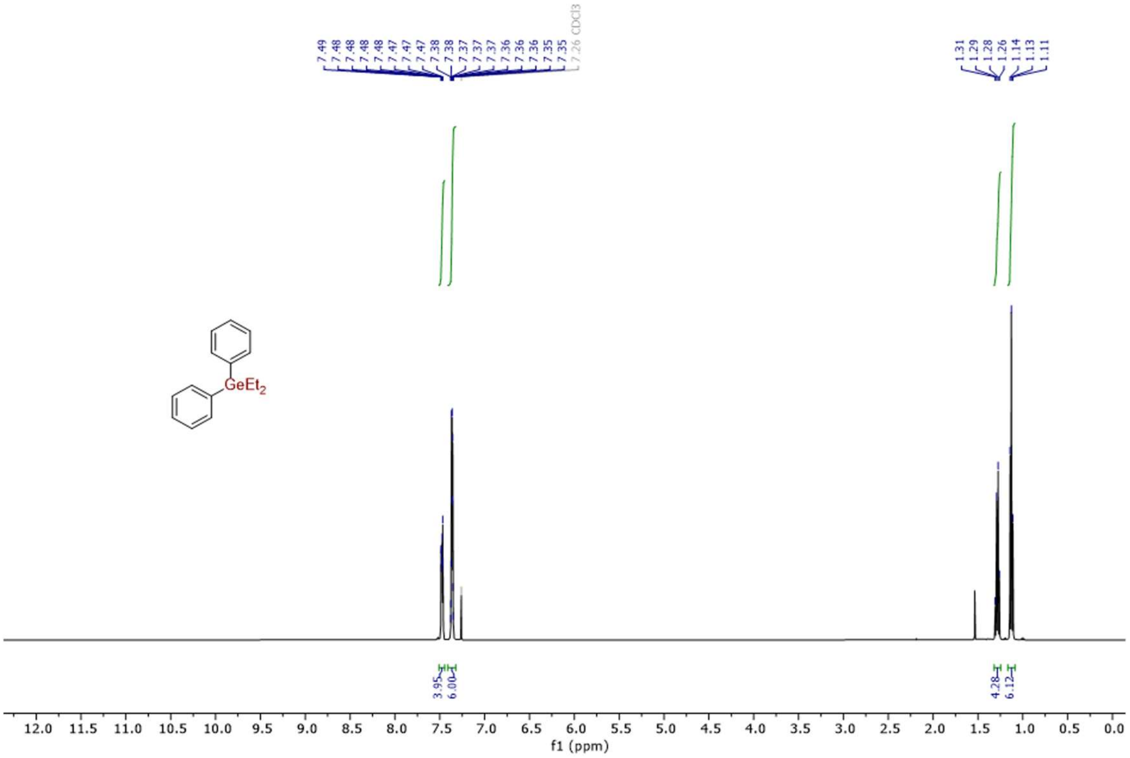

Figure S24: ^{1}H NMR spectrum of compound **3g** in $CDCl_3$.

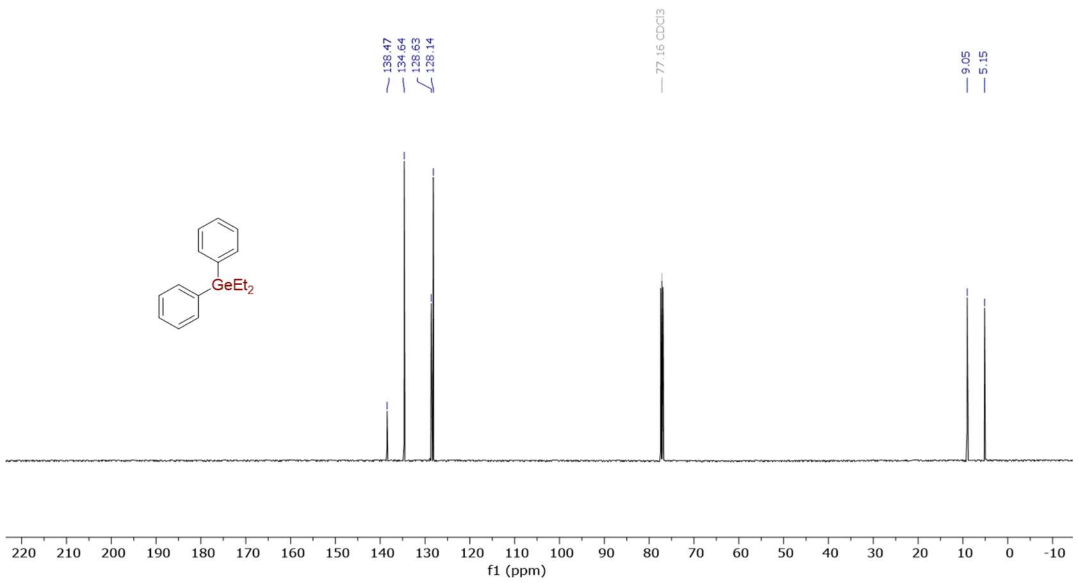

Figure S25: $^{13}C\{^{1}H\}$ NMR spectrum of compound **3g** in $CDCl_3$.

S2.2.8. C–H germylation of chlorobenzene

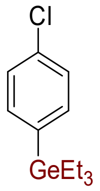

2h

As per modified general procedure 2, p-$GeEt_3$-chlorobenzene (**2h**) was prepared using chlorobenzene (50 µL, 0.5 mmol, 1.0 equiv.) and heating at 125 °C, followed by extraction in n-hexane. **2h** was purified by column chromatography on silica gel using 40/60 petroleum ether (100%) eluent and isolated as a colourless oil. Yield: 29% (39 mg). *Note:* $GeEt_2$-(p-chlorobenzene)$_2$ (**3h**) was also isolated in 18% yield (16 mg).

As per modified general procedure 2, p-$GeEt_3$-chlorobenzene (**2h**) was prepared using chlorobenzene (50 µL, 0.5 mmol, 1.0 equiv.), $AlCl_3$ (167 mg, 1.25 mmol, 2.5 eq.) and heating at 125 °C, followed by extraction in n-pentane. **2h** was purified by column chromatography on silica gel using 40/60 petroleum ether (100%) eluent and isolated as a colourless oil. Yield: 41% (54 mg). *Note:* $GeEt_2$-(p-chlorobenzene)$_2$ (**3h**) was also isolated in 11% yield (10 mg).

^{1}H NMR (500 MHz, CDCl$_3$): δ 7.35 (d, J = 8.3 Hz, 2H, ArH), 7.31 (d, J = 8.5 Hz, 2H, ArH), 1.07-1.02 (m, 9H, GeEt_3), 1.02-0.93 (m, 6H, GeEt_3).

^{13}C{^{1}H} NMR (126 MHz, CDCl$_3$): δ 138.2, 135.4, 134.5, 128.2, 9.0, 4.3.

Mass Spectrometry: Calculated [(M-Et)$^+$] = 242.99903 ($C_{10}H_{14}ClGe$), Observed [(M-Et)$^+$] = 242.99903.

These data are in agreement with those reported previously in the literature.[3]

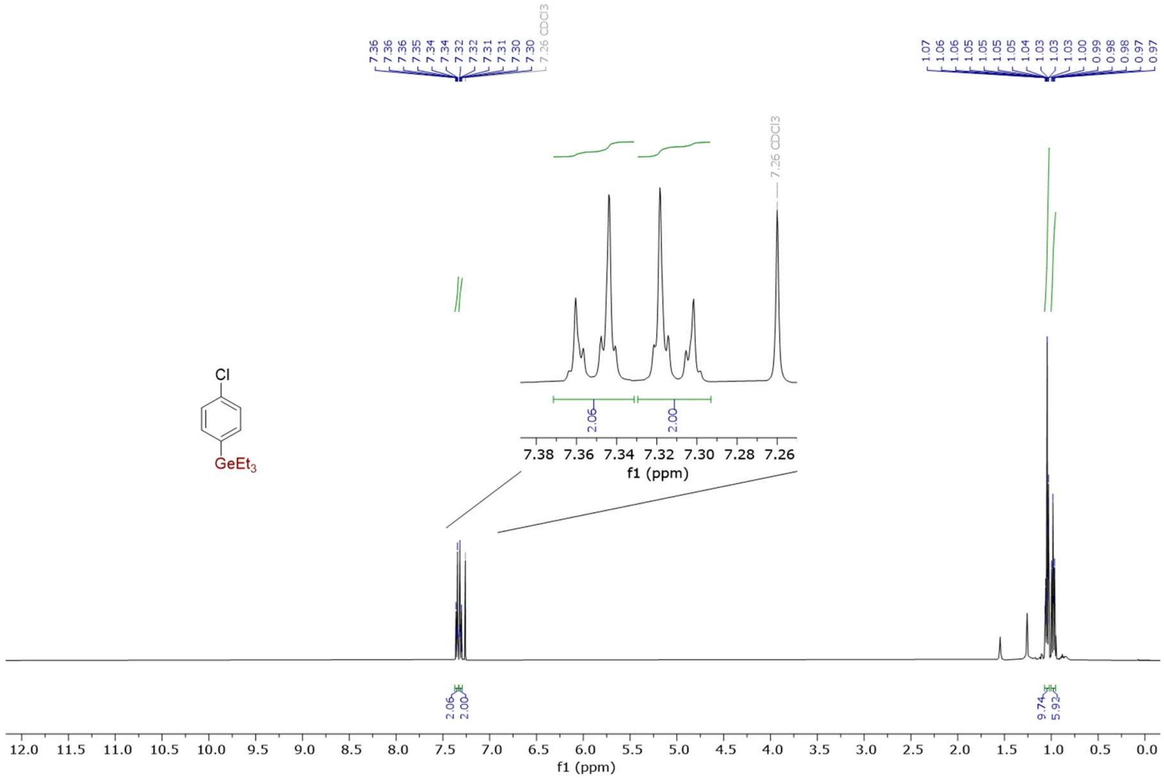

Figure S26: ^{1}H NMR spectrum of compound **2h** in CDCl$_3$.

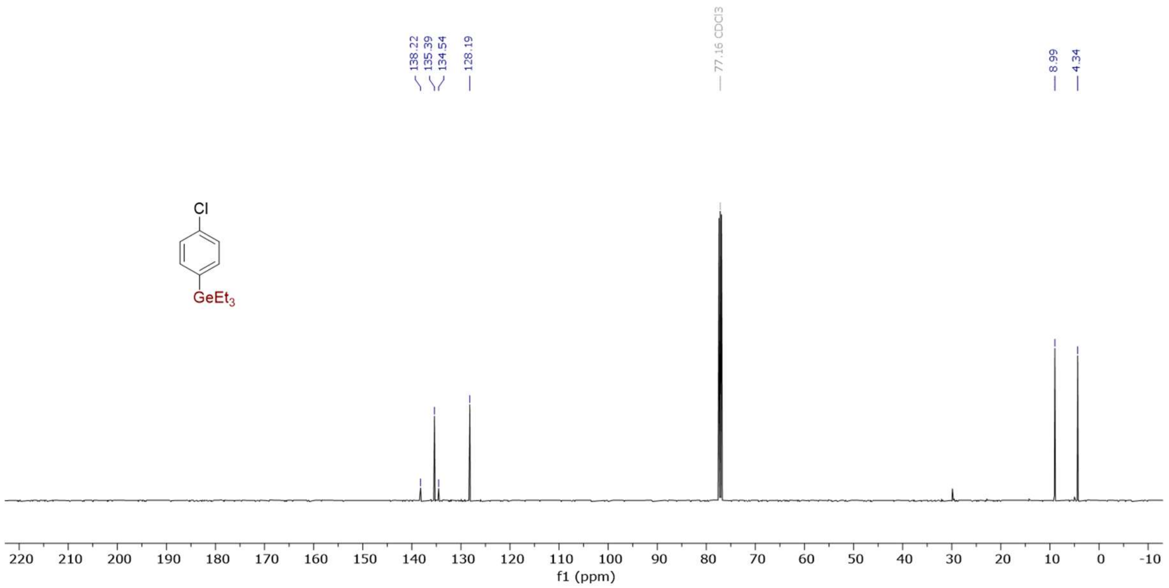

Figure S27: $^{13}C\{^{1}H\}$ NMR spectrum of compound **2h** in $CDCl_3$.

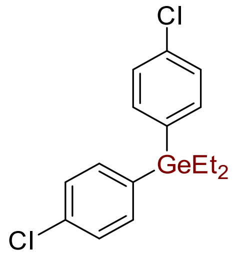

3h

Isolated in low yields (18% yield, 16 mg; 11% yield 10 mg). during the C–H germylation of chlorobenzene (see earlier).

^{1}H NMR (500 MHz, $CDCl_3$): δ 7.34 (apparent d, J = 1.6 Hz, 8H, ArH), 1.25 (qd, J = 7.6, 1.1 Hz, 4H, GeEt_2), 1.13-1.06 (m, 6H, GeEt_2).

$^{13}C\{^{1}H\}$ NMR (126 MHz, $CDCl_3$): δ 136.2, 135.8, 135.2, 128.5, 8.9, 5.2.

Mass Spectrometry: Calculated [$(M\text{-}Et)^{+}$] = 324.96071 ($C_{14}H_{13}Cl_2Ge$), Observed [$(M\text{-}Et)^{+}$] = 324.96094.

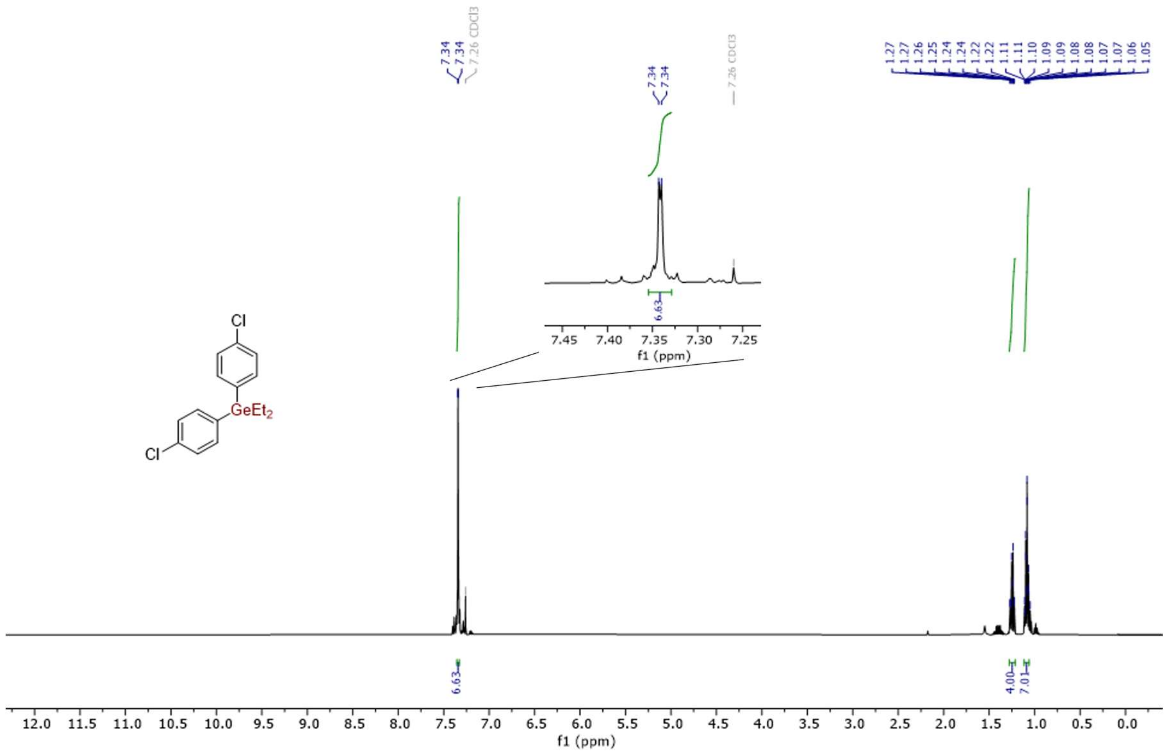

Figure S28: ^{1}H NMR spectrum of compound **3h** in $CDCl_3$.

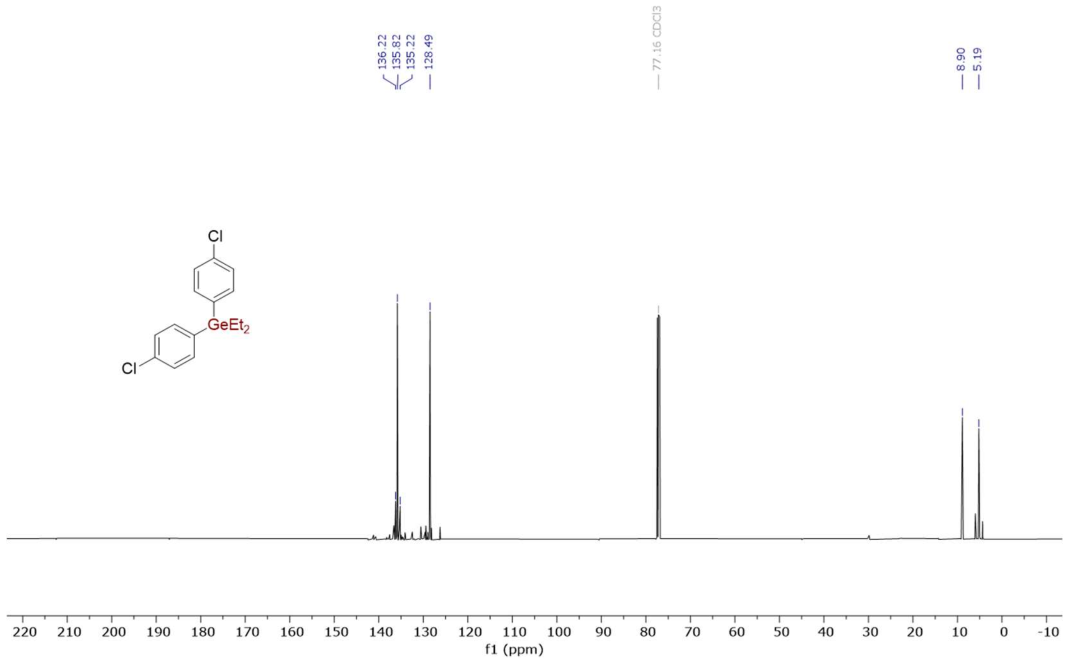

Figure S29: $^{13}C\{^{1}H\}$ NMR spectrum of compound **3h** in $CDCl_3$.

S2.2.9. C–H germylation of bromobenzene

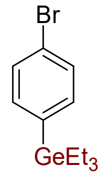

2i

As per modified general procedure 2, p-GeEt$_3$-bromobenzene (**2i**) was prepared using bromobenzene (52.5 µL, 0.5 mmol, 1.0 equiv.) and heating at 125 °C, followed by extraction in n-hexane. **2i** was purified by column chromatography on silica gel using 40/60 petroleum ether (100%) eluent and isolated as a colourless oil. Yield: 18% (28 mg). *Note:* GeEt$_2$-(p-bromobenzene)$_2$ (**3i**) was also isolated in 19% yield (21 mg).

^{1}H NMR (500 MHz, CDCl$_3$): δ 7.47 (dt, J = 8.4, 1.9 Hz, 2H, ArH), 7.30 (dt, J = 8.3, 1.9 Hz, 2H, ArH), 1.08-1.03 (m, 9H, GeEt_3), 1.02-0.95 (m, 6H, GeEt_3).

^{13}C{^{1}H} NMR (126 MHz, CDCl$_3$): δ 138.8, 135.7, 131.1, 123.0, 9.0, 4.3.

Mass Spectrometry: Calculated [(M-Et)$^+$] = 286.94815, Observed [(M-Et)$^+$] = 286.94873.

These data are in agreement with those reported previously in the literature.[6]

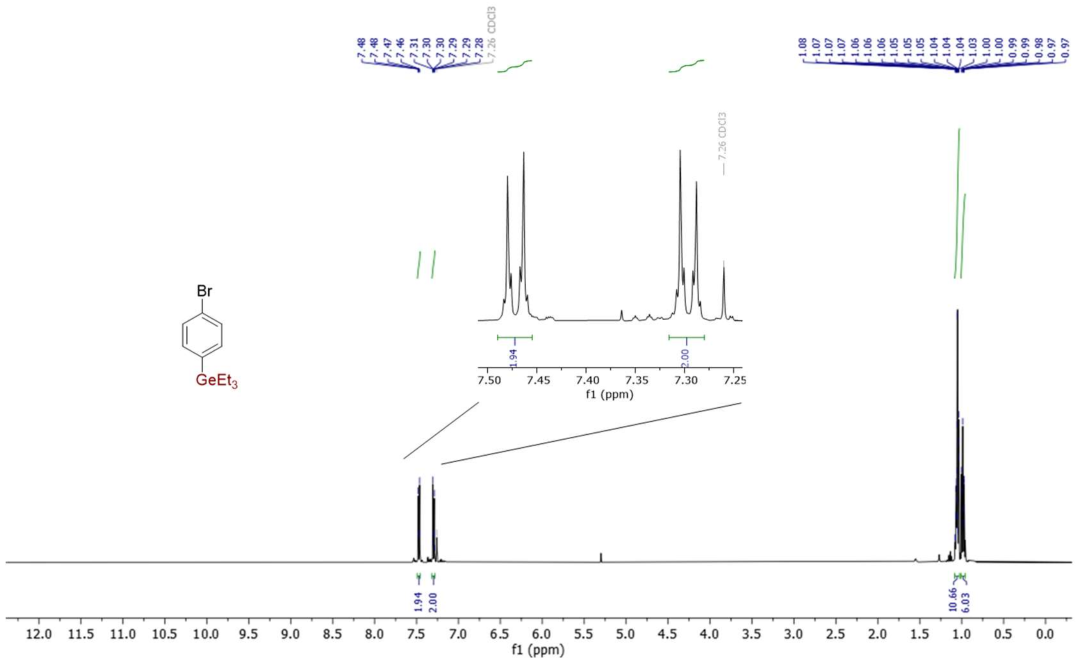

Figure S30: ^{1}H NMR spectrum of compound **2i** in CDCl$_3$.

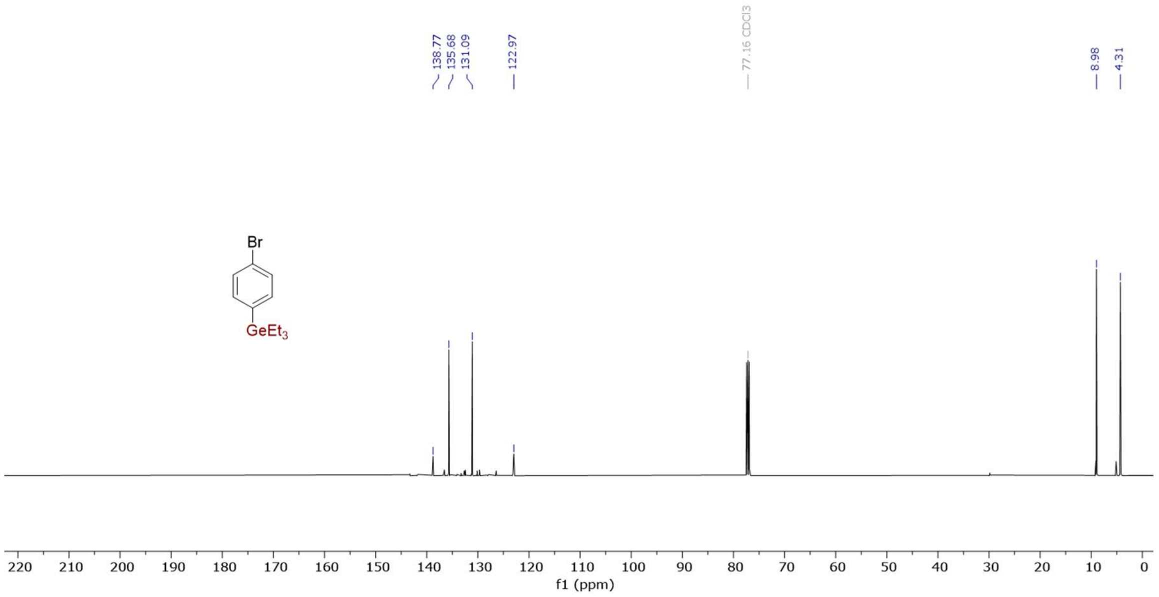

Figure S31: $^{13}C\{^{1}H\}$ NMR spectrum of compound **2i** in $CDCl_3$.

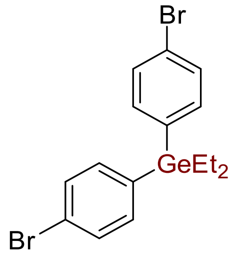

3i

Isolated in low yield (19% yield, 21 mg) during the C–H germylation of bromobenzene (see earlier).

^{1}H NMR (500 MHz, $CDCl_3$): δ 7.49 (dt, J = 8.4, 1.8 Hz, 4H, ArH), 7.28 (dt, J = 8.3, 1.9 Hz, 4H, ArH), 1.24 (qd, J = 7.6, 1.0 Hz, 4H, GeEt_2), 1.12-1.03 (m, 6H, GeEt_2).

$^{13}C\{^{1}H\}$ NMR (126 MHz, $CDCl_3$): δ 136.7, 136.1, 131.4, 123.7, 8.9, 5.1.

Mass Spectrometry: Calculated [(M-Et)$^+$] = 412.85841, Observed [(M-Et)$^+$] = 412.85822.

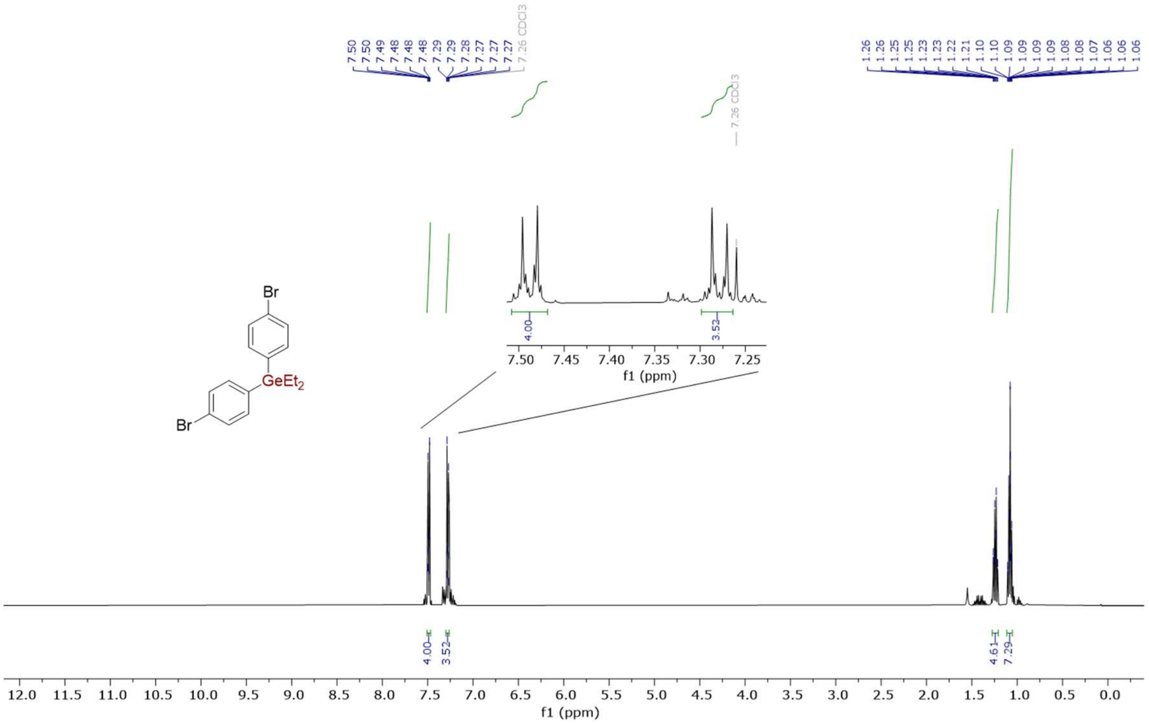

Figure S32: ^{1}H NMR spectrum of compound **3h** in $CDCl_3$.

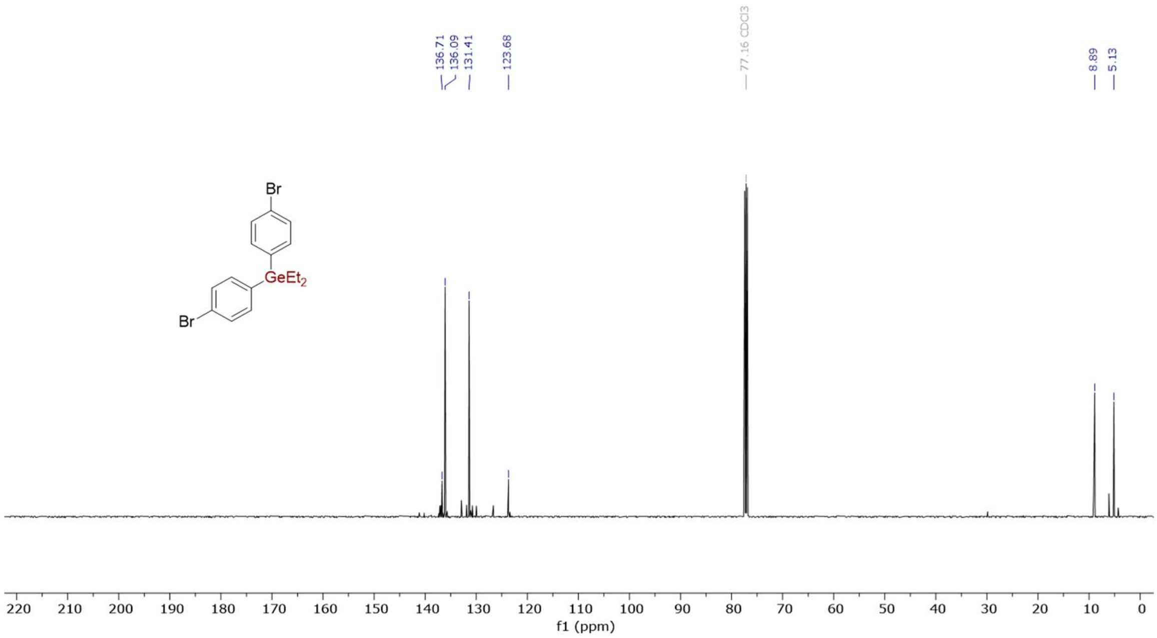

Figure S33: $^{13}C\{^{1}H\}$ NMR spectrum of compound **3h** in $CDCl_3$.

S2.2.10. C–H germylation of fluorotoluene

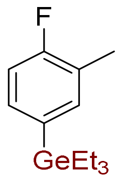

2j

As per modified general procedure 2, p-GeEt$_3$-fluorotoluene (**2j**) was prepared using o-fluorotoluene (55 µL, 0.5 mmol, 1.0 equiv.), followed by extraction in n-pentane. **2i** was purified by manual column chromatography on silica gel using 40/60 petroleum ether (100%) eluent and isolated as a colourless oil. Yield: 15% (21 mg). *Note:* GeEt$_2$-(p-fluorotoluene)$_2$ (**3j**) was also isolated in 59% yield (51 mg).

^{1}H NMR (400 MHz, CDCl$_3$): δ 7.22-7.18 (m, 2H, Ar*H*), 6.98 (dd, *J* = 9.9, 8.5 Hz, 1H, Ar*H*), 2.28 (d, *J* = 2.0 Hz, 3H, C*H$_3$*), 1.06-1.03 (m, 9H, Ge*Et$_3$*), 0.99-0.95 (m, 6H, Ge*Et$_3$*).

^{1}H{^{19}F} NMR (400 MHz, CDCl$_3$): δ 7.21 (s, 1H, Ar*H*), 7.20 (d, *J* = 8.5 Hz, 1H, Ar*H*, overlapping with the singlet at 7.21), 6.98 (d, *J* = 8.5 Hz, 1H, Ar*H*), 2.28 (s, 3H, C*H$_3$*), 1.07-1.03 (m, 9H, Ge*Et$_3$*), 0.99-0.95 (m, 6H, Ge*Et$_3$*).

^{13}C{^{1}H} NMR (126 MHz, CDCl$_3$): δ 162.0 (d, *J* = 245.3 Hz), 137.1 (d, *J* = 4.5 Hz), 134.7 (d, *J* = 4.5 Hz), 133.0 (d, *J* = 7.5 Hz), 124.4 (d, *J* = 15.5 Hz), 114.7 (d, *J* = 20.9 Hz), 14.7 (d, *J* = 4.0 Hz), 9.0, 4.4.

^{19}F NMR (471 MHz, CDCl$_3$): δ –118.2 - –118.3 (m).

Mass Spectrometry: Calculated [(M-Et)$^+$] = 241.04441, Observed [(M-Et)$^+$] = 241.04403.

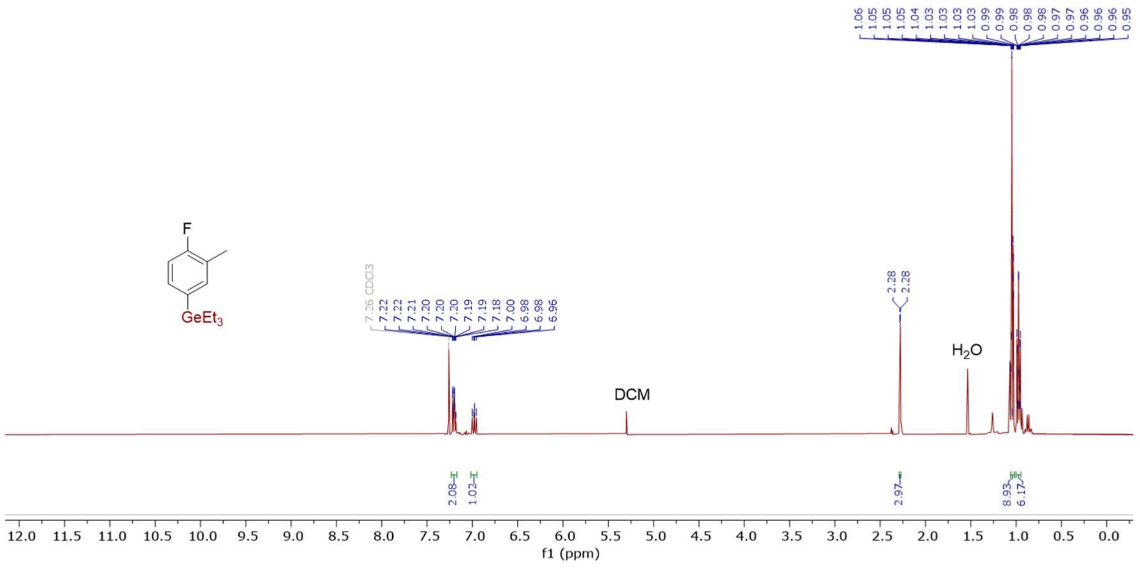

Figure S34: ^{1}H NMR spectrum of compound **2j** in $CDCl_3$.

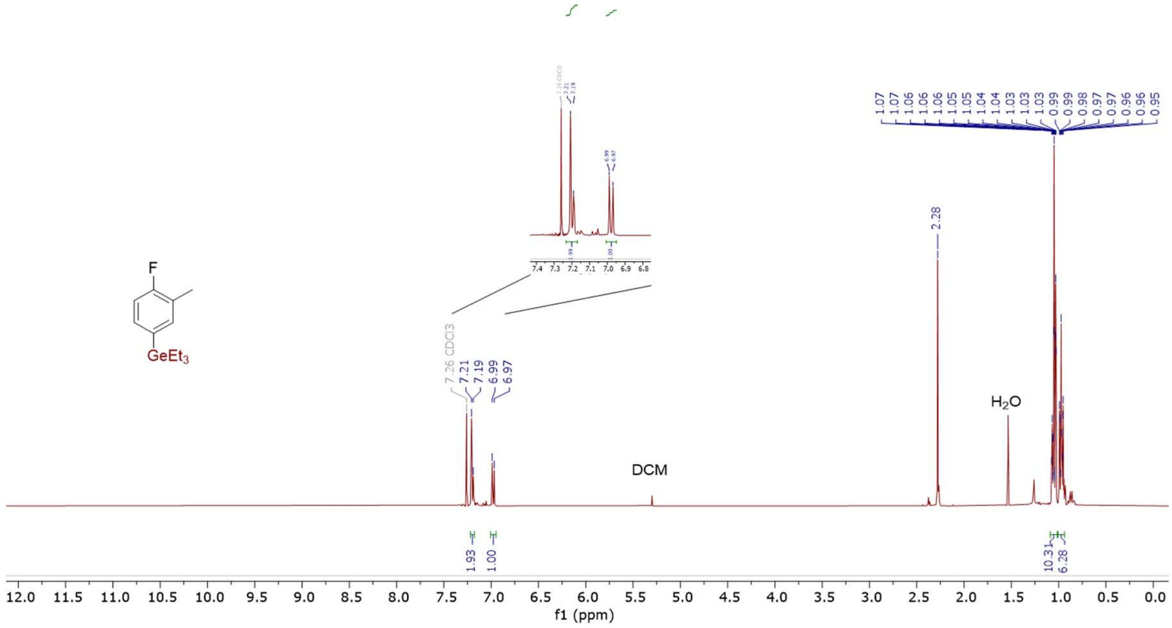

Figure S35: $^{1}H\{^{19}F\}$ NMR spectrum of compound **2j** in $CDCl_3$.

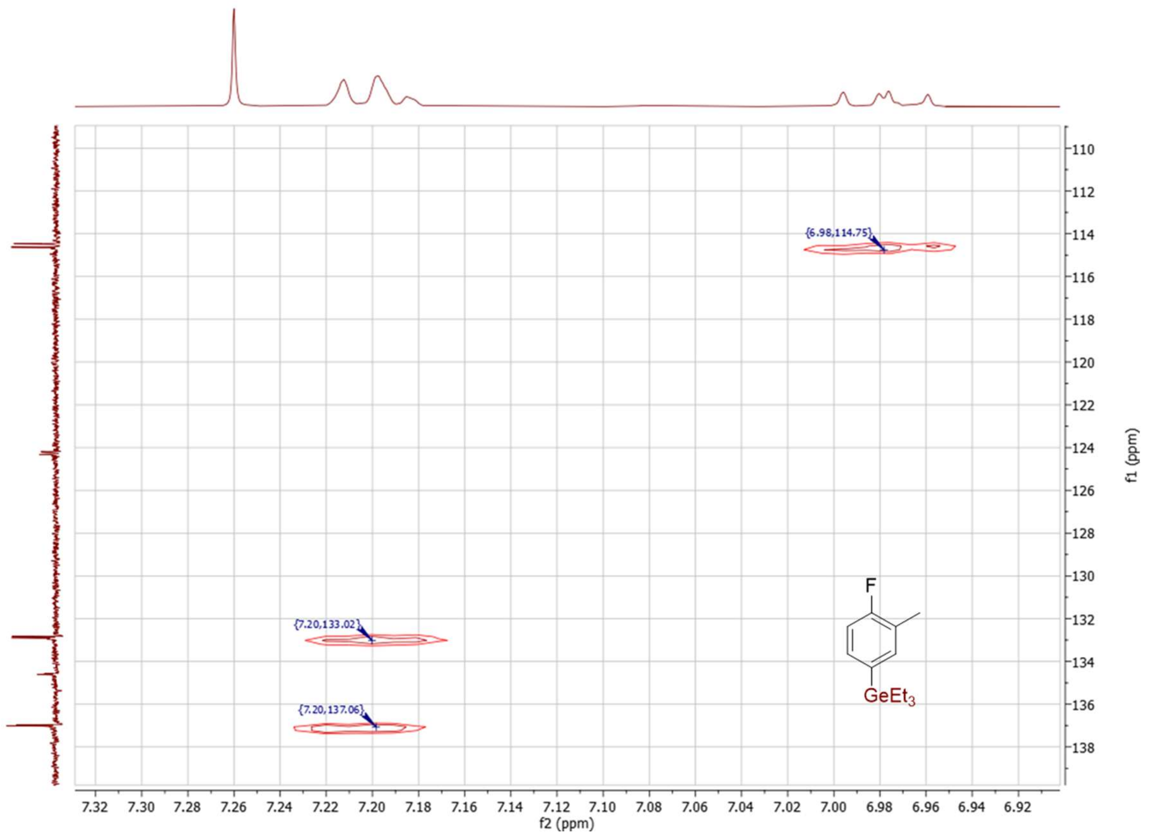

Figure S36: HSQC NMR spectrum of compound **2j** in $CDCl_3$.

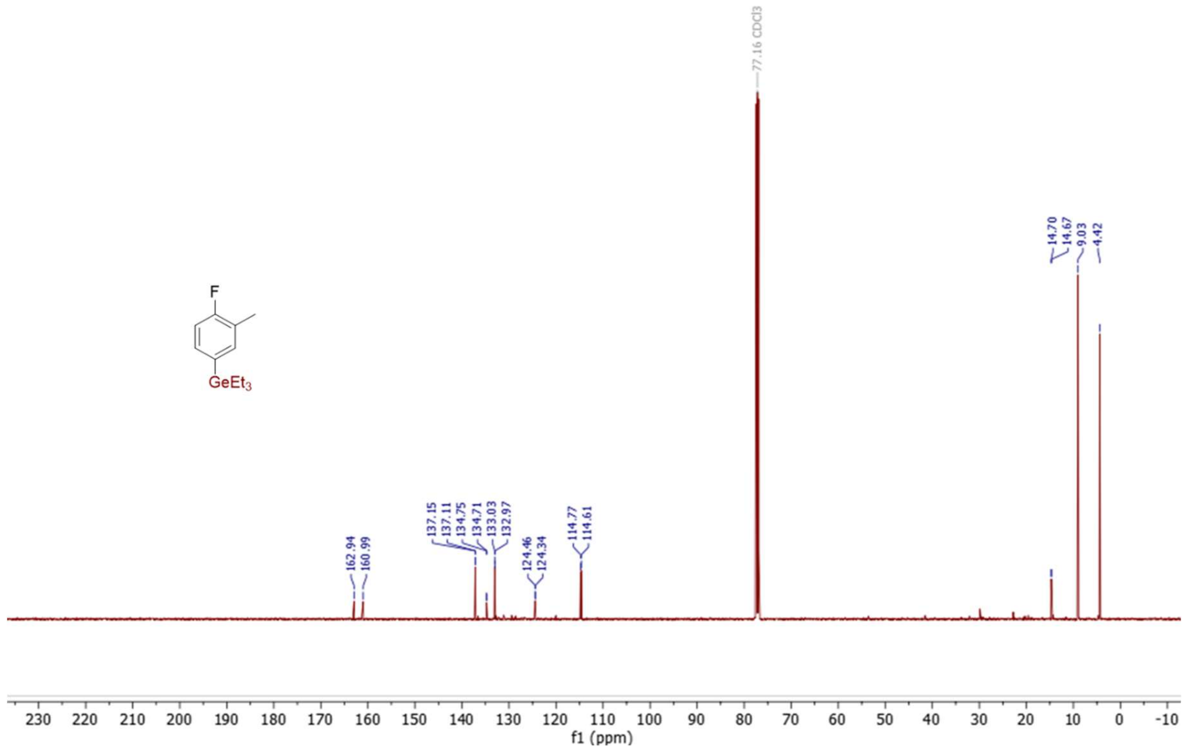

Figure S37: $^{13}C\{^{1}H\}$ NMR spectrum of compound **2j** in $CDCl_3$.

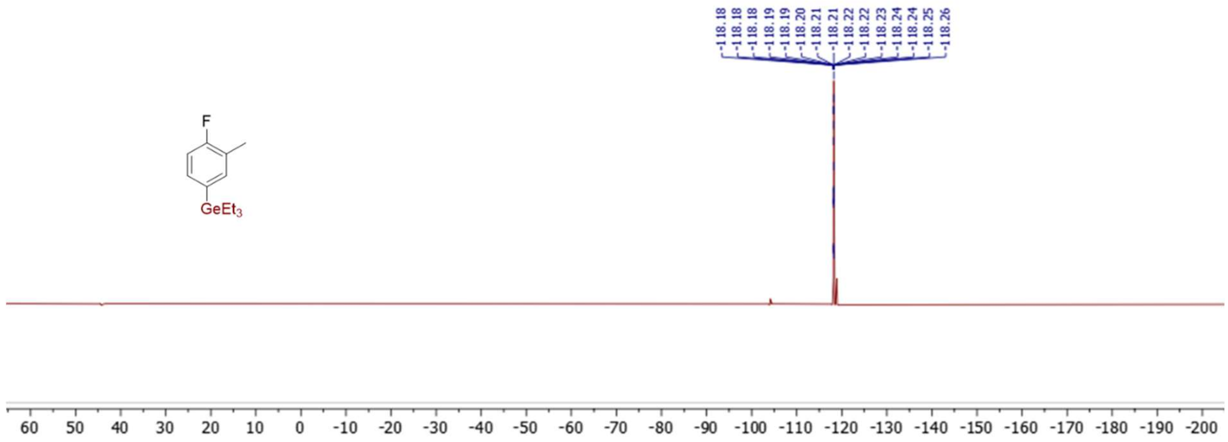

Figure S38: ^{19}F NMR spectrum of compound **2j** in $CDCl_3$.

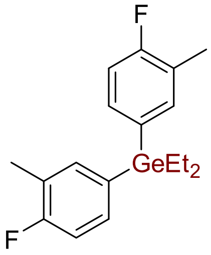

3j

Isolated as the major product (59% yield, 51 mg) from the C–H germylation of fluorotoluene (see earlier).

^{1}H NMR (400 MHz, $CDCl_3$): δ 7.24-7.19 (m, 4H, Ar*H*), 7.00 (dd, *J* = 10.4, 7.9 Hz, 2H, Ar*H*), 2.28 (d, *J* = 2.0 Hz, 6H, C*H*3), 1.25-1.20 (m, 4H, Ge*Et*3), 1.09 (t, *J* = 7.6 Hz, 6H Ge*Et*3).

^{1}H{^{19}F} NMR (400 MHz, $CDCl_3$): δ 7.23 (s, 2H, Ar*H*), 7.22 (d, *J* = 7.9 Hz, 2H, Ar*H*, overlapping with the singlet at 7.23), 7.01 (d, *J* = 7.9 Hz, 2H, Ar*H*), 2.28 (s, 6H, C*H*3), 1.25-1.20 (m, 4H, Ge*Et*3), 1.09 (t, *J* = 7.6 Hz, 6H Ge*Et*3).

^{13}C{^{1}H} NMR (126 MHz, $CDCl_3$): δ 162.2 (d, *J* = 245.9 Hz), 137.6 (d, *J* = 4.7 Hz), 133.6 (d, *J* = 7.4 Hz), 133.2 (d, *J* = 4.5 Hz), 124.7 (d, *J* = 15.9 Hz), 114.9 (d, *J* = 20.9 Hz), 14.7 (d, *J* = 4.0 Hz), 9.0, 5.3.

^{19}F NMR (471 MHz, $CDCl_3$): δ –117.3 - –117.4 (m).

Mass Spectrometry: Calculated [(M-Et)$^+$] = 321.05046, Observed [(M-Et)$^+$] = 321.05053.

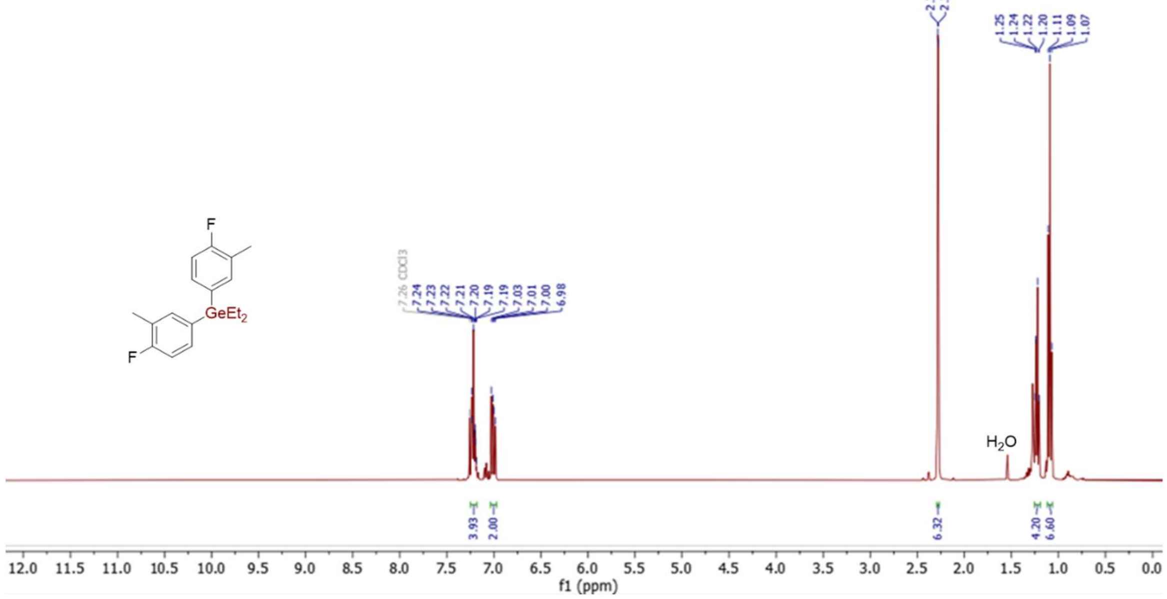

Figure S39: ^{1}H NMR spectrum of compound **3j** in $CDCl_3$.

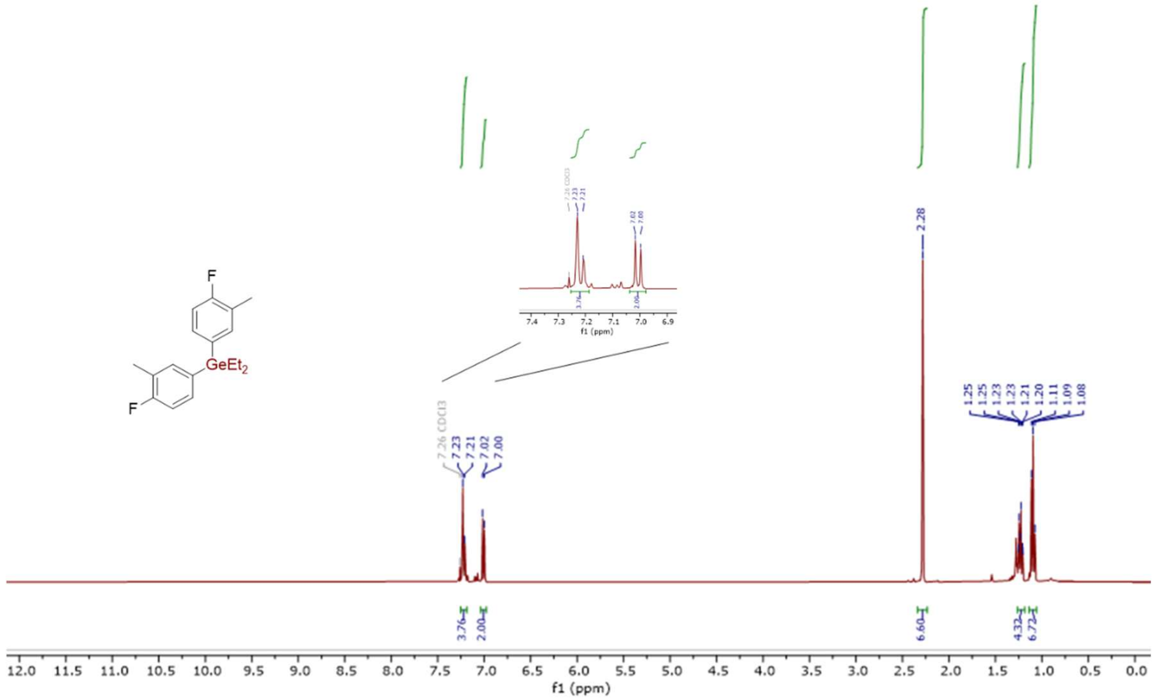

Figure S40: $^1H\{^{19}F\}$ NMR spectrum of compound **3j** in $CDCl_3$.

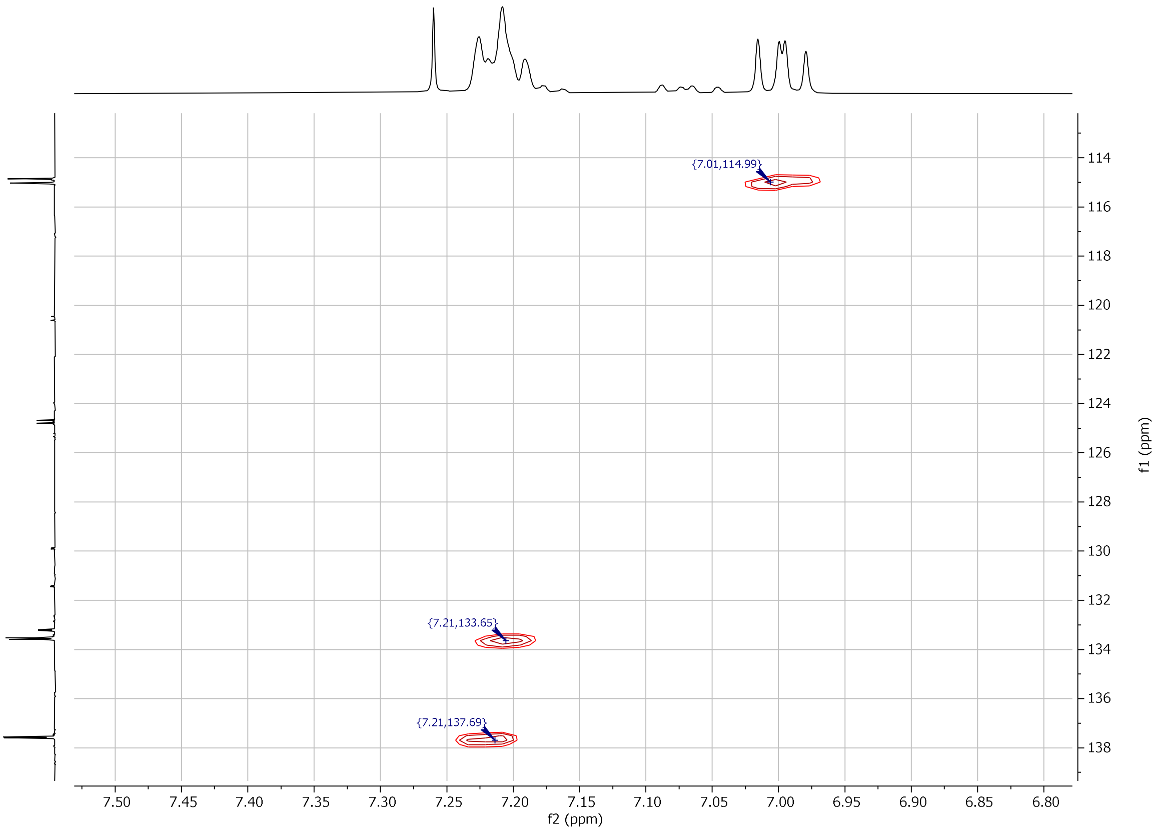

Figure S41: HSQC NMR spectrum of compound **3j** in $CDCl_3$.

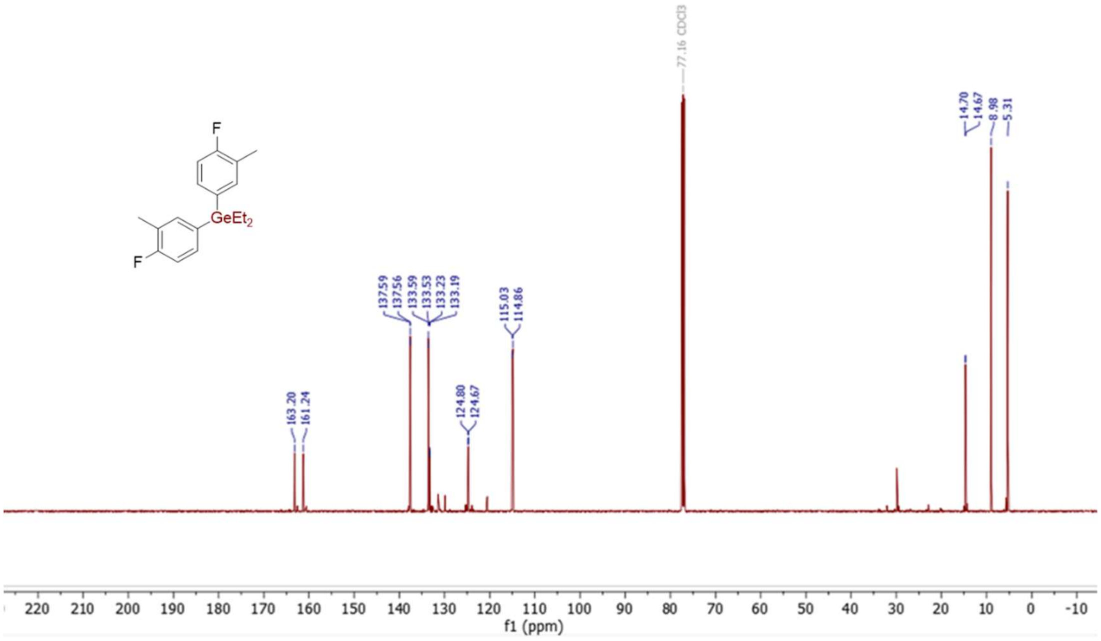

Figure S42: $^{13}C\{^1H\}$ NMR spectrum of compound **3j** in $CDCl_3$.

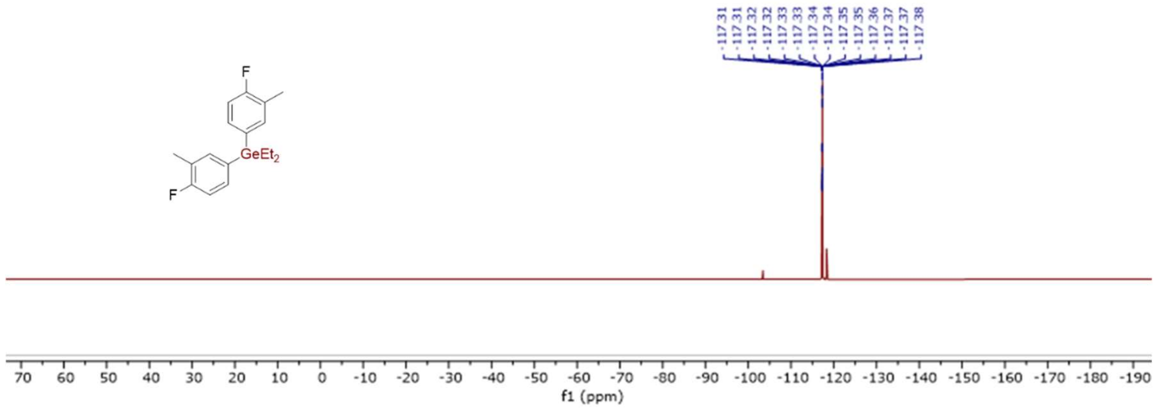

Figure S43: ^{19}F NMR spectrum of compound **3j** in $CDCl_3$.

S2.3. Unsuccessful substrates in C–H germylation.

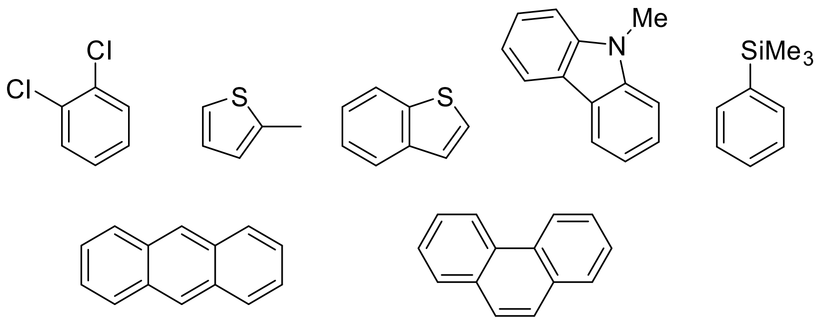

S3. Mechanistic investigation

S3.1. Attempted C−H germylation without $AlCl_3$.

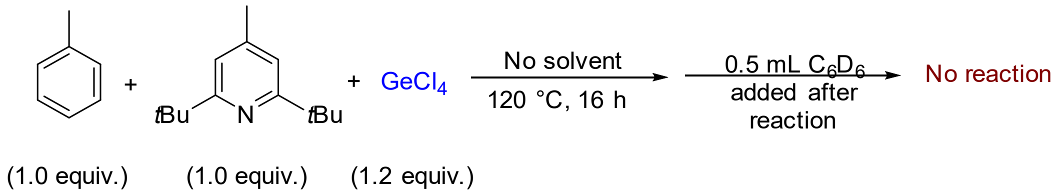

In a glovebox, 2,6-ditertbutyl-4-methyl-pyridine (103 mg, 0.5 mmol, 1.0 equiv.) was charged in a J. Young's NMR tube. Toluene (54 µL, 0.5 mmol, 1.0 equiv.) and $GeCl_4$ (70 µL, 0.6 mmol, 1.2 equiv.) were subsequently added at room temperature. The NMR tube was then sealed under argon and heated at 120 °C for 16 h. Upon completion, C_6D_6 (0.4 mL) was added to the reaction mixture. No C−H germylation of toluene was observed via NMR spectroscopy in the absence of $AlCl_3$.

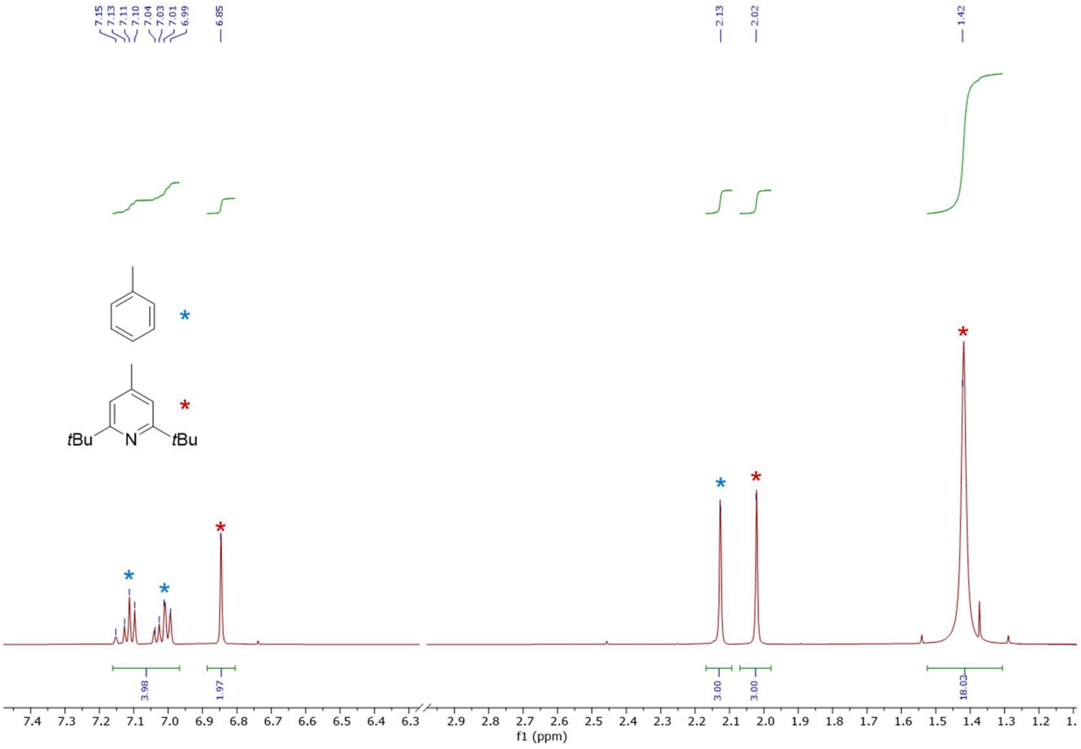

Figure S44: No C–H germylation of toluene in the absence of $AlCl_3$ was observed by ^1H NMR spectroscopy in C_6D_6.

S3.2. C−H germylation with a variable amount of $AlCl_3$.

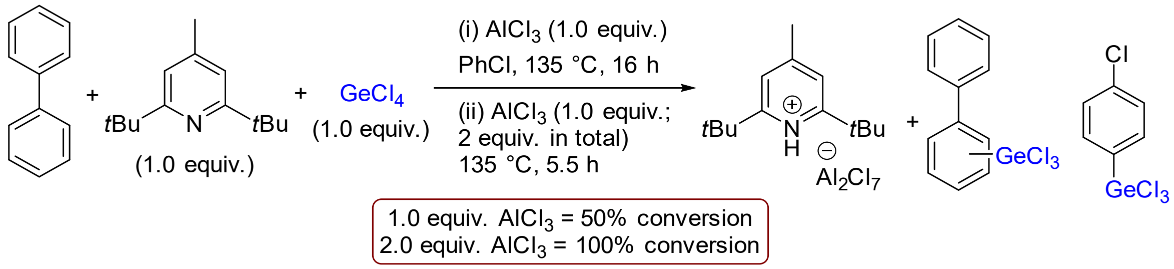

In a glovebox, biphenyl (15 mg, 0.1 mmol, 1.0 equiv.), $AlCl_3$ (13 mg, 0.1 mmol, 1.0 equiv.), and 2,6-ditertbutyl-4-methyl-pyridine (21 mg, 0.1 mmol, 1.0 equiv.) were charged in a J. Young's NMR tube followed by dissolution in chlorobenzene (0.6 mL) at room temperature. $GeCl_4$ (12 µL, 0.1 mmol, 1.0 equiv.) was subsequently added at room temperature. The NMR tube was then sealed under argon and heated at 135 °C for 16 h. Incomplete conversion (*ca* 50%) was determined via ^{1}H NMR spectroscopy. Next, in a glovebox extra $AlCl_3$ (13 mg, 0.1 mmol, 1.0 equiv., 2.0 equiv. in total) was added and the NMR tube was again sealed under argon and heated at 135 °C for 5.5 h. Full conversion (100%) was determined via ^{1}H NMR spectroscopy.

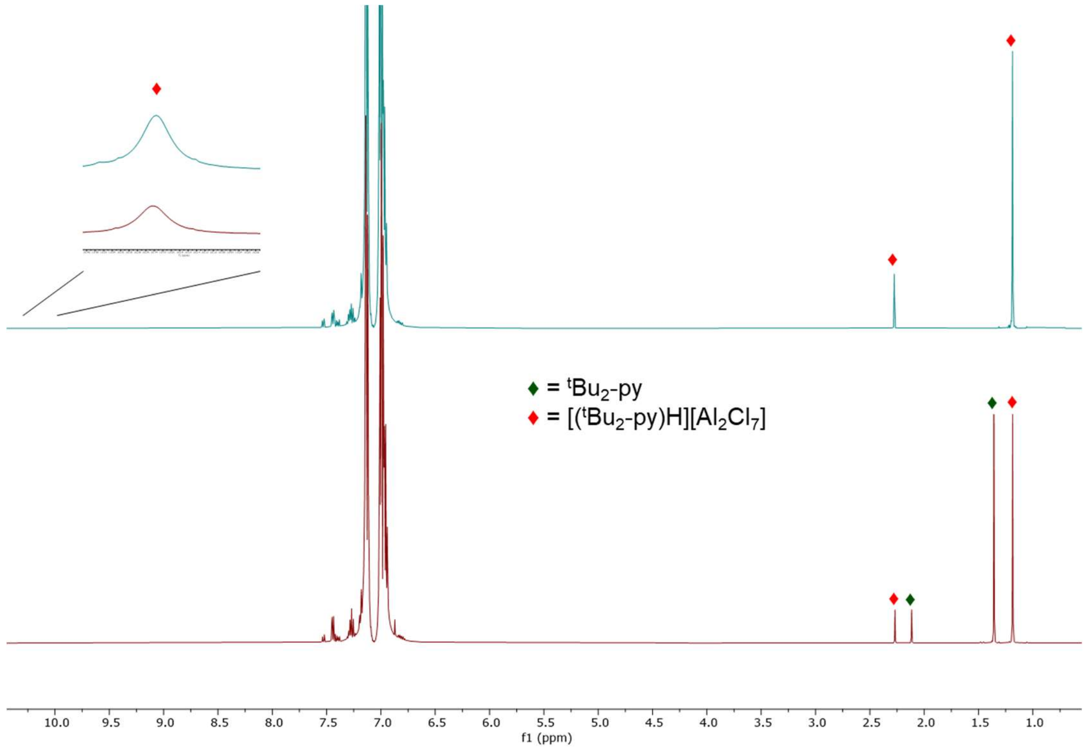

Figure S45: C–H germylation of biphenyl monitored by ^{1}H NMR spectroscopy in PhCl in the presence of: $AlCl_3$ (1 equiv.) and heating for 16 h at 135 °C (bottom), after addition of extra $AlCl_3$ (2 equiv. in total) and heating at 135 °C for 5.5 h (top).

S3.3. C(sp^3)−H alumination of 2,6-ditertbutyl-4-methyl pyridine in the absence of $GeCl_4$.

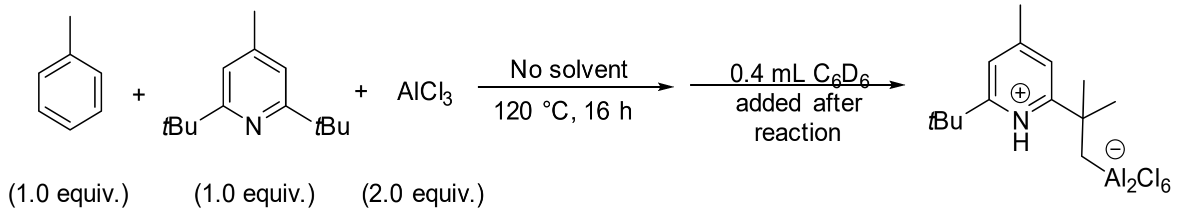

In a glovebox, $AlCl_3$ (134 mg, 1.0 mmol, 2.0 equiv.), and 2,6-ditertbutyl-4-methyl-pyridine (103 mg, 0.5 mmol, 1.0 equiv.) were charged in a J. Young's NMR tube. Toluene (54 µL, 0.5 mmol, 1.0 equiv.) was subsequently added at room temperature. The NMR tube was then sealed under argon and heated at 120 °C for 16 h. Upon completion, C_6D_6 (0.4 mL) was added to the reaction mixture. No sp^2 C−H alumination of toluene was not observed via NMR spectroscopy in the absence of $GeCl_4$. Instead sp^3 C−H alumination of the 2,6-ditertbutyl-4-methyl-pyridine forming **A** was observed.[7]

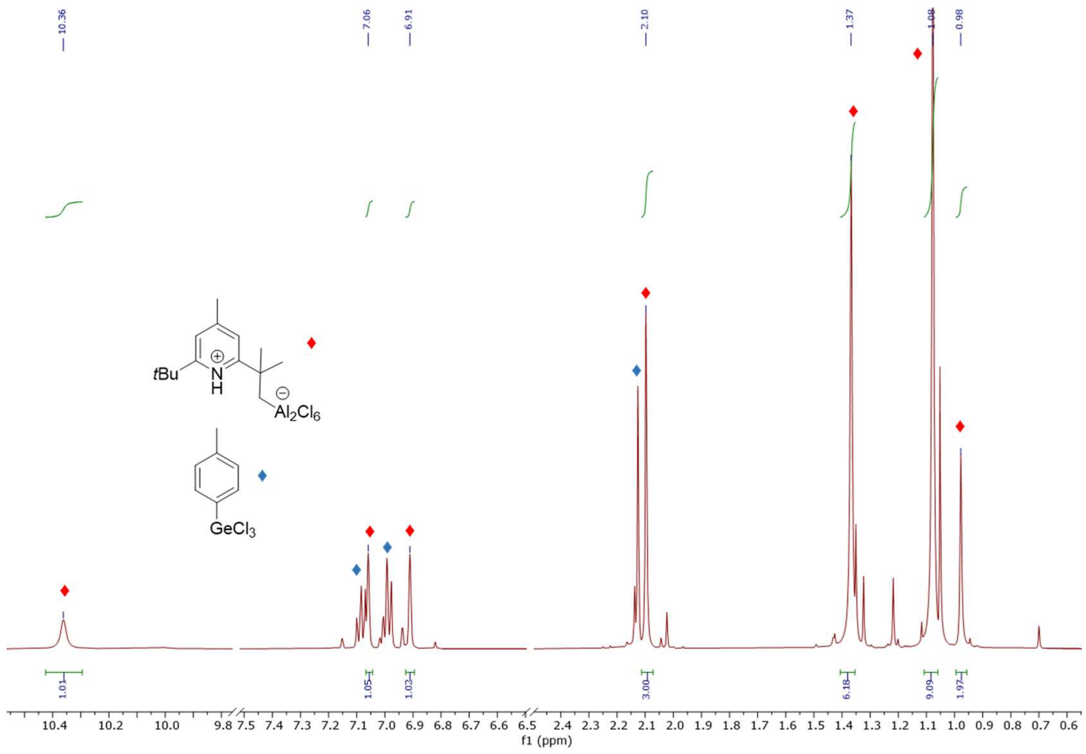

Figure S46: C(sp^3)–H alumination of 2,6-ditertbutyl-4-methyl-pyridine observed by ^{1}H NMR spectroscopy in C_6D_6.

S3.4. Synthesis of the sp^3 C−H alumination product (**A**) of 2,6-ditertbutyl-4-methyl pyridine and subsequent reaction with toluene and $GeCl_4$.

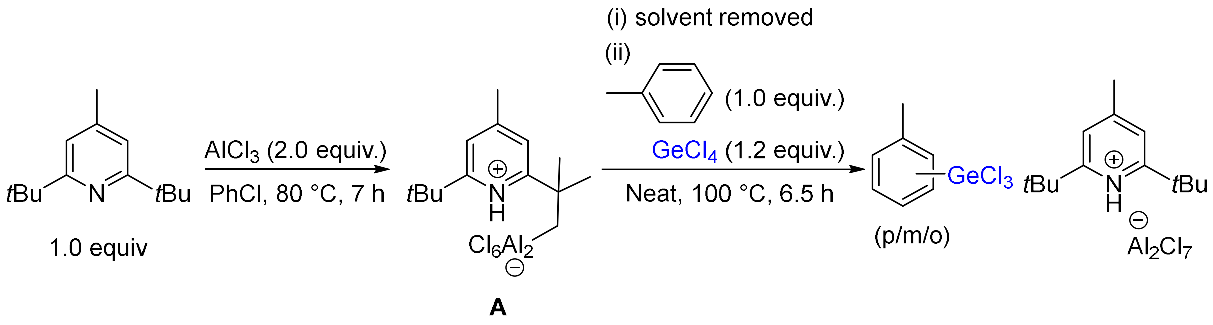

In a glovebox, $AlCl_3$ (134 mg, 1.0 mmol, 2.0 equiv.) and 2,6-ditertbutyl-4-methyl-pyridine (103 mg, 0.5 mmol, 1.0 equiv.) were charged in a J. Young's NMR tube followed by the addition of chlorobenzene (0.6 mL) at room temperature. The NMR tube was then sealed under argon and heated at 80 °C for 7 h. Formation of the sp^3 C−H alumination product (**A**) was confirmed via NMR spectroscopy which was in agreement with the NMR data reported previously in the literature.[7] Subsequently, the solvent was removed under vacuum and toluene (54 µL, 0.5 mmol, 1.0 equiv.) and $GeCl_4$ (70 µL, 0.6 mmol, 1.2 equiv.) were added at room temperature. The NMR tube was then sealed under argon and heated at 100 °C for 6.5 h and upon completion, C_6D_6 (0.4 mL) was added. The conversion to the products was determined by the integration of diagnostic ^{1}H (*Me*-$C_6H_4GeCl_3$) resonances. Formation of the sp^2 C−H germylation product of toluene in 85% yield (p/m/o = 47/28/10) and 15% unreacted **A** was confirmed via NMR spectroscopy.

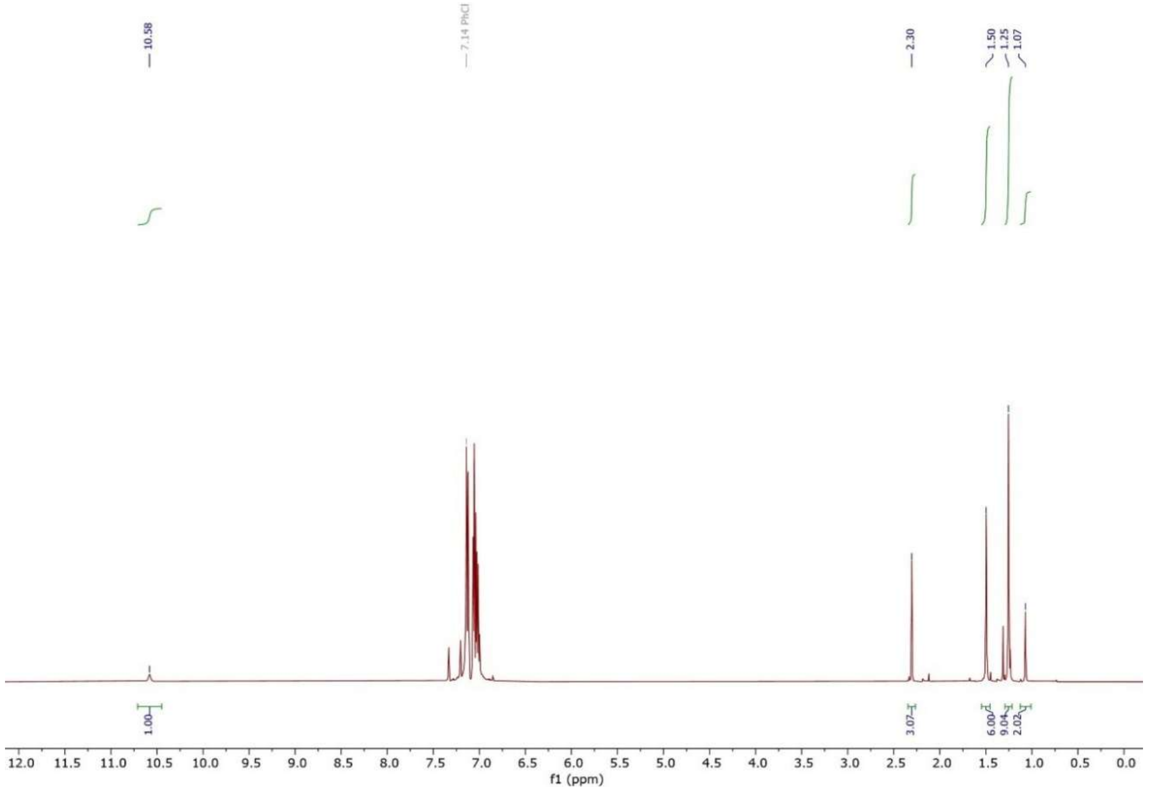

Figure S47: $C(sp^3)$–H alumination of 2,6-ditertbutyl-4-methyl-pyridine observed by ^1H NMR spectroscopy in PhCl.

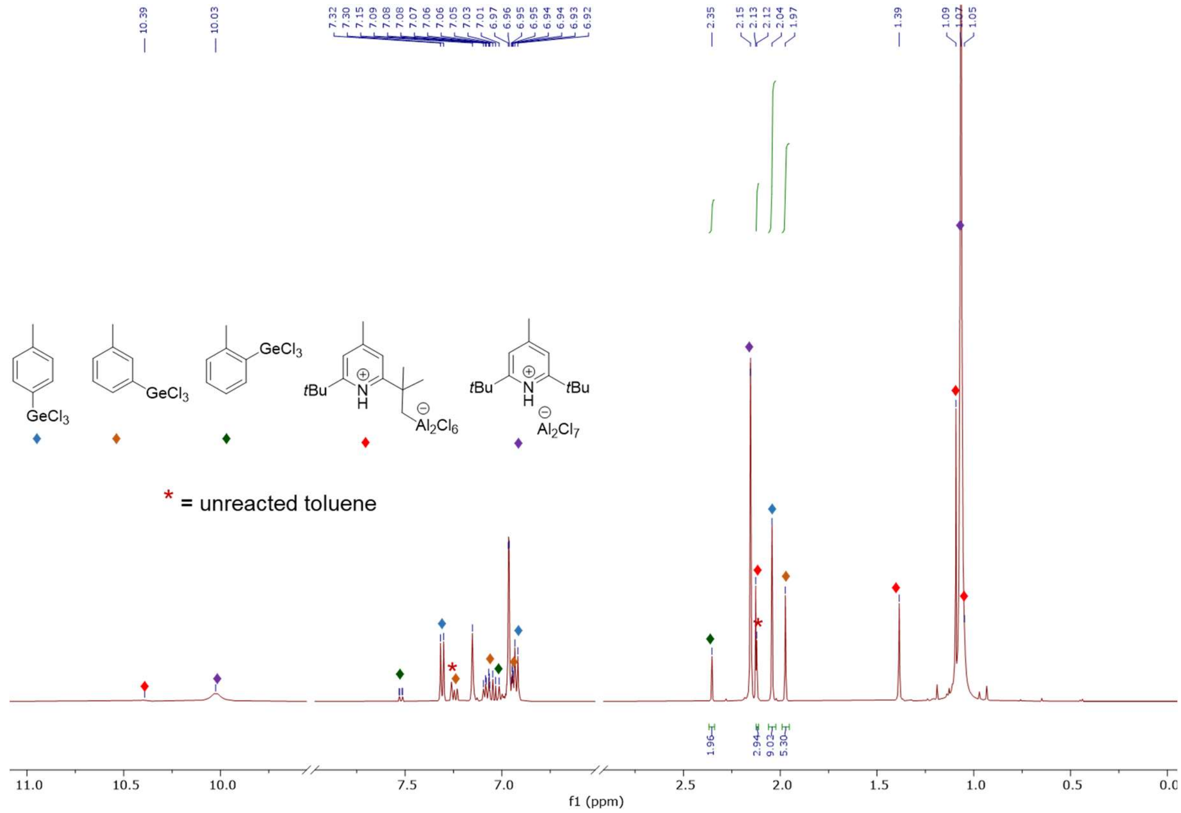

Figure S48: Transformation of $C(sp^3)$–H alumination product of 2,6-ditertbutyl-4-methyl-pyridine to $C(sp^2)$–H germylation of toluene observed by ^1H NMR spectroscopy in C_6D_6.

S3.5. Determination of experimental kinetic isotope effect (KIE) for C–H germylation (all experiments run in duplicates).

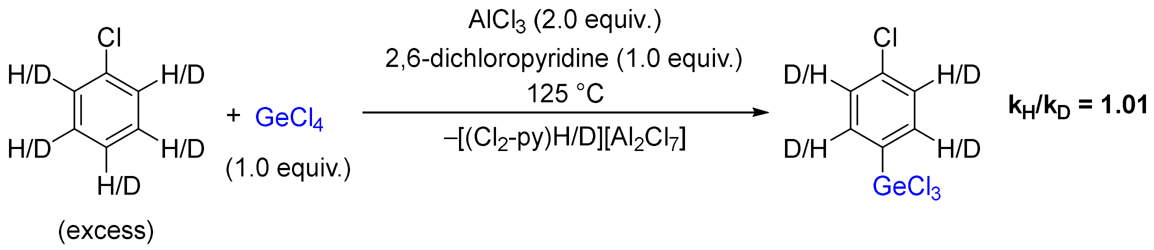

In a glovebox, $AlCl_3$ (59 mg, 0.4 mmol, 2.0 equiv.), and 2,6-dichloropyridine (33 mg, 0.2 mmol, 1.0 equiv.) were charged in a J. Young's NMR tube. Subsequently, chlorobenzene or 5-*d5*-chlorobenzene (0.6 mL, 5.9 mmol, excess), $GeCl_4$ (25 µL, 0.2 mmol, 1.0 equiv.) and cyclohexane (23.5 µL, 0.2 mmol, 1.0 equiv.), acting as an internal standard, were added to the reaction mixture at room temperature. The J. Young's NMR tube was then sealed under argon and heated at 125 °C. A homogenous reaction mixture was obtained within 30 minutes of heating. Reaction progress was monitored via ^{1}H NMR spectroscopy in 1 h time intervals up to 6 h. Conversion to the product was determined by the integration of the diagnostic ^{1}H NMR resonances of the protonated base formed stoichiometrically, $[Cl_2\text{-pyH/D}][Al_2Cl_7]$ (the N–H peak in the reaction with chlorobenzene and the para-C–H in the reaction with *d5*-chlorobenzene) against the internal standard.

With the average taken from two different runs for each experiment, concentration of the product $[Cl_2\text{-pyH}][Al_2Cl_7]$ (with chlorobenzene) or $[Cl_2\text{-pyD}][Al_2Cl_7]$ (with *d5*-chlorobenzene) versus time (h) was plotted and the KIE calculated to be 1.01 [equation (1)].

Time (h)	$[Cl_2\text{-pyH}][Al_2Cl_7]$ (mM)	$[Cl_2\text{-pyD}][Al_2Cl_7]$ (mM)
2	0.2166[a]	0.1444[a]
3	0.2648	0.1874
4	0.2992	0.2269
5	0.3249	0.2441
6	0.3421	0.2699

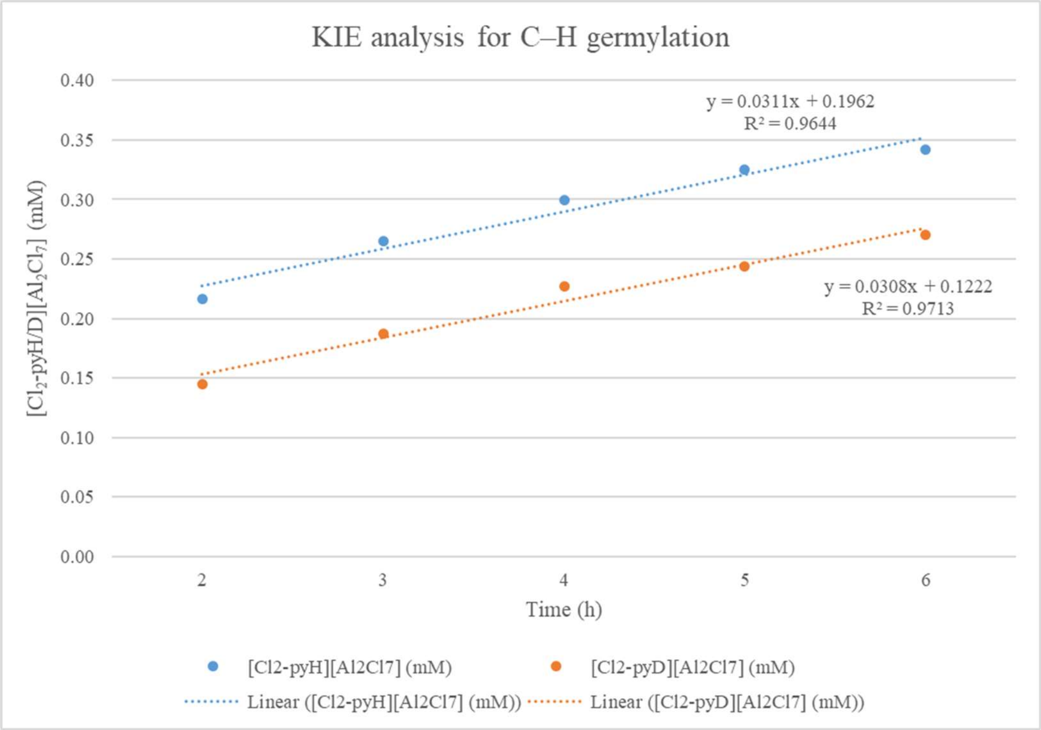

Figure S49: Concentration of $[Cl_2\text{-pyH}][Al_2Cl_7]$ vs $[Cl_2\text{-pyD}][Al_2Cl_7]$ as a function of time for the C–H germylation. [a] The difference in the initial concentration of the formed product is tentatively attributed to a variable water content of the starting material and reagents' solubility in the solvent at ambient temperature.

$$\frac{k_H}{k_D} = \frac{0.0311}{0.0308} = 1.01 \tag{1}$$

S4. Computational details

DFT calculations were run with Gaussian 16 (Revision A.03).[8] Geometry optimizations and thermodynamic corrections were performed with the PBE0 functional[9] with def2-SVP basis sets[10,11] for all atoms. All stationary points were fully characterized via analytical frequency calculations as either minima (all positive frequencies) or transition states (one negative frequency) and the latter were characterized via IRC calculations and subsequent geometry optimizations to confirm the adjacent minima. Electronic energies were recomputed with the B3PW91 functional[12] using def2-TZVP basis sets (BS2),[10,11] a correction for dispersion (BJD3)[13] and chlorobenzene solvent (SMD approach).[14] The thermochemical corrections from the PBE0 frequency calculations were then added to give the free energies quoted in the text.

A solvent polarity study was performed via recomputing the electronic energy with the B3PW91 functional using def2-TZVP basis sets (BS2), a correction for dispersion (BJD3) and variable solvents (SMD approach). Additional functional testing was performed with the BP86,[15,16] BLYP,[15,17] B3LYP,[18] PBE,[19] B97D3,[18] B97D,[18] M05,[20] M05-2x,[21] M06,[22] M06-2x[22], MN15,[23] ωB97x-D[24] and TPSS[25] functionals. Electronic energies were recomputed with each functional using def2-TZVP basis sets (BS2), a correction for dispersion (BJD3, for BP86, BLYP, B3LYP, PBE and TPSS) and chlorobenzene solvent (SMD approach). The thermochemical corrections from the PBE0 frequency calculations were then added to give the free energies. Details of the solvent polarity study, functional testing and all computed structures are provided below, the latter also as a separate XYZ file.

S4.1. Functional testing.

Functional testing for the transition states in the $AlCl_3$-mediated C–H germylation of benzene using $GeCl_4$ and 2,6-dichloropyridine was performed (Table S2). For the majority of the screened functionals, **TS2** was consistently calculated to be higher in energy than **TS3**. An opposite trend was observed with all Minnesota functionals tested which predict a significant primary KIE should be observed (**TS3** being higher in energy than **TS2**). In order to be consistent with the experimental data, all further calculations were performed with the B3PW91 functional – this protocol was chosen based on its good fit with the experimental KIE and its performance in our previous studies on electrophilic C–H zincation.[26]

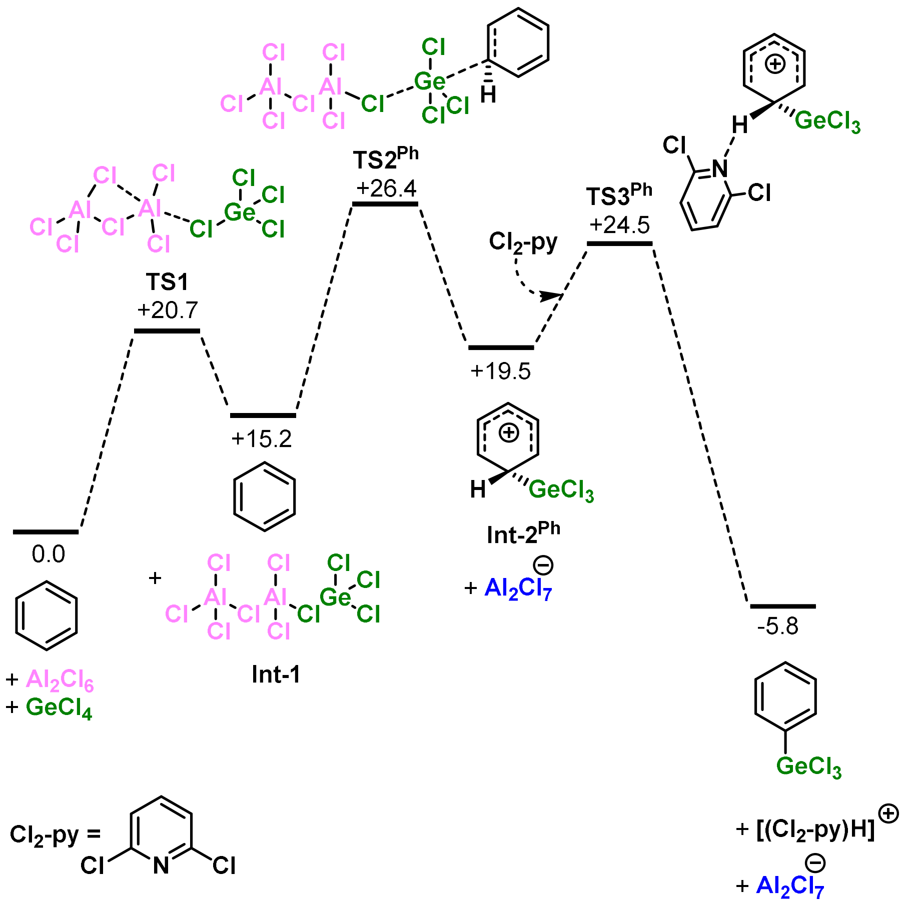

Figure S50: Computed free energy profile (kcal/mol) for the $AlCl_3$-mediated C–H germylation of benzene using $GeCl_4$ and 2,6-dichloropyridine at 25°C. [Method: B3PW91(Def2-TZVPP, BJD3, PhCl)//PBE0(Def2-SVP, BJD3)].

Table S2: Functional testing for the transition states in the $AlCl_3$-mediated C–H germylation of benzene using $GeCl_4$ and 2,6-dichloropyridine. Free energies quoted in the table (kcal/mol) were calculated at room temperature.

Species	BP86	BLYP	B3LYP	PBE	PBE0	B97D3	B97D
**$TS1^{Ph}$**	18.4	21.3	22.7	22.8	23.6	23.3	27.8
**$TS2^{Ph}$**	23.6	26.9	28.4	32.2	31.8	29.4	34.3
**$TS3^{Ph}$**	20.8	24.1	26.5	26.4	28.2	25.2	27.4

Species	WB97xD	TPSS	B3PW91	M05-2X	M06	M06-2X	MN15
**$TS1^{Ph}$**	27.6	21.3	20.7	25.5	24.8	24.1	22.0
**$TS2^{Ph}$**	36.1	29.8	26.4	34.2	31.9	32.2	29.9
**$TS3^{Ph}$**	31.5	27.1	24.5	34.9	32.4	33.6	31.9

S4.2. Solvent polarity study.

The substrate scope for C–H germylation was performed under neat conditions (*no solvent* was used). However, during the neat reactions various compounds are or become (due to the temperature of the reaction) liquids including: Cl_2-Py, $GeCl_4$ and in most cases the arene substrate. Furthermore, as the reaction proceeds $ArGeCl_3$ is produced (many of which are liquids even at 20°C) along with [Cl_2-Py][Al_2Cl_7] (related [pyridinium][Al_2Cl_7] salts exist as ionic liquids). Due to this complexity, we were unable to accurately represent this mixed-solvent system. However, a screen varying the dielectric constant (**ε**) of the solvent was performed to gain insight into how the polarity of the solvent impacts the transition states (Table S3). It can be noted that as the solvent polarity increases (**ε** increases) TS1 remains effectively unaffected, however both TS2 and TS3 decrease, albeit the latter more rapidly.

In order to remain consistent throughout the DFT calculations (*i.e.* KIE experiments which were performed in PhCl, *vide infra*), the reaction profile calculated for benzene (Figure S50) employed a chlorobenzene solvation model (SMD approach). However, due to the presence of more polar media in the reactions the free energies calculated may be overestimated.

Table S3: Solvent polarity study varying the dielectric constant (ε) in the calculation of the transition states in the $AlCl_3$-mediated C–H germylation of benzene using $GeCl_4$ and 2,6-dichloropyridine. Free energies quoted in the table (kcal/mol) were calculated at room temperature.

Solvent	MeOH	Acetone	m-Cresol	PhCl	CCl_4
**ε**	32.613	20.493	12.440	5.697	2.228
**$TS1^{Ph}$**	20.7	20.7	20.7	20.7	20.8
**$TS2^{Ph}$**	21.9	22.8	23.1	26.4	32.1
**$TS3^{Ph}$**	14.4	15.6	17.7	24.5	43.7

S4.3. Calculation of theoretical KIE for C–H germylation.

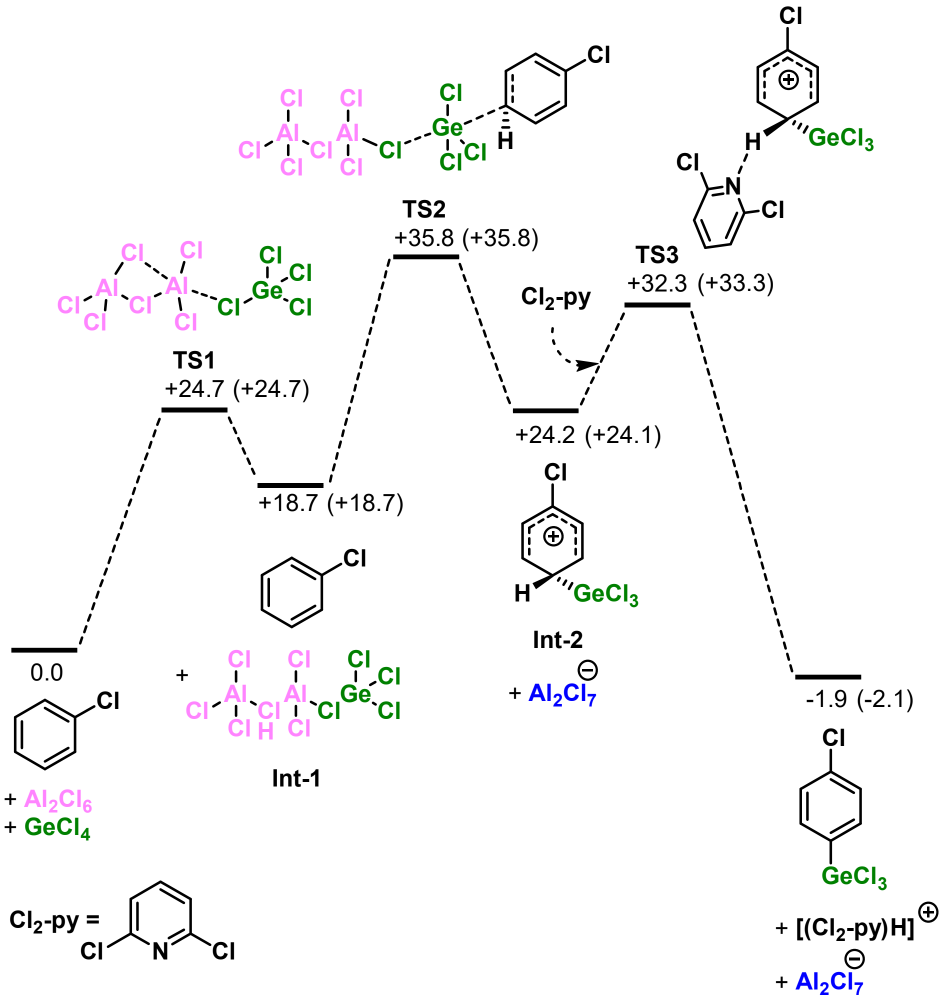

Figure S51: Computed free energy profile (kcal/mol) for the $AlCl_3$-mediated C–H germylation of chlorobenzene using $GeCl_4$ and 2,6-dichloropyridine at 125°C. [Method: B3PW91(Def2-TZVPP, BJD3, PhCl)//PBE0(Def2-SVP, BJD3)]. Selected Gibbs free energies (kcal/mol) computed for d_5-chlorobenzene at 125°C are provided in parenthesis.

Based on the free energy profile computed for the C–H germylation of chlorobenzene or d_5-chlorobenzene using $GeCl_4$ and 2,6-dichloropyridine at 125°C (see Figure S51) the relevant energy barriers ($\Delta G^{\ddagger}$) were extracted and respective KIEs were calculated [equation (2)].

$$\frac{k_H}{k_D} = e^{-(\Delta G_H^{\ddagger} - \Delta G_D^{\ddagger})/RT} \tag{2}$$

Table S4: Energy barriers (kcal/mol) and associated KIEs calculated at 125°C for selected transition states in C–H germylation of chlorobenzene or d_5-chlorobenzene.

RDS	ΔG‡ (kcal/mol) at 125°C	k_H/k_D at 125°C
TS2	35.8	**1.00**
d-TS2	35.8	
TS3	32.3	**3.54**
d-TS3	33.3	

S4.4. Additional energy profiles.

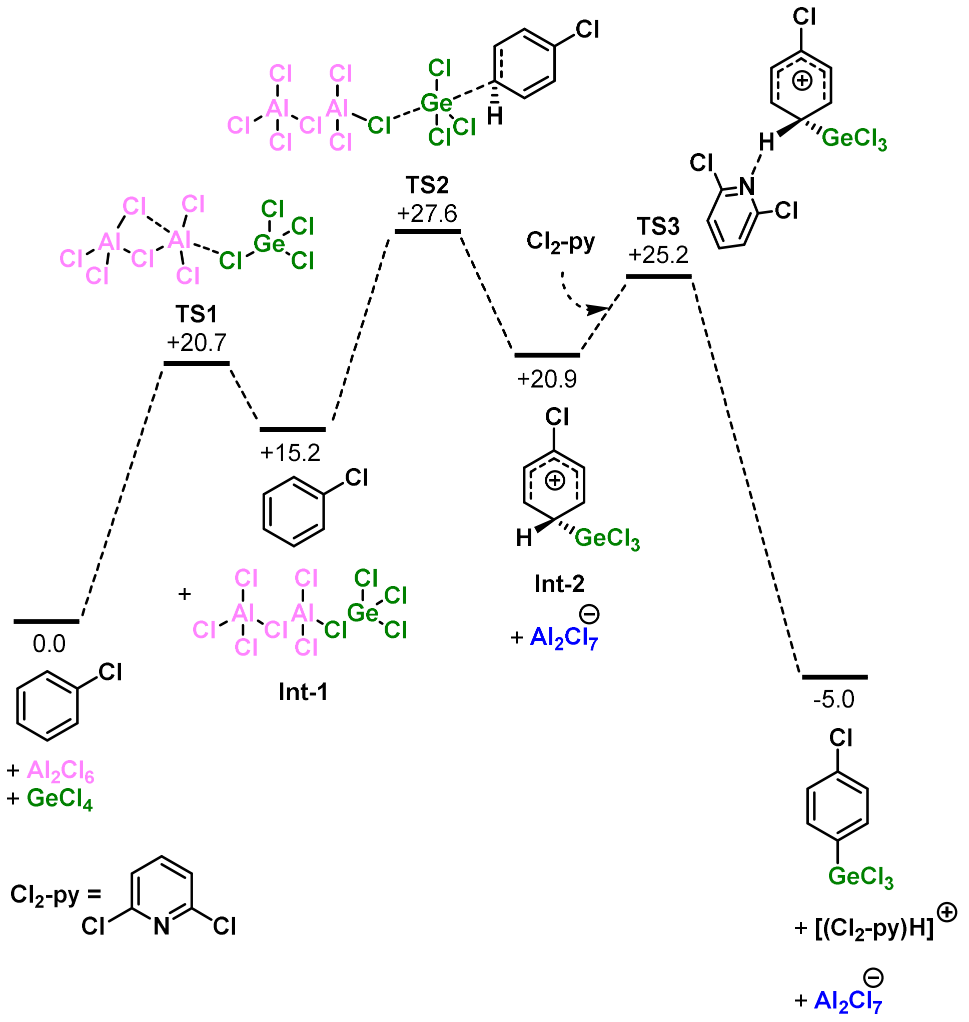

Figure S52: Computed free energy profile (kcal/mol) for the $AlCl_3$-mediated C–H germylation of chlorobenzene using $GeCl_4$ and 2,6-dichloropyridine at 25°C. [Method: B3PW91(Def2-TZVPP, BJD3, PhCl)//PBE0(Def2-SVP, BJD3)].

An alternative mechanism exploring the use of Cl_3Ge-(μ-Cl)-$AlCl_3$ (which was found to be a minimum) as the germylating agent in an S_EAr-type process was considered (**Figure S53**). However, all attempts to progress forward along the reaction coordinate by interacting benzene with the germanium centre led to scans that only increased in energy and no transition state could be located.

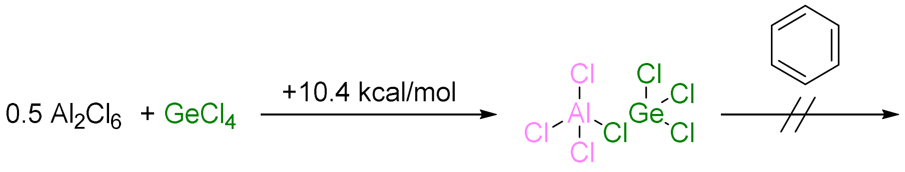

Int-1(AlCl$_3$)

Figure S53: Computed free energy (kcal/mol) for the formation of Cl_3Ge-(μ-Cl)-$AlCl_3$ using $GeCl_4$ and Al_2Cl_6 (0.5 eq.) at 25°C. [Method: B3PW91(Def2-TZVPP, BJD3, PhCl)//PBE0(Def2-SVP, BJD3)].

Note, to select a zero energy starting point we considered possible products from combining Al_2Cl_6, $GeCl_4$ and Cl_2-Py (see below). At 125°C the lowest energy product from combining 1 eq. Cl_2-Py and 1 eq. Al_2Cl_6 is the Lewis adduct (Cl_2-Py)$AlCl_3$ and 0.5. Al_2Cl_6 (top).

ΔG(398)

Al_2Cl_6 + Cl_2-Py → (**-3.4**) Cl_3Al-(Cl_2-Py) + 0.5. Al_2Cl_6

Al_2Cl_6 + Cl_2-Py → (**-1.7**) Cl_6Al_2-(Cl_2-Py)

2 Al_2Cl_6+ 2 (Cl_2-Py) → (**0.0**) $[(Cl_2\text{-}Py)_2AlCl_2]^+$ + $[AlCl_4]^-$ + Al_2Cl_6 → (**-3.5**) $[(Cl_2\text{-}Py)_2AlCl_2]^+$ + $[Al_2Cl_7]^-$ + 0.5 Al_2Cl_6

Figure S54: Computed free energy (kcal/mol) for the formation of possible products from combining Cl_2-Py and Al_2Cl_6 at 125°C. [Method: B3PW91(Def2-TZVPP, BJD3, PhCl)//PBE0(Def2-SVP, BJD3)].

As discussed in the manuscript, entropic contributions calculated in the gas-phase are thought to be over-estimated compared to the solution phase and so the difference between the calculated TSs and the absolute barriers for the pathway in Figure 3 are likely to be exaggerated. One approach to address this has been to include only 50% of the gas-phase entropy in the calculation of the solution-phase free energy (denoted G_{50}).[27, 28] Applying this approach gives G50 values which are more feasible for a reaction proceeding at 125°C.

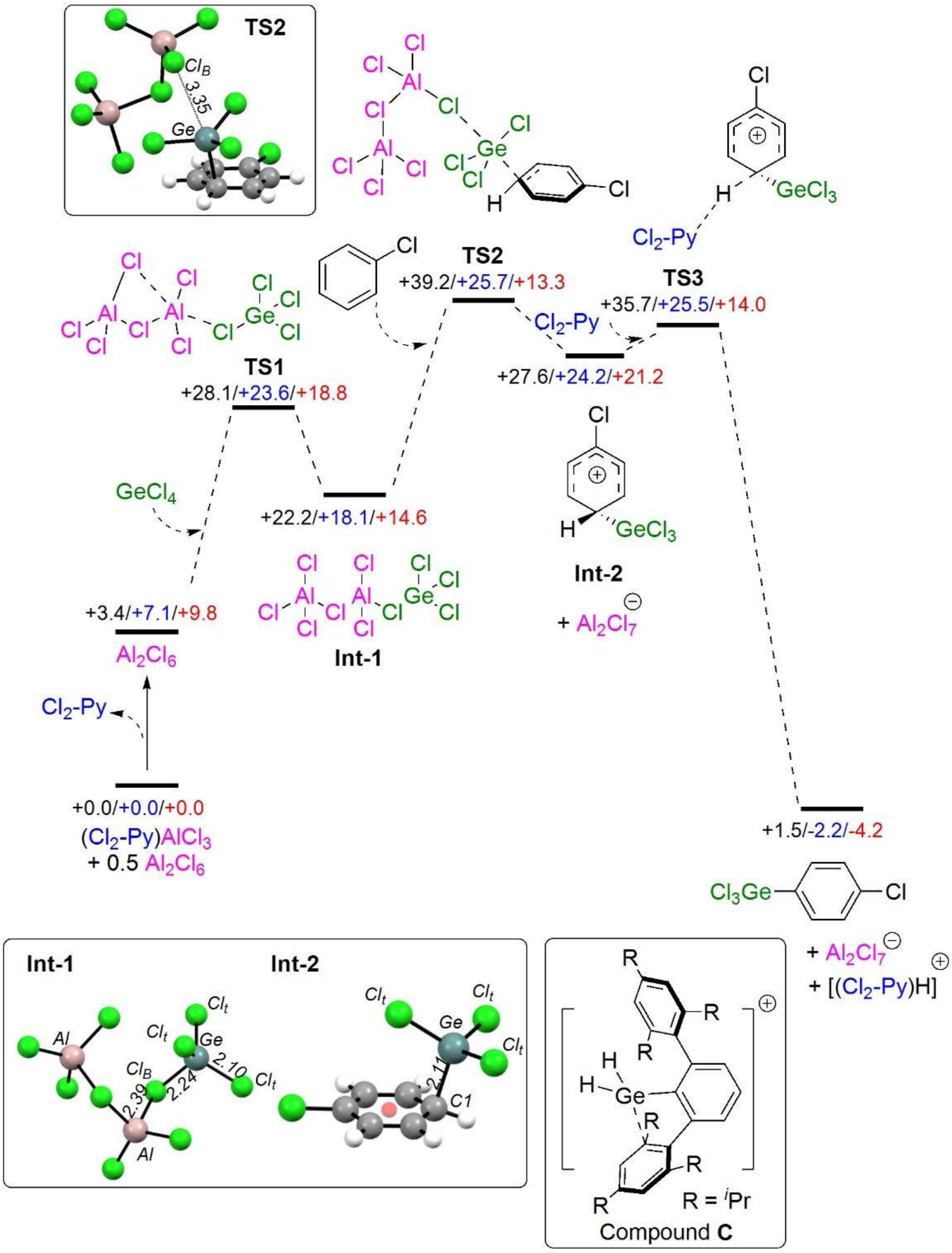

Figure S55: Computed free energy profile (kcal/mol) for the $AlCl_3$-mediated C–H germylation of chlorobenzene using $GeCl_4$ and 2,6-dichloropyridine at 125°C comparing data computed with the $\Delta G_{50\%}$ approach (in blue) with the original free energies (black) and enthalpies (red) reported in the main text. [Method: B3PW91(Def2-TZVPP, BJD3, PhCl)//PBE0(Def2-SVP, BJD3)].

S4.5. Computed Cartesian coordinates (Å) and energies (hartrees) for all species.

C_6H_5Cl
PBE0
SCF = -691.121632945
H (0 K) = -691.029846
H (398 K) = -691.019274
G (298 K) = -691.059581
G (398 K) = -691.072281
Low freq. = 191.8294
Second freq. = 301.1798
B3PW91
SCF (PhCl,D3BJ,BS2) = -691.851080282

12
C_6H_5Cl
C 1.56628 1.20556 0.00002
C 0.17288 1.21364 0.00000
C -0.51349 -0.00001 -0.00002
C 0.17289 -1.21364 -0.00000
C 1.56629 -1.20555 0.00000
C 2.26668 0.00000 0.00001
H -0.38592 2.15081 0.00001
Cl -2.24651 -0.00000 -0.00001
H -0.38589 -2.15083 -0.00001
H 2.10732 -2.15471 0.00000
H 3.35877 0.00002 0.00001
H 2.10732 2.15471 0.00003

C_6H_6
PBE0
SCF = -231.811413157
H (0 K) = -231.710119
G (298 K) = -231.737574
G (398 K) = -231.749061
Low freq. = 415.6313
Second freq. = 415.6693
B3PW91
SCF (C6H5Cl,D3BJ,BS2) = -232.271973508

12
C_6H_6
C -1.29827 0.50928 0.00000
C -0.20798 1.37895 -0.00011
C 1.09017 0.86969 0.00008
C 1.29823 -0.50937 -0.00002
C 0.20809 -1.37894 -0.00008
C -1.09023 -0.86961 0.00008
H -2.31520 0.90832 0.00007
H -0.37109 2.45914 -0.00006
H 1.94425 1.55080 0.00018
H 2.31525 -0.90816 0.00001
H 0.37094 -2.45915 -0.00003
H -1.94416 -1.55093 0.00007

$C_6H_5CH_3$
PBE0
SCF = -271.055506121
H (0 K) = -270.926977
G (298 K) = -270.958288
Low freq. = 17.3141
Second freq. = 211.3624
B3PW91
SCF (PhCl,D3BJ,BS2) = -271.595672128

15
$C_6H_5CH_3$
C 1.90145 -0.00144 0.00816
C 1.19984 1.20267 0.00202
C -0.19386 1.20144 -0.00874
C -0.91517 0.00183 -0.01158
C -0.19642 -1.20000 -0.00876
C 1.19667 -1.20432 0.00204
H 1.74141 2.15197 0.00196
H -0.73576 2.15151 -0.01760
C -2.41662 0.00095 0.00883
H -0.73993 -2.14927 -0.01765
H 1.73601 -2.15491 0.00196
H 2.99400 -0.00274 0.01418
H -2.82746 -0.83409 -0.57778
H -2.79710 -0.10687 1.03843
H -2.82654 0.93768 -0.39533

Cl_2-py
PBE0
SCF = -1166.44404409
H (0 K) = -1166.373728
H (398 K) = -1166.361778
G (298 K) = -1166.405594
G (398 K) = -1166.419403
Low freq. = 174.2964
Second freq. = 181.6285
B3PW91
SCF (PhCl,D3BJ,BS2) = -1167.46645127

11
Cl_2-py
C 1.12978 0.00425 0.00000
C 1.20423 -1.39009 0.00000
N 0.00000 0.68138 0.00000
C -1.12986 0.00429 0.00000
C -1.20429 -1.39002 0.00000
C -0.00004 -2.08758 0.00000
H 2.16964 -1.89551 0.00000

H -0.00009 -3.18011 0.00000
H -2.16970 -1.89546 0.00000
Cl 2.59764 0.92217 0.00000
Cl -2.59757 0.92232 0.00000

[(Cl_2-py)H]+
PBE0
SCF = -1166.79517461
H (0 K) = -1166.711308
H (398 K) = -1166.699118
G (298 K) = -1166.743218
G (398 K) = -1166.757092
Low freq. = 176.7734
Second freq. = 186.9696
B3PW91
SCF (PhCl,D3BJ,BS2) = -1167.88881253

12
[(Cl_2-py)H]+
C 1.19167 -0.03821 0.00000
C 1.21505 -1.42359 0.00000
N 0.00000 0.60428 0.00000
C -1.19167 -0.03822 0.00000
C -1.21507 -1.42359 0.00000
C -0.00000 -2.10801 0.00000
H 2.17375 -1.94300 0.00000
H -0.00000 -3.20104 0.00000
H -2.17376 -1.94301 0.00000
Cl 2.57777 0.92412 0.00000
Cl -2.57776 0.92412 0.00000
H -0.00001 1.62686 0.00000

$GeCl_4$
PBE0
SCF = -3916.28232734
H (0 K) = -3916.276513
H (398 K) = -3916.264713
G (298 K) = -3916.309085
G (398 K) = -3916.323253
Low freq. = 124.0627
Second freq. = 124.0928
B3PW91
SCF (PhCl,D3BJ,BS2) = -3917.91805544

5
$GeCl_4$
Ge 0.00000 0.00000 0.00000
Cl 0.00000 1.73491 1.22674
Cl 1.73491 -0.00000 -1.22674
Cl -1.73491 0.00000 -1.22674
Cl -0.00000 -1.73491 1.22674

$AlCl_3$
PBE0
SCF = -1622.17060799
H (0 K) = -1622.165669
G (298 K) = -1622.196641
G (398 K) = -1622.209518Low freq. = 146.8640
Second freq. = 146.9810
B3PW91
SCF (PhCl,D3BJ,BS2) = -1623.16167171

4
$AlCl_3$
Cl 1.65651 1.25507 0.00001
Al -0.00030 -0.00005 -0.00003
Cl 0.25893 -2.06181 0.00001
Cl -1.91522 0.80678 0.00001

$[AlCl_4]^-$
PBE0
SCF = -2082.25773737
H (0 K) = -2082.251656
G (298 K) = -2082.285248
G (398 K) = -2082.299728
Low freq. = 112.5276
Second freq. = 112.8405
B3PW91
SCF (PhCl,D3BJ,BS2) = -2083.58140629

5
[AlCl4]-
Al 0.00033 -0.00000 -0.00010
Cl 1.47520 0.97990 -1.23837
Cl -0.89874 1.44676 1.32949
Cl -1.53035 -0.87315 -1.25143
Cl 0.95364 -1.55351 1.16038

Al_2Cl_6
PBE0
SCF = -3244.40106802
H (0 K) = -3244.389837
H (398 K) = -3244.370694
G (298 K) = -3244.432027
G (398 K) = -3244.451418
Low freq. = 15.8622
Second freq. = 62.9637
B3PW91
SCF (PhCl,D3BJ,BS2) = -3246.37417508

8
Al_2Cl_6
Al 1.59996 -0.00001 -0.00001
Cl 2.59542 -1.82889 0.00000
Cl 0.00000 -0.00013 -1.62471
Cl 2.59513 1.82903 0.00000
Al -1.59996 -0.00001 0.00002
Cl -2.59542 -1.82889 -0.00001
Cl -0.00000 -0.00014 1.62471

Cl -2.59513 1.82903 -0.00000

$[Al_2Cl_7]^-$
PBE0
SCF = -3704.49046861
H (0 K) = -3704.478497
H (398 K) = -3704.456231
G (298 K) = -3704.525079
G (398 K) = -3704.546819
Low freq. = 20.0329
Second freq. = 23.4810
B3PW91
SCF (PhCl,D3BJ,BS2) = -3706.78480155

9
$[Al_2Cl_7]^-$
Al 1.92055 -0.00683 -0.02682
Al -1.92085 0.00697 -0.02657
Cl 2.33180 2.02523 0.43182
Cl 0.00009 0.00061 -1.29331
Cl 3.31447 -0.84400 -1.40770
Cl -3.31449 0.84198 -1.40896
Cl -2.33018 -2.02520 0.43303
Cl -1.52992 1.25395 1.64270
Cl 1.52846 -1.25267 1.64323

$(Cl_2\text{-}Py)AlCl_3$
PBE0
SCF = -2788.65771251
H (0 K) = -2788.580595
G (298 K) = -2788.625486
G (398 K) = -2788.646402
Low freq. = 2.1957
Second freq. = 38.7440
B3PW91
SCF (PhCl,D3BJ,BS2) = -2790.67084999

15
$(Cl_2\text{-}Py)AlCl_3$
C 1.39681 -1.32516 0.00949
C 2.78099 -1.46578 -0.02468
N 0.77138 -0.13147 0.01641
C 1.55214 0.96990 0.00849
C 2.94295 0.91839 -0.02545
C 3.56385 -0.32110 -0.04623
H 3.21036 -2.46679 -0.03118
H 4.65303 -0.39493 -0.07410
H 3.50433 1.85181 -0.03261
Cl 0.46344 -2.75063 0.05390
Cl 0.83291 2.51401 0.05066
Al -1.34015 0.13709 -0.00941
Cl -2.51677 -1.62116 -0.07611
Cl -1.49507 1.18168 -1.84996
Cl -1.56485 1.11671 1.85773

TS1
PBE0
SCF = -7160.67313992
H (0 K) = -7160.655690
H (398 K) = -7160.62401
G (298 K) = -7160.711027
G (398 K) = -7160.738265
Low freq. = -61.4287
Second freq. = 15.8313
B3PW91
SCF (PhCl,D3BJ,BS2) = -7164.27902342

13
TS1
Al -0.78469 1.73112 -0.06895
Al -2.73473 -1.06366 0.03092
Cl -1.50915 3.43486 0.90479
Cl -1.97551 0.47019 -1.52445
Cl 0.81310 2.25136 -1.37346
Cl -1.32017 -2.62151 0.00735
Cl -2.35905 0.33808 1.65205
Cl -4.74317 -1.46777 -0.37366
Cl 0.72460 0.30648 1.29636
Ge 2.38524 -0.48960 0.05326
Cl 1.62658 -1.16779 -1.78644
Cl 3.05770 -2.11049 1.23235
Cl 3.88652 0.97776 -0.10608

Int-1
PBE0
SCF = -7160.67772582
H (0 K) = -7160.660088
H (398 K) = -7160.627236
G (298 K) = -7160.716331
G (398 K) = -7160.744211
Low freq. = 28.3796
Second freq. = 34.6145
B3PW91
SCF (PhCl,D3BJ,BS2) = -7164.28714996

13
Int-1
Al 0.42112 2.03720 0.18505
Al 2.67675 -0.99435 -0.05320
Cl 1.27445 3.35669 -1.17186
Cl 1.79078 0.76663 1.34034
Cl -1.17844 2.62342 1.40274
Cl 1.13102 -2.43762 0.08610
Cl 2.76394 0.00777 -1.90903
Cl 4.46140 -1.40428 0.96501
Cl -0.61327 0.41997 -1.23415
Ge -2.18707 -0.66885 -0.07351
Cl -1.55319 -0.94544 1.90832
Cl -2.41962 -2.46477 -1.14349
Cl -3.90920 0.53916 -0.20644

TS2
PBE0
SCF = -7851.79072622
H (0 K) = -7851.680292
H (398 K) = -7851.636784
G (298 K) = -7851.744346
G (398 K) = -7851.777560
Low freq. = -31.7345
Second freq. = 20.3539
B3PW91
SCF (PhCl,D3BJ,BS2) = -7856.14136375

25
TS2
C 3.07323 2.10989 1.11375
C 1.97794 2.34264 0.26827
C 1.93305 1.70381 -0.95305
C 2.94368 0.75777 -1.31730
C 4.09250 0.62028 -0.47492
C 4.14304 1.27006 0.73544
Ge 1.80903 -1.05049 -0.85465
Cl 3.12538 -2.58802 -1.49542
Cl 1.69956 -1.04172 1.25096
Cl 0.24470 -0.78362 -2.23607
Cl -0.80678 -2.95308 0.02397
Al -1.93159 -1.62836 1.29613
Cl -1.25443 -1.71378 3.29900
Cl -3.99977 -1.63063 0.93379
Cl -1.22143 0.51124 0.71094
Al -2.54447 1.63697 -0.81413
Cl -4.17978 2.40986 0.25219
Cl -1.09927 3.14369 -1.36871
Cl -2.91541 0.26837 -2.38222
H 3.04116 0.48974 -2.37806
H 1.07371 1.89094 -1.60614
H 4.90385 -0.04543 -0.77834
H 1.16531 3.00988 0.56188
H 4.98759 1.14193 1.41365
Cl 3.12182 2.85946 2.63726

Int-2
PBE0
SCF = -4147.18871903
H (0 K) = -4147.091109
H (398 K) = -4147.070302
G (298 K) = -4147.132254
G (398 K) = -4147.151567
Low freq. = 41.0400
Second freq. = 46.1551
B3PW91
SCF (PhCl,D3BJ,BS2) = -4149.34272423

16
Int-2
C 2.64796 0.00000 -0.30732
C 2.04110 1.23421 -0.62992
C 0.80975 1.23626 -1.23775
C 0.11011 0.00004 -1.47871
C 0.80974 -1.23620 -1.23781
C 2.04110 -1.23418 -0.62999
Ge -1.27654 -0.00000 0.11641
Cl -0.18898 -0.00032 1.92394
Cl -2.42009 -1.75183 -0.15393
Cl -2.41971 1.75212 -0.15356
H 0.32675 2.18156 -1.49953
H 0.32675 -2.18149 -1.49965
H 2.56303 2.16517 -0.40216
H 2.56303 -2.16515 -0.40227
Cl 4.14422 -0.00002 0.47072
H -0.65148 0.00006 -2.27441

TS3
PBE0
SCF = -5313.66521161
H (0 K) = -5313.500804
H (398 K) = -5313.467216
G (298 K) = -5313.554775
G (398 K) = -5313.581730
Low freq. = -589.5726
Second freq. = 9.5671
B3PW91
SCF (PhCl,D3BJ,BS2) = -5316.81787109

27
TS3
N 1.74662 -1.09734 -0.00007
C 2.40878 -1.24644 1.14930
C 3.77467 -1.50099 1.20413
C 4.46191 -1.61563 -0.00004
C 3.77476 -1.50066 -1.20423
C 2.40886 -1.24612 -1.14943
Cl 1.51085 -1.15699 2.60824
Cl 1.51104 -1.15627 -2.60842
C -0.87143 -0.15278 -0.00005
C -1.50867 -0.54701 1.22406
C -2.63145 -1.34188 1.22815
C -3.18982 -1.74261 -0.00016
C -2.63144 -1.34172 -1.22841
C -1.50866 -0.54685 -1.22421
Ge -0.33971 1.75991 0.00012
Cl -2.09579 2.94523 0.00058
Cl 0.81507 2.14475 -1.74115
Cl 0.81562 2.14425 1.74113
H -1.07863 -0.22063 2.17414
H -1.07862 -0.22035 -2.17424
H -3.10140 -1.65611 2.16134
H -3.10138 -1.65583 -2.16164
Cl -4.57689 -2.71856 -0.00022
H 0.34509 -0.59677 -0.00006

H 5.53655 -1.81146 -0.00003
H 4.26984 -1.60946 -2.16926
H 4.26968 -1.61006 2.16917

p-GeCl3-C6H4Cl
PBE0
SCF = -4146.87952919
H (0 K) = -4146.793328
H (398 K) = -4146.772925
G (298 K) = -4146.835400
G (398 K) = -4146.854931
Low freq. = 17.7960
Second freq. = 46.3351
B3PW91
SCF (PhCl,D3BJ,BS2) = -4148.96281084

15
p-GeCl3-C6H4Cl
C 3.25273 -0.00001 0.00769
C 2.56694 -1.21504 -0.00170
C 1.17557 -1.21038 -0.01584
C 0.47451 0.00016 -0.01593
C 1.17571 1.21060 -0.01593
C 2.56710 1.21508 -0.00181
Ge -1.44524 0.00007 -0.00374
Cl -2.20148 1.74958 -0.98331
Cl -2.25630 -0.00601 1.97998
Cl -2.20139 -1.74374 -0.99351
H 0.63707 -2.16120 -0.03746
H 0.63726 2.16145 -0.03757
H 3.12441 -2.15281 -0.00274
H 3.12468 2.15279 -0.00293
Cl 4.97970 -0.00013 0.02399

TS2(Ph)
PBE0
SCF = -7392.48074037
H (0 K) = -7392.360603
G (298 K) = -7392.422568
Low freq. = -31.4502
Second freq. = 22.1881
B3PW91
SCF (PhCl,D3BJ,BS2) = -7396.56426135

25
TS2(Ph)
Al 1.65292 -2.09119 -0.46929
Al 2.52845 1.67047 0.26624
Cl 3.65741 -2.15372 0.15269
Cl 1.22313 0.15698 -0.89614
Cl 1.15291 -2.92828 -2.34859
Cl 1.27804 3.40366 -0.03686
Cl 2.51967 1.01514 2.27686
Cl 4.36749 1.78630 -0.74106
Cl 0.23101 -2.64818 1.04720
Ge -2.16700 -0.30206 0.65323
Cl -1.83476 -1.20038 -1.22042
Cl -0.73321 0.36056 2.04039
Cl -3.76423 -1.28499 1.64742
H -3.30367 1.83623 1.23578
C -3.07204 1.64865 0.17833
H -1.08293 2.58714 0.28042
H -5.05775 0.86286 -0.27094
C -1.88884 2.23050 -0.36964
C -4.13277 1.26013 -0.69492
C -1.72112 2.28925 -1.74387
C -3.95355 1.34096 -2.06158
C -2.74665 1.83972 -2.57928
H -0.78601 2.68511 -2.14536
H -4.74268 1.01242 -2.74026
H -2.60779 1.87596 -3.66300

Int2(Ph)
PBE0
SCF = -3687.87883727
H (0 K) = -3687.771591
G (298 K) = -3687.810518
Low freq. = 51.6067
Second freq. = 87.4527
B3PW91
SCF (PhCl,D3BJ,BS2) = -3689.76591147

16
Int2(Ph)
Ge 0.75061 -0.00005 -0.07268
Cl 1.84022 -1.74703 0.38017
Cl 1.83896 1.74790 0.37952
Cl -0.04297 -0.00040 -2.02280
C -0.85960 -0.00036 1.33176
C -1.48949 1.23725 0.96946
C -1.48966 -1.23775 0.96905
C -2.61237 1.22619 0.16508
H -1.04714 2.17923 1.30404
C -2.61258 -1.22629 0.16475
H -1.04747 -2.17991 1.30335
C -3.16041 0.00006 -0.24078
H -3.07881 2.16349 -0.14427
H -3.07919 -2.16342 -0.14485
H -4.04513 0.00022 -0.88373
H -0.19277 -0.00057 2.20833

TS3(Ph)
PBE0
SCF = -4854.35566290
H (0 K) = -4854.181386
G (298 K) = -4854.232719
Low freq. = -359.5752
Second freq. = 12.6689
B3PW91
SCF (PhCl,D3BJ,BS2) = -4857.24051359

27
TS3(Ph)
Ge 1.37716 -0.96915 -0.09062
Cl 0.56590 -1.71379 -1.90577
Cl 0.57627 -2.03731 1.56000
Cl 3.49026 -1.10732 -0.10977
C 0.88398 0.95321 0.08938
C 1.25403 1.47864 1.37041
C 1.25255 1.70623 -1.07301
C 1.88475 2.70242 1.47921
H 1.01765 0.90109 2.26733
C 1.88326 2.92913 -0.95476
H 1.01495 1.30424 -2.06086
C 2.19577 3.42292 0.31859
H 2.14900 3.10269 2.45983
H 2.14636 3.50362 -1.84491
H 2.70194 4.38811 0.40819
H -0.37318 0.71171 0.06718
Cl -1.68547 0.36240 2.65464
Cl -1.68866 0.85536 -2.53961
C -2.51326 0.14302 1.16745
C -3.82989 -0.30455 1.18155
C -4.48670 -0.42469 -0.03889
C -3.83123 -0.07706 -1.21568
C -2.51455 0.36002 -1.11917
N -1.86254 0.44377 0.04202
H -5.52038 -0.77628 -0.07168
H -4.31682 -0.13176 -2.19005
H -4.31438 -0.54161 2.12880

$PhGeCl_3$
PBE0
SCF = -3687.57028335
H (0 K) = -3687.474422
G (298 K) = -3687.514301
G (398 K) = -3687.532609
Low freq. = 18.6441
Second freq. = 63.6512
B3PW91
SCF (PhCl,D3BJ,BS2) = -3689.38498113

15
$PhGeCl_3$
Ge -0.79420 0.00009 -0.00560
Cl -1.61891 -0.00632 1.97415
Cl -1.55378 -1.74223 -0.99827
Cl -1.55383 1.74849 -0.98750
C 1.12604 0.00013 -0.00829
C 1.82266 -1.21312 -0.00354
C 1.82292 1.21320 -0.00363
C 3.21563 -1.20937 0.01954
H 1.27909 -2.16109 -0.02841
C 3.21590 1.20913 0.01944
H 1.27949 2.16124 -0.02849
C 3.91034 -0.00020 0.03315
H 3.76108 -2.15585 0.02213
H 3.76158 2.15548 0.02196
H 5.00282 -0.00032 0.04947

Ph_2GeCl_2
PBE0
SCF = -3458.85196002
H (0 K) = -3458.666031
G (298 K) = -3458.711044
Low freq. = 21.7196
Second freq. = 33.6080
B3PW91
SCF (PhCl,D3BJ,BS2) = -3460.84442417

25
Ph_2GeCl_2
C 3.92685 -1.87676 -0.22698
C 2.78954 -2.24553 -0.94507
C 1.63579 -1.46798 -0.86771
C 1.61639 -0.31480 -0.07231
C 2.75871 0.04725 0.64937
C 3.91105 -0.73242 0.56988
Ge 0.00000 0.73447 -0.00001
Cl 0.14156 2.04077 1.72095
C -1.61637 -0.31481 0.07228
Cl -0.14158 2.04086 -1.72089
H 0.74559 -1.76603 -1.42958
H 2.74157 0.93954 1.28051
H 2.80037 -3.14389 -1.56684
H 4.80122 -0.44606 1.13503
H 4.83117 -2.48724 -0.28717
C -1.63578 -1.46799 0.86770
C -2.78953 -2.24554 0.94507
C -3.92684 -1.87677 0.22697
C -3.91104 -0.73244 -0.56991
C -2.75870 0.04723 -0.64941
H -0.74558 -1.76603 1.42958
H -2.80037 -3.14389 1.56685
H -4.83115 -2.48725 0.28717
H -4.80121 -0.44609 -1.13506
H -2.74156 0.93951 -1.28055

Ph_3GeCl
PBE0
SCF = -3230.12724642
H (0 K) = -3229.851518
G (298 K) = -3229.903186
Low freq. = 9.2265
Second freq. = 19.2212
B3PW91
SCF (PhCl,D3BJ,BS2) = -3232.29712080

35
Ph_3GeCl
Ge 0.00262 -0.03142 0.53030
Cl -0.02836 -0.14675 2.71141
C -0.13025 1.82922 0.00745
C 0.96445 2.69034 0.16257
C -1.31726 2.32733 -0.54320
C 0.87126 4.02625 -0.22137
H 1.90027 2.31471 0.58669
C -1.41044 3.66440 -0.92849
H -2.17724 1.66397 -0.67407
C -0.31684 4.51360 -0.76716
H 1.72921 4.69126 -0.09529
H -2.34093 4.04464 -1.35722
H -0.38936 5.56141 -1.06909
C -1.53462 -1.03943 -0.09017
C -2.66962 -1.21152 0.71073
C -1.52713 -1.57176 -1.38611
C -3.78091 -1.89636 0.22101
H -2.67715 -0.81694 1.73047
C -2.63835 -2.25634 -1.87582
H -0.64248 -1.46177 -2.02095
C -3.76681 -2.41715 -1.07250
H -4.66163 -2.02774 0.85455
H -2.62143 -2.67045 -2.88697
H -4.63755 -2.95557 -1.45482
C 1.68882 -0.79093 -0.05126
C 2.24643 -0.37911 -1.26924
C 2.34359 -1.77450 0.69980
C 3.43148 -0.94956 -1.73369
H 1.75925 0.40331 -1.85934
C 3.52936 -2.34161 0.23734
H 1.92688 -2.08875 1.66062
C 4.07247 -1.93184 -0.98057
H 3.85868 -0.62098 -2.68435
H 4.03499 -3.10631 0.83215
H 5.00292 -2.37702 -1.34158

p-tolyl-$GeCl_3$
PBE0
SCF = -3726.81543858
H (0 K) = -3726.692393
G (298 K) = -3726.736141
Low freq. = 15.9620
Second freq. = 18.2190
B3PW91
SCF (PhCl,D3BJ,BS2) = -3728.70998662

18
p-tolyl-$GeCl_3$
Ge -1.13564 0.00007 -0.00354
Cl -1.95682 -0.00591 1.97893
Cl -1.90322 -1.74253 -0.99147
Cl -1.90341 1.74820 -0.98150
C 0.78124 0.00021 -0.01651
C 1.48412 -1.20893 -0.01840
C 1.48417 1.20922 -0.01849
C 2.87526 -1.20222 -0.00707
H 0.94609 -2.16009 -0.04249
C 2.87539 1.20240 -0.00716
H 0.94617 2.16039 -0.04260
C 3.59388 0.00010 0.00356
H 3.41682 -2.15204 -0.01375
H 3.41704 2.15215 -0.01392
C 5.09281 -0.00025 0.04903
H 5.44838 -0.00983 1.09297
H 5.51152 -0.88741 -0.44703
H 5.51160 0.89554 -0.43105

(p-tolyl)$_2$-$GeCl_2$
PBE0
SCF = -3537.34171928
H (0 K) = -3537.101422
G (298 K) = -3537.154084
Low freq. = 11.1310
Second freq. = 21.4427
B3PW91
SCF (PhCl,D3BJ,BS2) = -3539.49380042

31
(p-tolyl)$_2$-$GeCl_2$
C 3.95867 1.60485 0.13347
C 3.90228 0.44529 -0.64980
C 2.74831 -0.33076 -0.70514
C 1.61711 0.03501 0.03118
C 1.66267 1.19360 0.81647
C 2.82064 1.96425 0.86618
Ge -0.00028 -1.00961 -0.00122
Cl 0.10022 -2.31551 -1.72795
C 5.19420 2.45520 0.16291
C -1.61619 0.03753 -0.02791
Cl -0.10290 -2.32731 1.71631
H 2.72343 -1.22798 -1.32906
H 0.78821 1.50171 1.39740
H 4.78076 0.14488 -1.22774
H 2.84304 2.86416 1.48713
H 6.10133 1.85385 0.00696
H 5.16397 3.21491 -0.63613
H 5.29397 2.98973 1.11845
C -2.74468 -0.32519 0.71374
C -3.89541 0.45661 0.66763
C -3.95244 1.61635 -0.11497
C -2.81437 1.97670 -0.84755
C -1.65994 1.20057 -0.80700
H -2.71831 -1.22098 1.33963
H -4.76914 0.16292 1.25609
C -5.20580 2.43745 -0.18738
H -2.83260 2.88427 -1.45743
H -0.78440 1.51128 -1.38488

H -4.98278 3.50211 -0.34812
H -5.83974 2.10598 -1.02685
H -5.80366 2.34398 0.73044

Int-1($AlCl_3$)
PBE0
SCF = -5538.47254798
H (0 K) = -5538.460994
G (298 K) = -5538.507437
Low freq. = 24.9058
Second freq. = 35.1447
B3PW91
SCF (PhCl,D3BJ,BS2) = -5541.09597874

9
Int-1($AlCl_3$)Ge -1.58195 -0.00310 -0.07098
Cl -1.22738 1.48329 1.37700
Cl 0.17987 -0.14597 -1.38037
Cl -3.14488 0.56705 -1.37838
Cl -1.95240 -1.90624 0.75213Al 2.24805 -0.00296 0.07356
Cl 1.48745 -1.04509 1.73573
Cl 3.49395 -1.02311 -1.26497
Cl 2.42206 2.07816 0.23622
(Cl_2-Py)Al_2Cl_6
PBE0
SCF = -4410.87802144
H (0 K) = -4410.794675
G (298 K) = -4410.847474
G (398 K) = -4410.873853
Low freq. = 14.8880
Second freq. = 45.5214
B3PW91
SCF (PhCl,D3BJ,BS2) = -4413.87324599
19
(Cl_2-Py)Al_2Cl_6
Al -1.15066 1.27463 0.50052
Al 2.35790 0.10225 -0.31832
Cl -1.22722 2.69675 -1.03778
Cl 1.00714 1.33863 1.14816
Cl -2.46100 1.60235 2.10984
Cl 2.57197 -1.74160 0.71292
Cl 1.12384 -0.08592 -2.04327
Cl 4.07596 1.29314 -0.49795
Cl -2.62802 0.00472 -2.14104
Cl -0.50352 -1.19332 2.49071
C -1.80651 -1.11508 -1.15762
C -1.65881 -2.41863 -1.60791
C -1.05101 -3.34344 -0.76955
C -0.67095 -2.95914 0.50782
C -0.86626 -1.63814 0.88786
N -1.36264 -0.69992 0.04961
H -0.89717 -4.37174 -1.10296
H -0.22401 -3.65218 1.21859
H -2.01861 -2.67986 -2.60210
[(Cl_2-Py)$_2$$AlCl_2$]$^+$
PBE0
SCF = -3494.95573003
H (0 K) = -3494.807276
G (298 K) = -3494.858553
G (398 K) = -3494.884249
Low freq. = 17.5920
Second freq. = 31.4812
B3PW91
SCF (PhCl,D3BJ,BS2) = -3497.75620642

25
[(Cl_2-Py)$_2$$AlCl_2$]$^+$
Cl 0.03681 -1.30763 2.10498
Cl 0.28601 2.19175 -1.76417
Cl -0.03682 -1.31409 -2.10106
Al 0.00013 1.09008 -0.00139
Cl -0.28619 2.19700 1.75802
Cl -2.86110 1.18982 -1.45859
Cl 2.86109 1.19434 1.45470
N -1.54355 -0.20507 0.31792
N 1.54357 -0.20611 -0.31739
C 2.73491 -0.04457 0.29600
C -2.73484 -0.04546 -0.29604
C -1.45061 -1.15232 1.27264
C 1.45061 -1.15622 -1.26924
C 2.50300 -1.99080 -1.61366
H 2.36164 -2.74098 -2.39164
C -3.71641 -1.82224 0.95974
H -4.56844 -2.45831 1.21058
C -3.84137 -0.83510 -0.00929
H -4.77630 -0.65671 -0.54061
C 3.71646 -1.82510 -0.95442
H 4.56851 -2.46189 -1.20336
C 3.84144 -0.83504 0.01163
H 4.77639 -0.65503 0.54238
C -2.50295 -1.98592 1.61952
H -2.36158 -2.73378 2.39973

S5. References

[1] A. El-Maradny, H. Tobita, H. Ogino, "Photoreaction of Silyliron(II) Complex $Cp^*Fe(CO)_2$ $SiMe_3$ (Cp^* = η^5-C_5Me_5) in the Presence of *p*-Tolylgermane" *Organometallics* **1996**, *15*, 4954–4958.

[2] P. Schmid, B. Bitschnau, M. Finšgar, I. Letofsky-Papst, J. Rattenberger, R. Saf, F. Uhlig, A. Torvisco, "Characterization of Germanium Nanoparticles from Arylgermanium Trihydrides" *Chem. Eur. J.* **2024**, *30*, e202401382.

[3] C. Fricke, G. J. Sherborne, I. Funes-Ardoiz, E. Senol, S. Guven, F. Schoenebeck, "Orthogonal Nanoparticle Catalysis with Organogermanes" *Angew. Chem. Int. Ed.* **2019**, *58*, 17788–17795.

[4] A. Kaithal, H. S. Sasmal, S. Dutta, F. Schäfer, L. Schlichter, F. Glorius, "cis-Selective Hydrogenation of Aryl Germanes: A Direct Approach to Access Saturated Carbo- and Heterocyclic Germanes" *J. Am. Chem. Soc.* **2023**, *145*, 4109–4118.

[5] C. Fricke, K. Deckers, F. Schoenebeck, "Orthogonal Stability and Reactivity of Aryl Germanes Enables Rapid and Selective (Multi)Halogenations" *Angew. Chem. Int. Ed.* **2020**, *59*, 18717–18722.

[6] C. Fricke, A. Dahiya, W. B. Reid, F. Schoenebeck, "Gold-Catalyzed C–H Functionalization with Aryl Germanes" *ACS Catal.* **2019**, *9*, 9231–9236.

[7] A. V. Schellbach, D. R. Willcox, M. Guarnaccia, G. S. Nichol, V. Fasano, M. J. Ingleson, "Intermolecular Sp3C─H Metalation of Non-Nucleophilic Brønsted Bases Using Simple Lewis Acids" *Angew. Chem. Int. Ed.* **2025**, *64*, e202512254.

[8] M. J. Frisch, G. W. Trucks, H. B. Schlegel, G. E. Scuseria, M. A. Robb, J. R. Cheeseman, G. Scalmani, V. Barone, G. A. Petersson, H. Nakatsuji, X. Li, M. Caricato, A. V. Marenich, J. Bloino, B. G. Janesko, R. Gomperts, B. Mennucci, H. P. Hratchian, J. V. Ortiz, A. F. Izmaylov, J. L. Sonnenberg, D. Williams-Young, F. Ding, F. Lipparini, F. Egidi, J. Goings, B. Peng, A. Petrone, T. Henderson, D. Ranasinghe, V. G. Zakrzewski, J. Gao, N. Rega, G. Zheng, W. Liang, M. Hada, M. Ehara, K. Toyota, R. Fukuda, J. Hasegawa, M. Ishida, T. Nakajima, Y. Honda, O. Kitao, H. Nakai, T. Vreven, K. Throssell, J. J. A. Montgomery, J. E. Peralta, F. Ogliaro, M. J. Bearpark, J. J. Heyd, E. N. Brothers, K. N. Kudin, V. N. Staroverov, T. A. Keith, R. Kobayashi, J. Normand, K. Raghavachari, A. P. Rendell, J. C. Burant, S. S. Iyengar, J. Tomasi, M. Cossi, J. M. Millam, M. Klene, C. Adamo, R. Cammi, J. W. Ochterski, R. L. Martin, K. Morokuma, O. Farkas, J. B. Foresman, D. J. Fox **2016**.

[9] S. Grimme, S. Ehrlich, L. Goerigk *J. Comput. Chem.* **2011**, *32*, 1456–1465.

[10] F. Weigend, R. Ahlrichs, "Balanced basis sets of split valence, triple zeta valence and quadruple zeta valence quality for H to Rn: Design and assessment of accuracy" *Phys. Chem. Chem. Phys.* **2005**, *7*, 3297–3305.

[11] F. Weigend *Phys. Chem. Chem. Phys.* **2006**, *8*, 1057–1065.

[12] A. D. Becke *J. Chem. Phys.* **1993**, *98*, 5648–5652.

[13] S. Grimme, J. Antony, S. Ehrlich, H. Krieg *J. Chem. Phys.* **2010**, *132*, 154104.

[14] A. V. Marenich, C. J. Cramer, D. G. Truhlar, "Universal Solvation Model Based on Solute Electron Density and on a Continuum Model of the Solvent Defined by the Bulk Dielectric Constant and Atomic Surface Tensions" *J. Phys. Chem. B* **2009**, *113*, 6378–6396.

[15] A. D. Becke *Phys. Rev. A* **1988**, *38*, 3098–3100.

[16] J. P. Perdew *Phys. Rev. B* **1986**, *33*, 8822–8824.

[17] C. Lee, W. Yang, R. G. Parr *Phys. Rev. B* **1988**, *37*, 785–789.

[18] S. Grimme, "Semiempirical GGA-type density functional constructed with a long-range dispersion correction" *J. Comput. Chem.* **2006**, *27*, 1787–1799.

[19] C. Adamo, V. Barone *J. Chem. Phys.* **1999**, *110*, 6158–6170.
[20] Y. Zhao, N. E. Schultz, D. G. Truhlar, "Exchange-correlation functional with broad accuracy for metallic and nonmetallic compounds, kinetics, and noncovalent interactions" *J. Chem. Phys.* **2005**, *123*, 161103.
[21] Y. Zhao, N. E. Schultz, D. G. Truhlar, "Design of Density Functionals by Combining the Method of Constraint Satisfaction with Parametrization for Thermochemistry, Thermochemical Kinetics, and Noncovalent Interactions" *J. Chem. Theory Comput.* **2006**, *2*, 364–382.
[22] Y. Zhao, D. G. Truhlar *Theor. Chem. Acc.* **2008**, *120*, 215–241.
[23] H. S. Yu, X. He, S. L. Li, D. G. Truhlar, "MN15: A Kohn–Sham global-hybrid exchange–correlation density functional with broad accuracy for multi-reference and single-reference systems and noncovalent interactions" *Chem. Sci.* **2016**, *7*, 5032–5051.
[24] J. D. Chai, M. Head-Gordon *Phys. Chem. Chem. Phys.* **2008**, *10*, 6615–6620.
[25] J. Tao, J. P. Perdew, V. N. Staroverov, G. E. Scuseria *Phys. Rev. Lett.* **2003**, *91*, 146401.
[26] M. K. Bisai, J. Łosiewicz, L. Sotorrios, G. S. Nichol, A. P. Dominey, M. J. Cowley, S. P. Thomas, S. A. Macgregor, M. J. Ingleson, "Transition Metal-Free Catalytic C−H Zincation and Alumination" *Angew. Chem. Int. Ed.* **2024**, *63*, e202404848.
[27] P. Ríos, A. Rodríguez and J. López-Serrano, ACS Catal., **2016**, *6*, 5715–5723.
[28] J. Kua, H. E. Krizner and D. O. De Haan, J. Phys. Chem. A, **2011**, *115*, 1667–1675.
